# Use of a Facilitated Group Process to Design and Implement a Group Antenatal and Postnatal Care Program in Rwanda

**DOI:** 10.1111/jmwh.12871

**Published:** 2018-09-25

**Authors:** Felix Sayinzoga, Tiffany Lundeen, Mathias Gakwerere, Emmanuel Manzi, Yvonne Delphine U. Nsaba, M. Providence Umuziga, Ina R. Kalisa, Sabine F. Musange, Dilys Walker

**Keywords:** antenatal care, group prenatal care, CenteringPregnancy, postnatal care, global health/international

## Abstract

**Introduction:**

The government of Rwanda is exploring strategies that may reduce the incidence of prematurity and low birth weight. Large‐scale implementation of group antenatal care (ANC) and postnatal care (PNC) within the context of the Rwanda national health care system is under consideration. To launch a cluster randomized controlled trial of group ANC and PNC in 5 districts in Rwanda, the implementation team needed a customized group care model for this context and trained health care workers to deliver the program.

**Process:**

Adapting the group ANC and group PNC model for the Rwandan context was accomplished through a group process identical to that which is fundamental to group care. A technical working group composed of 10 Rwandan maternal‐child health stakeholders met 3 times over the course of 3 months, for 4 to 8 hours each time. Their objectives were to consider the evidence on group ANC, agree on the priorities and constraints of their ANC delivery system, and ultimately define the content and structure of a combined group ANC and PNC model for implementation in Rwanda. The same group process was employed to train health care workers to act as group ANC facilitators.

**Outcomes:**

A customized group ANC and PNC model and guidelines for its introduction were developed in the context of a cluster randomized controlled trial in 36 health centers. Descriptions of this model and the implementation plan are included in this article.

**Discussion:**

Our experience suggests that the group process fundamental to successful group ANC and PNC is an effective method to customize and implement this innovative health services delivery model in a new context and is instrumental in achieving local ownership.

## INTRODUCTION

In 2015, the East Africa Preterm Birth Initiative‐Rwanda, a partnership among the Rwanda Ministry of Health, the Rwanda Biomedical Center, the University of Rwanda School of Public Health, and the University of California, San Francisco, Institute of Global Health Sciences, decided to design and implement a cluster randomized controlled trial of group antenatal care (ANC) and postnatal care (PNC) powered to assess the impact of this model of care on gestational age at birth in 5 districts. This article describes the facilitated group process central to developing a group care model and its implementation plan in the context of this study.

### Background

In the last decade, Rwanda has significantly improved its maternal‐child health indicators; the maternal mortality ratio, neonatal mortality rate, and infant mortality rate have all plummeted. Between 2005 and 2015, the maternal mortality ratio fell from 750 pregnancy‐related deaths per 100,000 live births to 210 deaths per 100,000 live births, the neonatal mortality rate decreased from 37 neonatal deaths per 1000 live births to 14 neonatal deaths per 1000 live births, and the infant mortality rate fell from 86 deaths per 1000 live births to 32 deaths per 1000 live births.^1^, ^2^ In Rwanda, neonatal mortality represents 64% of infant mortality and 45% of overall under‐5 mortality; prematurity is associated with 48% of all neonatal deaths, and low birth weight is associated with 60% of neonatal deaths.^3^ These figures suggest that Rwanda, in order to achieve the ambitious Sustainable Development Goals by 2030, has further work to do to reduce the maternal mortality ratio to at least as low as 70 per 100,000 live births, the neonatal mortality rate to at least as low as 12 per 1000 live births, and the under‐5 mortality to at least as low as 25 per 1000 live births.^4^

The Rwanda public health care delivery system is a pyramidal structure composed of 8 national referral hospitals, 4 provincial hospitals, 35 district hospitals in 30 districts, 495 health centers, and 406 health posts^1^ and includes a network of approximately 45,000 community health workers (CHWs) at the village level.^5^ The community health program has been implemented nationwide and represents the first line of service. Each village has a *binome*, a male‐female CHW pair who provide basic care, and a CHW called the *Animatrice de Santé Maternelle* who completes specific activities related to maternal and newborn health from pregnancy until the infant is 2 months of age. The first facility point of contact for clients are health centers that provide a minimum package of services spanning promotional, preventative, and curative activities. These public health centers are where universal access to ANC and PNC is offered in Rwanda. Health posts are facilities that bridge gaps between some health centers and communities by offering a more limited package of primary care services and are used intermittently for outreach

**QUICK POINTS**

✦ To customize a group antenatal care model for implementation and evaluation in a cluster randomized controlled trial in Rwanda, a stakeholder group applied the first principle of the group care model and facilitated discussion among the circle of group members.
✦ The resulting group antenatal and postnatal care program was shaped by national health system priorities and constraints as well as published literature about group antenatal care.
✦ The same group process was employed to train 6 Rwandan group care Master Trainers and 70 nurses and midwives and 217 community health workers as new group care facilitators.
✦ A facilitated group process will continue to be used as stakeholders revise and refine the Rwandan group care model.

activities. All Rwandans are invited to enroll in *Mutuelles de santé*, a universal, community‐based health insurance program that includes a household subscription and co‐payments at the time of care, including for ANC and PNC visits.^6^

Pregnant women and their newborns require access to high‐quality services along a continuum of care from pregnancy through childbirth and the postnatal period to help reduce maternal and newborn mortality. In Rwanda, there is large variation in the use of these services. The 2015 Rwanda Demographic and Health Survey reported nearly all pregnant women attended at least one ANC visit, but 56% of pregnant women did not receive 4 standard ANC visits as recommended in the national care package, and only 56% of women entered ANC by 16 weeks since their last menstrual period. Furthermore, whereas 91% of births occurred in health facilities in 2015, only 19% of newborns received PNC in the first 2 days after birth as recommended in government guidelines.^1^ Sustaining the momentum of newborn survival requires a renewed effort to build strong social networks, appropriate services and linkages to provide high‐quality ANC, skilled birth attendants, and PNC for women and newborns.

Group ANC has demonstrated promise as a service delivery model that may be superior to standard ANC in sociodemographic populations at disparately high risk for poor perinatal outcomes, with no report of harms. Randomized controlled trials demonstrating improved outcomes among women and newborns after participation in group ANC compared with standard ANC have been conducted in the United States^7^, ^8^, ^9^, ^10^, ^11^, ^12^ and Sweden,^13^ which the World Bank classifies as high‐income economies, and Iran,^14^, ^15^ which is classified as an upper middle‐income economy. Published benefits include increased uptake of postnatal family planning services, greater gestational age at birth and birth weight, lower incidence of sexually transmitted infections, healthier maternal weight trajectories, fewer depressive symptoms, and increased satisfaction with care.^16^ The potential to realize any of these benefits in the Rwandan maternity care context, especially increased gestational age at birth that might result in increased neonatal survival, inspired the East Africa Preterm Birth Initiative‐Rwanda to study group ANC at an expanded scale.

### Group ANC

Group ANC is an innovative model in which a group of pregnant women attend ANC visits together over the course of pregnancy; these women actively participate in their own physical assessments, discussion of pregnancy‐related topics, and mutual support. Sharon Schindler Rising, CNM, MSN, a nurse‐midwife based in the United States, formulated the conceptual basis for this model starting in the 1970s, named it CenteringPregnancy, and studied it for the first time in 1993 in a US hospital antenatal clinic among low‐income women.^17^ Rising and her colleagues defined CenteringPregnancy as “13 Essential Elements,” or key components, that correlate with the Institute of Medicine's 2001 suggestions for redesigning health care to improve quality.^18^, ^19^ CenteringPregnancy is now a trademarked package of tools, materials, and curricula administered by the Centering Healthcare Institute, a nonprofit organization.^20^

#### Past Efforts to Customize the Group ANC Model

Although the trademarked CenteringPregnancy package has been implemented in many locations,^20^ the core concepts of the group ANC model have been adapted and implemented in other ANC delivery sites without CenteringPregnancy's proprietary materials. Few published reports describe in detail the process by which these unique adaptations of the group ANC model have been made.

Two published examples of customization of the group ANC model for implementation in high‐income countries are summarized here: one is an example of a collaboration with Centering Healthcare Institute, and one is an example of a project that is independent of Centering Healthcare Institute. Kings College Hospital in London began with CenteringPregnancy training and tools as it launched its own group ANC program. In cooperation with the Centering Healthcare Institute, the local project leaders relied on action research to adapt CenteringPregnancy materials over the course of several group visits (with pregnant women) to reflect the local context of care; these changes included eliminating routine follow‐up weight measurements and reducing the number of group ANC visits to be consistent with the United Kingdom's National Institute of Health and Care Excellence's ANC guidelines.^21^ Expect With Me, a group ANC package designed for national scale in the United States, was implemented in 2014 across 5 facilities in 3 states with an interactive web‐based information platform for participants and health care providers. The authors reported that Expect With Me was developed using a human‐centered design approach with iterative revisions by a team whose special expertise is described, but there is no mention of a relationship with Centering Pregnancy.^22^

Published reports of the process by which group ANC programs have been designed for care delivery systems in low‐ and middle‐income countries, and who has participated in that process, are limited. Authors from Iran^15^ and Bangladesh^23^ did not describe the design process or specifications of their own group models of care in their published reports. In Ghana, a curriculum called *Home Based Life Saving Skills*, developed by the American College of Nurse‐Midwives, was adapted and delivered to women in 7 group ANC visits at a district hospital.^24^ Context‐specific constraints, such as government policies that prioritized precise gestational age at the time of each ANC visit over group consistency, shaped a group ANC model implemented in Nepal.^25^ A team that piloted group ANC in Malawi and Tanzania worked in cooperation with the Centering Healthcare Institute as they adapted CenteringPregnancy by reducing the total number of visits and incorporating region‐specific health promotion content; however, the process used for this adaptation was not described in detail in publications.^25^, ^26^

This report describes the process undertaken to develop a group care model for implementation and evaluation by the East Africa Preterm Birth Initiative‐Rwanda in the context of a cluster randomized controlled trial. We did not use tools, materials, or curricula trademarked as CenteringPregnancy. We relied exclusively on reports of CenteringPregnancy in peer‐reviewed journal articles and applied that knowledge to an action research plan inspired by the group care intervention itself.

## PROCESS

This work is included in the study protocol for a cluster randomized controlled trial of group ANC and PNC in Rwanda, which was approved by the Rwanda National Ethics Committee and the University of California, San Francisco, Institutional Review Board. The study is registered as NCT03154177 at http://clinicaltrials.gov.

Motivated by Kleinman's application of the social construction of reality theory to global health programs, we began with the assumption that even if the material facts of pregnancy should be consistent throughout the world, the reality of ANC and PNC is created socially and culturally through “legitimated ideas, practices, and things,”^27^ and that delivery of those services depends on a local moral context that influences the behaviors of health system administrators, health care providers, and health care seekers.

How could a well‐defined intervention, group ANC, be reimagined within a unique reality and retain its therapeutic potential? We relied on the fundamental principle of group ANC, the group process in which all members participate fully as peers and equals while 1 to 2 members act as facilitators.^28^ Descriptions of group ANC published over the past 20 years describe the role of a facilitative leader who understands that pregnant women in the group are the experts on their own needs.^19^ Quantitative and qualitative research on group ANC consistently demonstrates that a successful group process depends equally on the skill of the facilitator(s) and the engagement of all group members, which is physically represented and catalyzed by arranging all seats in a circle.^25^, ^29^, ^30^

**Figure 1:**
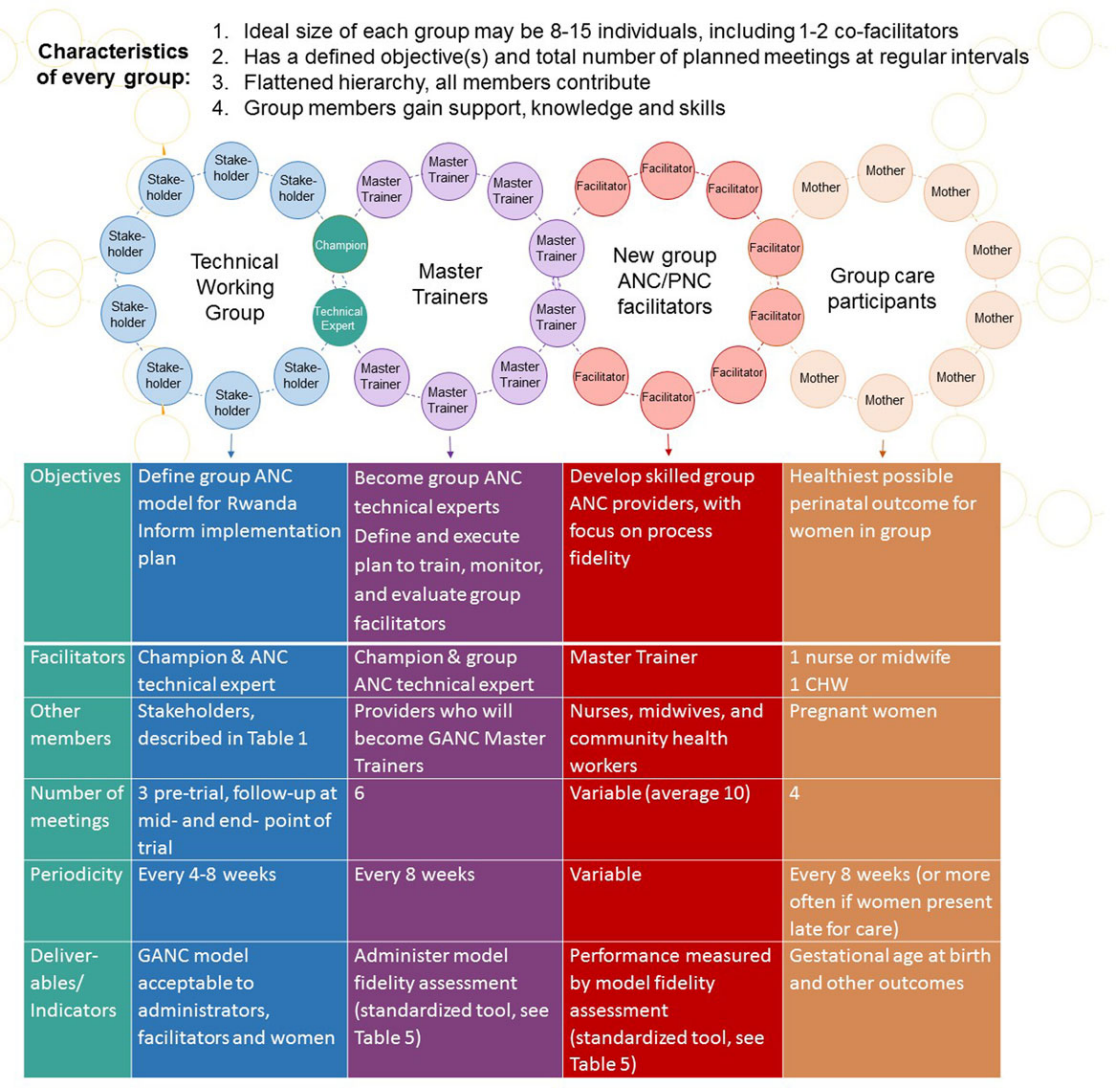
Conceptual Model for Implementing Group Antenatal and Postnatal Care in a New Context. A graphic representation of interrelated group processes employed for each design and implementation task.

We decided that this first principle of the group ANC model, facilitated discussion among group members seated in a circle, would inform our approach to accomplishing each of the tasks necessary to deliver group ANC in Rwanda. Our 3 main tasks were to 1) customize a group ANC model for this context; 2) build a cohort of group ANC Master Trainers to prepare, support, and monitor new group care facilitators; and 3) train a cadre of new group ANC facilitators. We applied principles of the group process to each of these tasks, represented in the conceptual framework illustrated in Figure 1. This figure represents the series of repeating, interlocking group processes that successfully resulted in the large‐scale implementation of group ANC among pregnant Rwandan women. Each circle represents a group of 8 to 15 individuals who work together to accomplish a defined objective within a prescribed amount of time, through a prescheduled series of group meetings.

Adapting the group care model for this context was accomplished in 2016 by a technical working group, described in Table 1. This group included 10 Rwandan stakeholders, the East Africa Preterm Birth Initiative‐Rwanda co‐Principal Investigator (the group ANC champion in this context), and a group ANC technical expert from outside Rwanda. This technical working group met 3 times over the course of 3 months, for 4 to 8 hours each time, and used the same group process. The group's objectives were to consider the evidence on group ANC and PNC, agree on the priorities and constraints of the Rwandan ANC and PNC delivery context, and ultimately define a group ANC and PNC model for implementation in Rwanda. Initial implementation was planned for 18 health centers, but the model was designed with large‐scale replication in mind.

**Table 1:** Composition of Technical Working Group on Rwandan Group Antenatal and Postnatal Care

Profession	Position and/or Institutional Affiliation	Nationality
Midwife	Assistant lecturer and clinical instructor, Kabgayi School of Nursing and Midwifery	Rwanda
Community health nurse	Director, community health unit, Rwanda Biomedical Center, Ministry of Health	Rwanda
General physician	Division manager, maternal, child, and community health division, Rwanda Biomedical Center	Rwanda
General physician	Specialist, maternal and child health, Ministry of Health	Rwanda
Mental health nurse	Lecturer, University of Rwanda School of Nursing (specialty: maternal mental health)	Rwanda
General physician	National program officer, maternal health and midwifery, United Nations Population Fund Rwanda	Rwanda
Obstetrician‐gynecologist	Lecturer, University of Rwanda College of Medicine and Health Sciences Clinical Instructor, Kigali University Teaching Hospital	Rwanda
Pediatrician	Newborn technical advisor, Maternal and Child Survival Program	Rwanda
Radiologist	Rwanda Radiologists Society	Rwanda
General physician	Public health specialist, maternal and child health, United Nations Children's Fund Rwanda	Rwanda
General physician	Lecturer, University of Rwanda School of Public Health Principal Investigator, East Africa Preterm Birth Initiative‐Rwanda	Rwanda
Midwife	University of California, San Francisco, Institute of Global Health Sciences	United States

We recruited a team of 5 Rwandan midwives and one physician, based on their professional reputation for excellent facilitation skills and interest in the project, to be part‐time group care Master Trainers. We decided on 6 Master Trainers because those selected have full‐time jobs and each had limited time to commit to training and field visits. The group ANC technical expert and the East Africa Preterm Birth Initiative‐Rwanda co‐Principal Investigator, acting as the group's cofacilitators, led this group of 6 Master Trainers through a purposeful group process. Half of the Master Trainers traveled to San Francisco, California, and each observed 3 to 4 actual group care sessions in various midwifery and family medicine practices; this experience was key to developing their confidence and understanding of the model. The Master Trainer group then met in Rwanda every 4 to 8 weeks for 6 follow‐up meetings to achieve group ANC and PNC technical expertise, develop training and monitoring plans for new group ANC and PNC facilitators, and coordinate their activities.

Health centers randomized to group ANC and PNC were invited to send all their health care providers who deliver ANC and/or PNC (usually 2‐3 per facility) to a 3‐day training workshop, in the capital city, on a rotating basis. The majority of these health care providers are nurses, but some have qualified as midwives. Twelve CHWs specializing in maternal‐child care within each of these health centers’ catchment areas were invited to this same training workshop. These new group care facilitators were trained using the same group process. Each training group consisted of 15 new facilitators and one group ANC Master Trainer and convened with the objective that each new facilitator would acquire the skills necessary to deliver group care, especially facilitation skills. Each of these groups met for 3 days of intensive training. The new group care facilitators received intermittent support visits from Master Trainers, convening a mini‐circle before and after the group visit to prepare, debrief, and discuss their progress as effective group ANC and PNC facilitators. Master Trainers collected data about model fidelity at each supportive visit using a tool represented in the online supplementary materials (see Supporting Information: Appendix S1).

In summary, the group process fundamental to group ANC has been applied to accomplish each task necessary for large‐scale implementation, from model design to service delivery. Figure 1 further explains how each group requires a distinct plan, including members, objective(s), a defined number of meetings at predetermined intervals, and deliverables or evaluation measures. Although each group has a unique membership, purpose, and schedule of meetings, the process by which the members accomplish their shared goal is the same. The facilitators lead group members through semistructured activities meant to generate cohesiveness, trust, and productive discussion, always with the end objective(s) in mind but without favoring their own opinions or agenda. Group members are encouraged to abandon real or perceived hierarchies, “listen, sort, and speak without having to be right,”^31^ and make decisions by consensus.

## OUTCOMES

A point‐by‐point comparison between the key components of the group ANC and PNC models defined by the technical working group (called *Ibaruke Neza Mubyeyi* in Rwanda) and CenteringPregnancy is reported in Table 2. There were 2 important logistical considerations related to the context of this trial. First, the health facilities randomized to group care entirely shifted to group ANC and group PNC as the standard of care for all women seeking ANC and PNC services (individual visits are available for those who opt out of group care or have special needs). Second, at the 18 health centers selected as study sites for this trial, existing health center staff were recruited to participate as group ANC and PNC facilitators. The nurses and midwives who provide ANC and PNC rotate through multiple service units at the health center, and although continuity of group ANC provider could not be guaranteed, one CHW is invited to consistently cofacilitate each longitudinal group. The technical working group integrated these realities with their vision for long‐term sustainability of a scaled group care model in Rwanda after the trial.

**Table 2:** Comparison of Key Components: *Ibaruke Neza Mubyeyi* a and CenteringPregnancy

CenteringPregnancy Essential Elements**^19^**	Does *Ibaruke Neza Mubyeyi* Include This Component?	Notes
Health assessment occurs within the group space.	Yes	Facilitators teach women in the first group antenatal care visit to assess weight and blood pressure (using an electronic cuff) with one another.
Women are involved in self‐care activities.	Yes	
A facilitative leadership style is used.	Yes	
Each session has an overall plan.	Yes	The facilitator's manual contains a plan for each session but encourages discussion of women's special concerns and questions.
Attention is given to the core content; emphasis may vary.	Yes	
There is stability of group leadership.	Mixed	A single community health worker attends all longitudinal visits and acts as a cofacilitator. The other cofacilitator is the antenatal care provider (nurse or midwife) on duty the day of a scheduled group visit; this person is not consistent across all of a distinct group's visits because of the needs of the health center's rotating staff schedule.
Group conduct honors the contribution of each member.	Yes	
The group is conducted in a circle.	Yes	
Group composition is stable but not rigid.	Yes	Women who miss scheduled group visits are encouraged to drop in and join other groups when they are able.
Group size is optimal to promote the process.	Yes	Recommended group size is 8‐12; in practice, actual group size ranges from 2‐16.
Involvement of family support people is optional.	Yes	Each group of women decides for themselves if they will invite husbands and next‐of‐kin to attend group visits.
Opportunity for socialization within the group is provided.	Yes	Some women continue to socialize outside of group antenatal care and have visited each other at home.
There is ongoing evaluation of outcomes.	Yes	Cofacilitators debrief after every group visit in a continuous learning and quality improvement process. Master Trainers regularly observe group visits, assess for model fidelity, and mentor cofacilitators. Health care provider and participant experiences will be measured in this study. Outcomes of the trial will be reported in 2019.

a *Ibaruke Neza Mubyeyi* is the name of the Rwandan group ANC and PNC model. It is translated from Kinyarwanda to English as “May all of us mothers have safe pregnancies, births, and new motherhood.”

The technical working group made several practical decisions that informed the group care model. First, the total number of routine antenatal visits is 4, following the focused ANC model that is foundational to the Rwanda ANC package. Notwithstanding the publication of new ANC recommendations by the World Health Organization during the model development phase, the technical working group felt an increase in the number of ANC contacts to 8 would place an impossible immediate strain on the ANC delivery system. Second, group care visits are spaced at regular 8‐week intervals to facilitate the creation of predictable group visit schedules at each health center, but if women present for their first ANC after mid‐pregnancy or miss their appointments, they are encouraged to drop in to other group visits at more narrow intervals to achieve 4 total ANC visits. Third, in consideration of the limited time both health care providers and women have to spend engaged in care, the time allowed for discussion and activities during the group visit was limited to 60 minutes. Fourth, the technical working group chose the discussion topics that should be addressed at each group visit, including the 6‐week postnatal visit (Table 3). Content decisions were based on the core content recommended in the Rwanda ANC and PNC packages as well as the area expertise of technical working group members. Fifth, women with any high‐risk conditions referred for more specialized care continued to attend routine group ANC (in addition to their specialty pregnancy surveillance) if their supervising physicians agreed to this parallel care. Finally, reuniting women at the 6‐week postnatal (postpartum) visit was prioritized to encourage uptake of PNC services.

**Table 3:** *Ibaruke Neza Mubyeyi* a Timing of Visits and Curriculum Content

Visit	Timing	Educational Content
Antenatal care visit 1 (standard, one‐on‐one initial pregnancy visit)	Variable; ideal is before 16 weeks’ gestation	Standard (eg, HIV counseling and testing) Introduction to group care model and invitation to participate
Antenatal care visit 2 (1st group visit)	20‐24 weeks’ gestation	Nutrition, supplements, and harmful substances Pregnancy danger signs Infection prevention and treatment
Antenatal care visit 3 (2nd group visit)	28‐32 weeks’ gestation	Birth plan (includes signs of labor) Healthy birth spacing and family planning Maternal mental health Review pregnancy danger signs
Antenatal care visit 4 (3rd group visit)	36‐40 weeks’ gestation	Respectful maternity care Breastfeeding and newborn care Postnatal and newborn danger signs Review family planning Review pregnancy danger signs
Postnatal care visit (4th group visit)	Approximately 6 weeks after birth	Review breastfeeding and infant feeding Review newborn danger signs Preventing health problems (eg, family planning, use of insecticide‐treated nets, hygiene, immunizations) Newborn and infant cognitive development (sing, talk, read, play)

a *Ibaruke Neza Mubyeyi* is the name of the Rwanda group ANC and PNC model. It is translated from Kinyarwanda to English as “May all of us mothers have safe pregnancies, births, and new motherhood.”

The technical working group prioritized the comfort of participants. They encouraged health center staff to provide clean drinking water for women during the group visit. The protection of each participant's confidential personal information was a priority for technical working group members, and some were fearful that women's privacy would be compromised to an unacceptable degree. As a result, each group visit began with a review of confidentiality and other group rules that the women set for themselves. The most common group rules the women set were to share phone numbers among themselves, arrive at the group visit on time, treat one another politely and with respect, set phones to silent, not go in and out of the room during group discussion, and protect one another's confidential, personal information; interestingly, most but not all groups decided (by consensus) not to invite husbands or next‐of‐kin to their sessions. The technical working group recommended that all group visits should be convened in an interior space of the health center where others do not enter during the meeting. In Rwanda, the floor is not a culturally acceptable space for physical examinations (even on a comfortable mat), so a portable cot about 2.5 feet above the floor was used for the semiprivate individual assessment completed for each woman during the group visit; this cot was shielded from the group space by a rolling privacy screen.

We convened 3 test groups among 22 pregnant and newly postnatal volunteers before implementation, with a focus on acceptability. During these mock visits, which did not include health assessments or health decision making, women were not shy about sharing deeply personal information within the safety of the group and were enthusiastic about all aspects of the model. These test activities did not result in significant changes to the model described by the technical working group, but minor adjustments to the discussion prompts and learning aids (for example, color instead of grayscale picture cards, with illustrations representing familiar clothing and settings) were made in direct response to women's feedback. Women in these test groups were asked to name the Rwanda group ANC and PNC model, and they named it *Ibaruke Neza Mubyeyi*, a very polite phrase in Kinyarwanda that translates as “May all of us mothers have safe pregnancies, births, and new motherhood.”

Two hundred seventeen CHWs and 54 health care providers were trained as new group ANC and PNC facilitators; 16% of the health care providers trained were midwives, and 84% of health care providers trained were nurses. As they planned and delivered each group visit, these facilitators referred to a Kinyarwanda‐language *Ibaruke Neza Mubyeyi* manual (see Supporting Information: Appendix S2). The English‐language version of this manual is also available in the supporting material of this article (see Supporting Information: Appendix S3).

## DISCUSSION

Recognizing the need to address the unacceptable neonatal mortality rate, the government of Rwanda and partners are conducting this cluster randomized controlled trial with the objective of assessing the impact of group ANC and PNC on gestational age at birth and other perinatal outcomes. Rwandan stakeholders collaborated to create a group ANC and PNC model with the hope that increased knowledge about perinatal complications among all group care participants will result in improved maternal and newborn health. This model, and this trial, were designed with the Rwandan health system's realities and priorities guiding every design and implementation decision.

Our experience suggests that the group process fundamental to successful group ANC and group PNC can successfully be employed to customize and implement this innovative health services delivery model in a new context at each step in the process. Employing this group process resulted in compromises by all members with an associated increase in trust and cohesion among them. The technical working group predicted that perceived risks to privacy would be a significant apprehension of women and families invited to participate in group care, and whether this cultural concern is a barrier to group care participation or inhibits active group discussion will be evaluated in this study. Creating simple learning activities for the Rwanda group ANC manual was also accomplished by the technical working group, and whereas activities using picture cards or other concrete manipulatives seem to be preferred by stakeholders and health care providers in this context, accumulating anecdotes suggest that other kinds of activities, such as story listening and telling and creating wishes for themselves and other group members, may be the types of activities preferred by participants.

Further iteration of the group care model in Rwanda is expected. We will collect anecdotal feedback from all study staff, including Master Trainers, throughout the trial. Qualitative research activities among ANC and PNC clients and health care providers are planned for 9 and 18 months after implementation. After each round of qualitative research, the technical working group will reconvene, using its successful group process, to reconsider the *Ibaruke Neza Mubyeyi* model and plan for possible implementation at a larger scale in Rwanda.

## CONFLICT OF INTEREST

The authors have no conflicts of interest to disclose.

## Supporting information


**Appendix S1**. Preterm Birth Initiative‐Rwanda cluster randomized controlled trial group antenatal and postnatal care model fidelity assessment data collection tool.

**Appendix S2**. Ibaruke Neza Mubyeyi: Group Antenatal and Postnatal Care in Rwanda [model manual, Kinyarwanda language]. San Francisco, CA: University of California, San Francisco, and East Africa Preterm Birth Initiative, 2017.

**Appendix S3**. *Ibaruke Neza Mubyeyi*: Group Antenatal and Postnatal Care in Rwanda [model manual, English translation]. San Francisco, CA: University of California, San Francisco, and East Africa Preterm Birth Initiative, 2017.
