# Supplementary material for: Use of a Facilitated Group Process to Design and Implement a Group Antenatal and Postnatal Care Program in Rwanda
Source: J Midwifery Womens Health. 2018 Sep 25;63(5):593–601. doi: 10.1111/jmwh.12871 (PMC6220997; doi:10.1111/jmwh.12871)
Supplement: Supplementary file 1 — Appendix S1. Preterm Birth Initiative‐Rwanda cluster randomized controlled trial group antenatal and postnatal care model fidelity assessment data collection tool. [file JMWH-63-593-s001.docx]

**Supporting Information Appendix S1**. Preterm Birth Initiative-Rwanda cluster randomized controlled trial group antenatal and postnatal care model fidelity assessment data collection tool

| Item # | Question | Answer/score |
| --- | --- | --- |
| 1 | Date of observation |  |
| 2 | Site of observation |  |
| 3 | Observer |  |
| 4 | How many facilitators were present during the group care visit? |  |
| 5 | Study identification codes of facilitators present |  |
| **For the following questions, please indicate your best assessment of how the co-facilitators during the group care visit today performed each skill or task.** Choose one answer per question:  0 = Not able to perform this skill even though the opportunity was present  1 = Made attempts but need significant help from the Master Trainer  2 = Have beginning skills but require more modelling, role-playing, and instruction  3 = Received a few minor suggestions from the Master Trainer  4 = Were fully competent | | |
| 6 | Prepared the group care room environment, including assessment equipment, learning materials, participant refreshment, and indicated medications |  |
| 7 | Communicated using language well understood by all participants, and responded appropriately to verbal and non-verbal cues |  |
| 8 | Performed assessments correctly and followed up on abnormal findings |  |
| 9 | Encouraged active participation in group activities/discussions and payed particular attention to participants who presented as reserved |  |
| 10 | Asked open-ended questions to promote discussion |  |
| 11 | Engaged next of kin and husbands in activities and discussions (if they were present) |  |
| 12 | Reinforced individual and group accomplishments |  |
| 13 | Participants spoke more than the co-facilitators spoke |  |
| 14 | Demonstrated mastery (accurate knowledge) of the curriculum, including discussion topics and key messages |  |
| 15 | Followed the lead of the women and could flexibly adjust the visit agenda to better meet women's needs and interests |  |
| 16 | Kept time |  |
| 17 | Provided antenatal and postnatal care screening, medications and referrals as indicated, consistent with the Rwanda Focused antenatal care and postnatal care packages |  |
