# Supplementary material for: Use of a Facilitated Group Process to Design and Implement a Group Antenatal and Postnatal Care Program in Rwanda
Source: J Midwifery Womens Health. 2018 Sep 25;63(5):593–601. doi: 10.1111/jmwh.12871 (PMC6220997; doi:10.1111/jmwh.12871)
Supplement: Supplementary file 2 — Appendix S2. Ibaruke Neza Mubyeyi: Group Antenatal and Postnatal Care in Rwanda [model manual, Kinyarwanda language]. San Francisco, CA: University of California, San Francisco, and East Africa Preterm Birth Initiative, 2017. [file JMWH-63-593-s002.pdf]

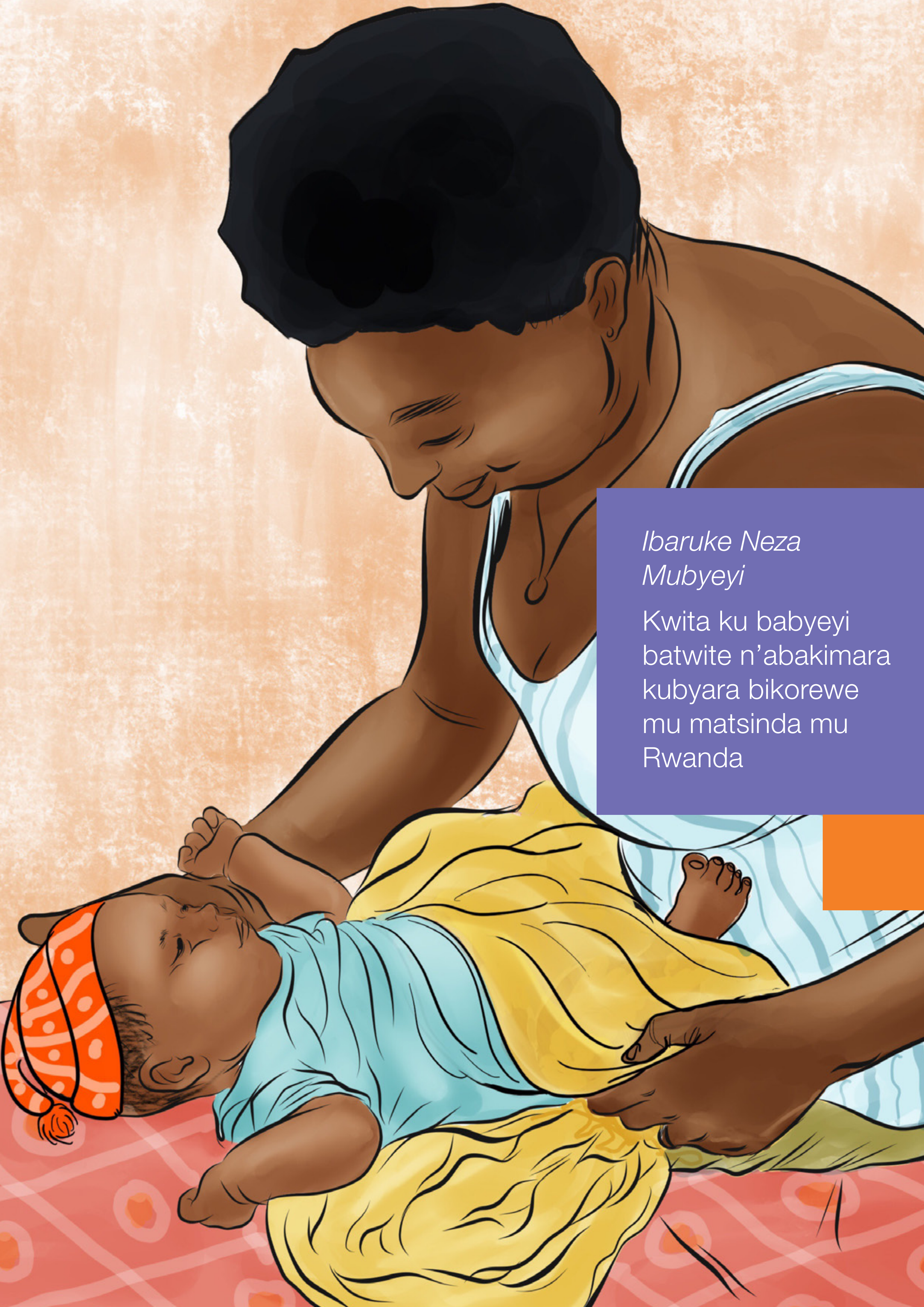

Ibaruke Neza Mubyeyi

Kwita ku babyeyi batwite n'abakimara kubyara bikorewe mu matsinda mu Rwanda

Ibaruke Neza Mubyeyi

Kwita ku babyeyi batwite n’abakimara kubyara bikorewe mu matsinda mu Rwanda

Uburenganzira bw'umuhanzi © 2017 Kaminuza ya California, San Francisco; Kaminuza y'u Rwanda n'Ikigo gishinzwe Ubuzima mu Rwanda (RBC). Uburenganzira bwose ni ubw'umuhanzi.

Gahunda yo kugabanya imfu z'abana bavuka batagejeje igihe muri Afurika y'Iburasirazuba

UCSF Global Health Sciences 550 16th Street, 3rd Floor,

Box 1224 San Francisco, CA 94158

Igitabo cyandikiwe muri Leta Zunze Ubumwe z'Amerika. Icapisha rya mbere, Nyakanga 2017.

Iki gitabo ni inyandiko ishobora kugerwaho na buri wese, igakwirakwizwa hakurikijwe uruhushya rwo gukwirakwiza ku buntu inyandiko zitagenewe gucuruzwa (Creative Commons Attribution-Noncommercial License), ruha abantu uburenganzira bwo kuzikoresha, kuzikwirakwiza no kuzitubura mu buryo butagamije ubucuruzi hifash-ishijwe igikoresho icyo ari cyo cyose, ariko ba nyirazo n'inkomoko yazo bikagaragazwa.

Gushimira

Iki gitabo cyanditswe ku bufatanye na Minisiteri y'Ubuzima mu Rwanda, Ikigo gishinzwe Ubuzima mu Rwanda, Kaminuza y'u Rwanda na Kaminuza ya California, San Francisco (UCSF) muri gahunda yo kugabanya imfu z'abana bavuka batagejeje igihe muri Afurika y'Iburasirazuba. Gahunda yo kugabanya imfu z'abana bavuka batagejeje igihe muri Afurika y'Iburasirazuba iterwa inkunga n'umuryango "Bill & Melinda Gates Foundation".

Imirimo yo gukusanya no kwegeranya ibikubiye muri aki gitabo mfashanyigisho yayobowe n'itsinda ry'abahanga n'abashakashatsi bakorera mu ishami ryita ku babyeyi batwite n'abakimara kubyara mu Rwanda (GANC/GPNC), aribo aba bakurikira:

Dushimimana Evodia
Gakwerere Mathias
Gatsinzi Patrick
Manzi Emmanuel
Mugeni Catherine
Musange Sabine
Mwali Assumpta
Nsaba Yvonne Delphine
Nshizirungu Jean Jacques
Sayinzoga Félix
Umuziga Providence

Itsinda rishinzwe ibya tekiniki rirashima akazi ku bijyanye no kwita ku babyeyi mu matsinda mbere yo kubyara kakozwe na Sharon Rising na bagenzi be nk'uko bigaragara muri iki gitabo.

Ibirimo

Intangiriro 1

Amashusho yagenewe gushyirwa ku makarita yifashishwa murigahunda *"Ibaruke Neza Mubyeyi"* 3

Imyiteguro yo kubonana n'ababyeyi mu itsinda 6

1. Gutegura no gusesengura amafishi y'abagize itsinda 6
2. Kwegeranya ibiri bukenerwe ku munsi wo gusura itsinda 6
3. Gutegura aho itsinda riri bukorere 7
4. Ibibanza kuganirwaho n'abayobora ibiganiro bombi 8

Uko izusuma ry'ababyeyi rikorwa 10

Uruhare rw'umujyanama w'ubuzima 10

Uruhare rw'umuforomo/umubyaza 10

Kwita ku babyeyi batwite no kubaha inama mu itsinda ku nshuro ya kabiri 13

Igikorwa cya 1: Gusobanura gahunda yo kwita ku babyeyi batwite no kubaha inama mu itsinda 13

Igikorwa cya 2: Ni ikihe kintu kiboneye cyo kurya no kunywa igihe umubyeyi atwite? (Imirire) 14

Igikorwa cya 3: Kwirinda no gukumira uburwayi 15

Igikorwa cya 4: Ibimenyetso mpuruza ku mugore utwite 16

Umusozo: Kurushaho kumenyana no kunga ubumwe 16

Kwita ku babyeyi batwite no kubaha inama ku nshuro ya gatatu 19

Gutangira gusuzuma ababyeyi bakihagera 19

Ikiganiro kibanza 19

Igikorwa cya 1: Ubuzima bwo mu mutwe 20

Igikorwa cya 2: Imyiteguro yo kubyara n'ibise 21

Igikorwa cya 3: Koboneza urubyaro 22

Igikorwa cya 4: Ibimenyetso mpuruza ku mugore utwite 23

Igikorwa gisoza: Kumenyana n'abandi 23

Kwita ku babyeyi batwite no kubaha inama mu itsinda ku nshuro ya kane 26

Isuzuma ry'ibanze ku babyeyi 26

Gutangiza ibiganiro 26

Igikorwa cya 1: Kujya ku nda no kubyara (n'ibimenyetso mpuruza) 27

Igikorwa cya 2: Kwita ku ruhinja n'ibimenyetso mpuruza 27

Igikorwa cya 3: Konsa 29

Igikorwa cya 4: Kwita ku mubyeyi nyuma yo kubyara n'ibimenyetso mpuruza ku mubyeyi 30

Igikorwa gisoza: Ubusabane no kumenyana 31

Kwita ku babyeyi no kubagira inama mu itsinda nyuma yo kubyara 33

Amasuzuma y'ibanze ku babyeyi 33

Ikiganiro kibanza 34

Igikorwa cya 1: Konsa 34

Igikorwa cya 2: Umuryango ufite ubuzima bwiza 35

Igikorwa cya 3: Ibimenyetso mpuruza ku ruhinja 36

Ikiganiro cyo gusoza: Ibyo ababyeyi bifuza kuzageraho 37

Intangiriro

Iki gitabo kigenewe abaforomo, ababyaza n'abajyanama b'ubuzima bazafatanya n'abashinzwe kwita ku babyeyi mu matsinda mbere na nyuma yo kubyara mu gihe cy'igeragezwa rizakorwa mu 2017–2019. Iki gitabo cyateguwe mu rurimi rw'Icyongereza nyuma gishyirwa mu rurimi rw'Ikinyarwanda, kikaba gikubiyemo amabwiriza yerekeranye no kwita ku babyeyi mu matsinda mbere na nyuma yo kubyara mu Rwanda, nko kwereka abantu ubu buryo bwo kwita ku babyeyi, gahunda zo gusura abantu, n'ibikorwa bigamije kubaka imibanire myiza mu itsinda.

Inyito y'iki gitabo, Ibaruke Neza Mubyeyi, yatoranyijwe n'abagore batwite bo mu Rwanda bagize amahirwe yo kwitabwaho binyuze muri gahunda yo kubitaho mu matsinda. Iki gitabo kigizwe n'amashusho anyuranye yakozwe n'umuhanzi w'Umunyarwanda Dolph Banza, wakoze amashusho agenewe gushyirwa ku makarita abagore bifashisha bigira mu matsinda yabo mbere na nyuma yo kubyara.

Iki gitabo kirareba ishyirwamubikorwa rya mbere rya gahunda yo kwita ku babyeyi mu matsinda mbere na nyuma yo kubyara mu Rwanda kandi gishobora kugenda gihindurwa n'abafatanyabikorwa mu macapisha azakurikira.

Iyi nyandiko ntigaragaza ibikurikizwa mu bushakashatsi, ibyakoreshejwe mu ikusanyamakuru, cyangwa ibijyanye no gushyira mu bikorwa no gukurikirana iyi gahunda yo kwita ku babyeyi mu matsinda mbere na nyuma yo kubyara mu Rwanda. Andi makuru ashobora kuboneka kuri ptbieastafrica@ucsf.edu.

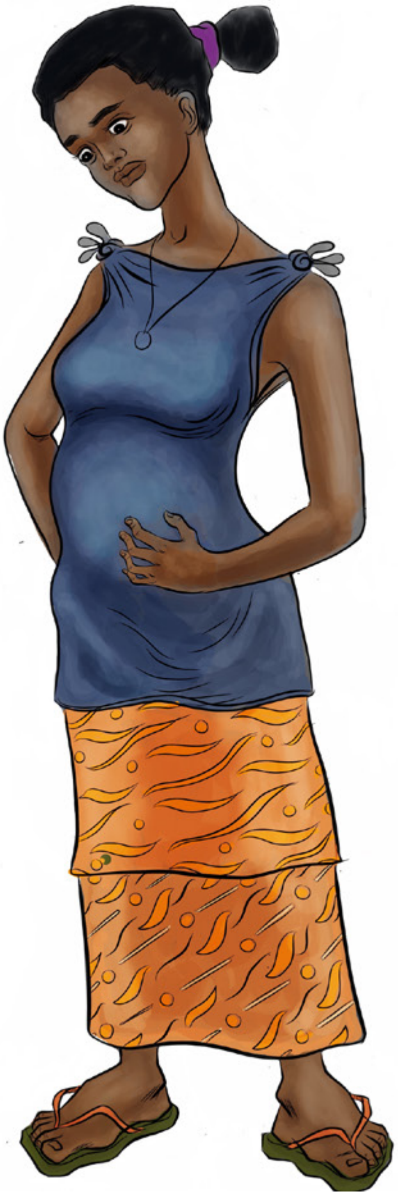

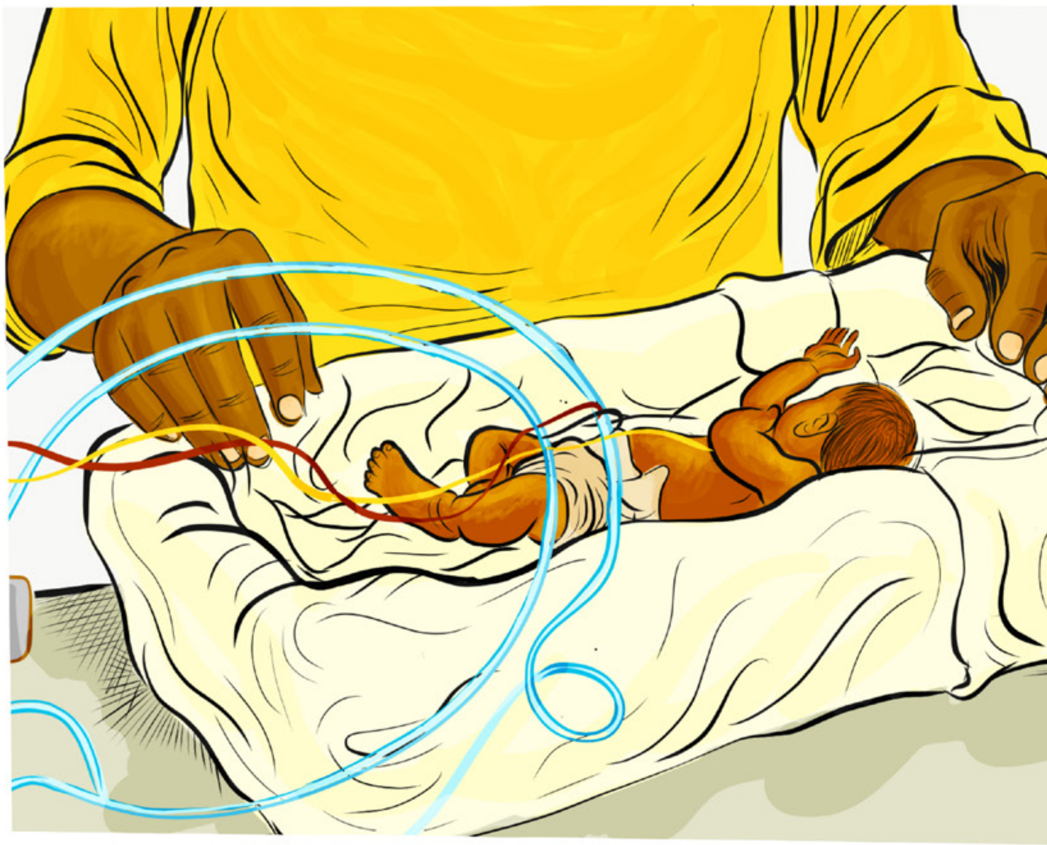

Kubyara inda itarageza ibyumweru 37

Amashusho yatunganijwe muri
gahunda ya Ibaruke Neza Mubyeyi

Amashusho yagenewe gushyirwa ku makarita yifashishwa muri gahunda "*Ibaruke Neza Mubyeyi*"

Itsinda rya tekiniki rishinzwe gahunda yo kwita ku babyeyi mu matsinda mbere na nyuma yo kubyara mu Rwanda ryasobanuye iby'ingenzi muri gahunda Ibaruke Neza Mubyeyi mu 2017. Itsinda ryakoze ibishoboka byose mu kubungabunga iby'ibanze muri ubu buryo bushya bwo kwita ku babyeyi, rigerageza kugaragaza serivisi zikubiye muri gahunda yo kwita ku babyeyi mu matsinda mbere na nyuma yo kubyara n'ibiranga urwego rw'ubuzima mu Rwanda.

1. **Abagore bashyirwa mu matsinda ahoraho buri tsinda rigizwe n'abantu bari hagati y'umunani na cumi na babiri (8-12), bazabyara mu gihe kimwe (bishobotse abafite amatariki yo kubyara yegeranye cyane).**

- Bishobotse hashyirwaho amatsinda yihariye ku bayeyi batwite ari abangavu.
- Ababyeyi bashishikarizwa kujya bubahiriza gahunda y'itsinda ryabo. Igihe hari uwagize ikibazo gituma asiba gahunda y'itsinda rye, ashobora kuza muri gahunda y'itsinda rikurikiraho kugira ngo adasigara inyuma mu gukurikiranwa.

2. **Ku nshuro ya mbere umubyeyi utwite yitabwaho nk'uko bisanzwe bigenda muri gahunda yo kwipimisha ku babyeyi batwite. Umubyeyi abonana n'umuforomo ku giti cye akitabwaho nk'uko biteganyijwe mu mabwiriza ngenderwaho agena uko umubyeyi utwite yitabwaho mu Rwanda. Buri mubyeyi akorerwa ibi bikurikira:**

- Kwiyandikisha;
- Gutegura idosiye yo gukurikirana umubyeyi utwite;
- Gukusanya amakuru arebana n'uko umubyeyi yagiye yivuza, arebana n'imyanya myibarukiro kimwe n'arebana n'uko yagiye abyara, ku mubyeyi utwite inshuro zirenze imwe;
- Gusuzuma umubiri w'umubyeyi mu buryo bwimbitse;
- Kumenya neza umunsi umubyeyi ashobora kuzabyariraho n'umunsi aherukira kujya mu mihango;
- Gufata no gupima ibizamini by'inkari hagamijwe kureba ko nta ndwara z'urwungano rw'inkari afite;
- Kuri iyi ngingo, hagomba gukorwa ibi bikurikira;
- Gukora ibizamini by'amaraso.
    - » Kureba ko nta kibazo cy'amaraso make umubyeyi afite (Hb);
    - » Ubwoko bw'amaraso n'igice aherereyemo (Blood type and Rhesus);
    - » Gusuzuma ko nta mburugu (RPR) na Virusi itera ubwandu bwa SIDA (no gupima umugabo we).
- Ibigomba kwitonderwa mu buzima bwa buri munsi: Mu buzima bwa buri munsi, hagomba kwitonderwa ibi bikurikira:
    - » Ubuvuzi;
    - » Imiti;
    - » Inkingo.
- Koherezwa kubonana n'umuganga w'inzobere/ igihe bibaye ngombwa;
- Ubutumwa n'inyigisho ku bijyanye n'ubuzima bw'umubyeyi n'umwana
- Gusobanura uko gahunda yo gukurikiranwa mu matsinda iteye (IBARUKE NEZA MUBYEYI) no gushyira abagore mu matsinda yabo (Icyiciro cya kabiri cyo guhabwa ubujyanama ku babyeyi bamaze ibyumweru 20 kugeza kuri 24 basamye).
    - » Ababyeyi bose bagenewe ubujyanama mu by'ubuzima mu gihe batwite bashyirwa mu matsinda;
    - » Ababyeyi bashyirwa hamwe mu matsinda hakurikijwe iminsi y'agateganyo yo kuzabyariraho mu kwezi kumwe cyangwa se iminsi yenda kwegerana;
        - › Ingengabihe igaragaza uko itsinda rizajya riza ku kigo nderabuzima itegurwa kare igashyirwa ku ndangaminsi;
        - › Buri mubyeyi utwite amenyeshwa amatariki abagize itsinda rye bazahurira mu gihe cyose cyo gutwita ndetse na nyuma yo kubyara;
    - » Abagore baza guhabwa ubujyanama bwa mbere baratinze, basabwa kubuhabwa bakurikije inshuro zisigaye mu itsinda bashyizwemo kandi bagasabwa no kwitabira guhabwa ubujyanama inshuro bacikanywe (urugero kwitabira guhabwa ubujyanama mu itsinda ubwa 2 n'ubwa 3 mu yandi matsinda);
    - » Abagore basabwa kwemera ku bushake bwabo kwinjira muri gahunda no gutanga uburenganzira ko amakuru ku buzima bwabo azesengurwa;

» Abagore batigeze bahabwa ubujyanama na rimwe mu gihe bari batwite ariko bakabyarira kwa muganga mbere yo gusezererwa ngo batahe, basabwa kwinjira mu itsinda ritanga ubujyanama nyuma yo kubyara bakazaza muri gahunda y'itsinda nyuma y'ibyumweru bitandatu.

3. **Gukurikirana ababyeyi batwite ku nshuro ya 2 kugeza ku ya 4 ndetse na nyuma y'ibyumweru 6 babyaye bizajya bikorwa mu matsinda ku buryo bukurikira:**

Ubujyanama	Igihe umubyeyi agezemo
Ubujyanama ku babyeyi batwite bukorerwa mu itsinda ku nshuro ya 2	Inda y'ibyumweru 20–24
Ubujyanama ku babyeyi batwite bukorerwa mu itsinda ku nshuro ya 3	Inda y'ibyumweru 28–32
Ubujyanama ku babyeyi batwite bukorerwa mu itsinda ku nshuro ya 4	Inda y'ibyumweru 36–40
Ubujyanama ku babyeyi igihe gito babyaye.	Ibyumweru 6 nyuma yo kubyara

4. **Ababyeyi bagize itsinda bicara ku ruziga ahantu abandi bakozi cyangwa abarwayi batinjira mu gihe cy'ubujyanama;**

5. **Buri tsinda riyoborwa n'abantu babiri: Umukozi umwe ushinzwe ubuzima bw'umubyeyi (umubyaza/umuforomo) afatanije n'umujyanama w'ubuzima;**

6. **Kugira ibanga no kubahana bigirwa ihame mu babyeyi batwite n'ababafasha;**

7. **Abo babyeyi bahabwa amazi meza yo kunywa hakoreshejwe udukoresho twiza twa palastike kandi dufite isuku;**

8. **Gusuzuma uko ubuzima bw'umubyeyi buhagaze bikorwa mu minota 30 ibanza iyo abagore bahagereye igihe cyari giteganijwe.**

9. **Ababyeyi bashishikarizwa kugira uruhare runini mu isuzuma ry'ubuzima ribakorerwa (urugero: gupima umuvuduko w'amaraso n'ibiro);**

10. **Isuzuma ricirirtse rikorerwa ababyeyi ahasa n'ahiherereye mu mwanya itsinda ryateraniyemo;**

- Ababyeyi bose bari mu itsinda bagomba gusuzumwa mu minota 30 ibanza.

11. **Ababyeyi n'abana bahabwa ubuvuzi no kwitabwaho bisanzwe biteganijwe mu gihe cyo gutwita ndetse na nyuma yo kubyara (ANC/PNC), ndetse n'ubuvuzi bwihariye nko ku babyeyi babana na virusi ya SIDA (Gahunda yo kurinda umubyeyi kwanduza umwana);**

- Ubundi buvuzi bwose butari rusange butangwa mu ibanga mu rwego rwo kwirinda ko hagira ababyeyi bahabwa akato.

12. **Ababyeyi bafite ibibazo bisaba ubufasha bwihariye butaboneka ku kigonderabuzima boherezwa ku bitaro by'akarere n'umukozi ushinzwe ubuzima bw'umubyeyi nkuko biteganijwe mu mabwiriza ya Minisiteri y';**

13. **Inama z'ingenzi zijyanye no kwita ku babyeyi batwite n'abamaze igihe gito babyaye zitangirwa mu kiganiro cy'itsinda kiyobowe n'umuforomo/umubyaza afatanije n'umujyanama w'ubuzima ariko ntibiharire ijambo ahubwo bagaha ababyeyi urubuga n'umwanya uhagije wo gutanga ibitekerezo;**

14. **Inyigisho zitangwa hakurikijwe amahame yo kwigisha abantu bakuru akurikira:**

- Gusubiramo insanganyamatsiko;
- Kungurana ibitekerezo nk'urungano;
- Kugira uruhare mu byigwa hakoresheje ibyiyumviro binyuranye by'umubiri;
- Gukemura ibibazo binyuze mu biganiro;
- Kwisanzura mu biganiro no gukora imyitozo ngorora mubiri.

15. **Kungurana ibitekerezo mu itsinda bimara isaha imwe;**

16. **Abagize buri tsinda ry'ababyeyi bihitiramo niba bashaka gutumira abagabo babo cyangwa abandi bafitanye isano rya bugufi muri gahunda y'itsinda;**

17. **Abafatanyije mu kuyobora ibiganiro « batanga inama » buri gihe uko itsinda rirangije ibiganiro, mu rwego rwo gukomeza kungurana ubumenyi no kunononsora ireme ry'isuzuma.**

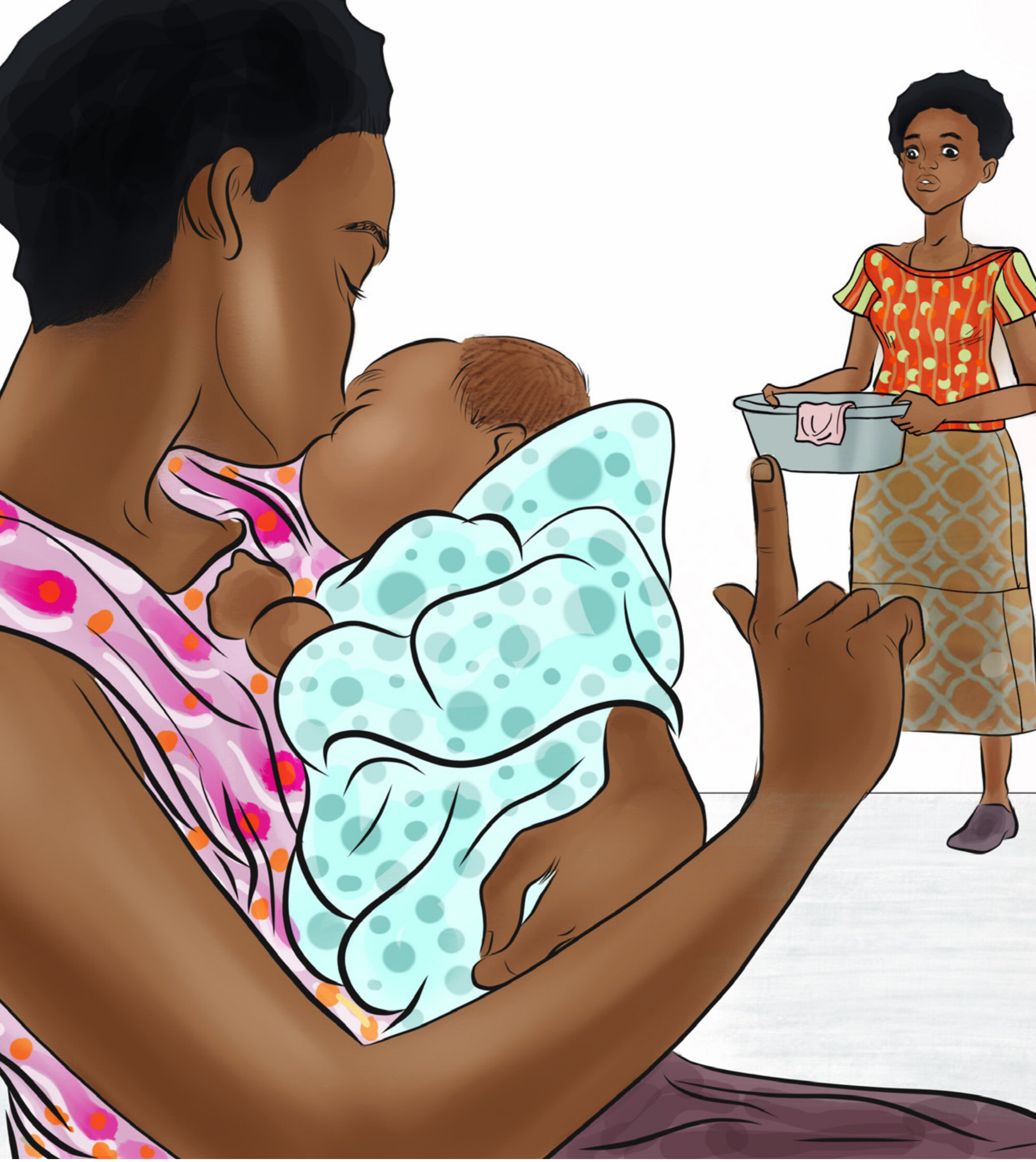

Kuba uretse gukarabya uruhinja, ukarukarabya bwa mbere nibura nyuma y’amasaha 24 ruvutse

Amashusho yatunganijwe muri gahunda ya Ibaruke Neza Mubyeyi

Imyiteguro yo kubonana n’ababyeyi mu itsinda

Ku minsi yo gutanga ubujyanama ku babyeyi bibumbiye mu itsinda, ugomba kwita kuri ibi bikurikira mu rwego rwo gukora isesengura ryimbitse, kunoza gahunda yo kwita kuri buri mubyeyi no gutegurana ubushishozi uburyo bwo gusura itsinda ry’ababyeyi. Iyi myiteguro ishobora kugutwara iminota 30 kugeza kuri 60.

1. Gutegura no gusesengura amafishi y’abagize itsinda

Idosiye ya buri mubyeyi washyizwe mu itsinda ishyirwa mu gakoresho kabika inyandiko k’udufashi dutatu. Iyo dosiye yitwa « ifishi y’umuzima bw’umubyeyi » kandi itangwa bwa mbere igihe umugore yipimisha inshuro ya mbere. Amafishi y’ubuzima bw’ababyeyi bibumbiye mu itsinda rimwe, ashyirwa hamwe akandikwaho izina ry’itsinda rigaragaza ukwezi n’umwaka ababyeyi bashobora kuzabyariraho na nimero z’itsinda. Urugero, itsinda rishobora kwitwa « Gicurasi 2017 & 2 » kuko ababyeyi barigize bazabyara mu kwezi kwa Gatanu 2017 bakaba bari mu itsinda rya kabiri ryashyizweho ry’abazabyara muri uko kwezi. Ikindi ugomba kwitegura, ni ugushakisha amafishi y’ubuzima bw’ababyeyi bari mu itsinda riri busurwe.

Mbere y’uko ababyeyi bahagera, reba amafishi yabo. Kuri buri fishi, reba ahabugenewe wandikemo amakuru ajyanye n’isuzuma ry’uwo munsi. Andikamo itariki y’umunsi n’amezi inda y’uwo mugore ifite (koresha ikaramu y’igiti, bityo mu gihe umugore ataje ube wasiba itariki y’uwo munsi). Subiramo buri fishi witonze kugira ngo uteganye guha umugore ubufasha ari bukenere kuri wo munsi:

- Inda ye igize igihe kingana iki ukurikije agakoresho babariraho igihe cy’inda n’itariki babariraho inda nk’uko byanditse muri buri fishi ye.
  - » Yasuzumishijwe icyuma kireba uko umwana ameze mu nda? (Ekogarafi niba ihari)
  - » Aheruka mu mihango ryari niba abizi?
  - » Hari ikizamini yakorewe ubwo yisuzumishaga bwa mbere?
- Ibi bizamini byose bishoboka by’aho basuzumira ibizamini (laboratwari) byarakozwe, n’ibisubizo umubyeyi yarabimenye?
  - » Kumenya niba umugore afite amaraso ahagije (Hb);
  - » Kureba niba ataranduye mburugu na SIDA;
  - » Gupima ibizamini by’inkari (Proteinurie, Cullot, etc);
  - » Gupima ubwoko bw’amaraso na Rezisi (Rhesus);
  - » Gupima udukoko dutera indwara;
  - » Niba bitarakozwe, ufate ibyo bizamini cyangwa ukoreshe ibisubizo by’uwo munsi
- Yahawe urukingo rwa tetanosi rwa 1, akeneye urwa 2 cyangwa urwa 3 uwo munsi?
- Hari ubundi buvuzi bwihariye ahabwa ugomba gukurikirana uwo munsi?
- Hari ibibazo byihariye afite bisaba ko agira irindi suzuma akorerwa uwo munsi cyangwa yoherezwa ahandi?

2. Kwegeranya ibiri bukenerwe ku munsi wo gusura itsinda

Ku munsi wo gusura itsinda, ugomba gutegura udupaki tw’ibinini bya feri/aside folike byo guha ababyeyi bari bwitabire gahunda y’umunsi—agapaki kamwe k’ibinini bya feri kuri buri mugore uteganya ko aza. Ugomba no gutegura kandi ibinini by’inzoka (Mebendazole) ndetse ugashyira hafi n’inkingo za Tetanosi niba bishoboka.

Umuti	Urugero utangwamo
Mebendazole mg 500	Ikinini 1
Ibinini bya fer/ aside folike	Ibinini 30-60
Urukingo rwa Tetanosi	Gutera mu ruzigira rw’ukuboko urushinge rurimo ml 0.5

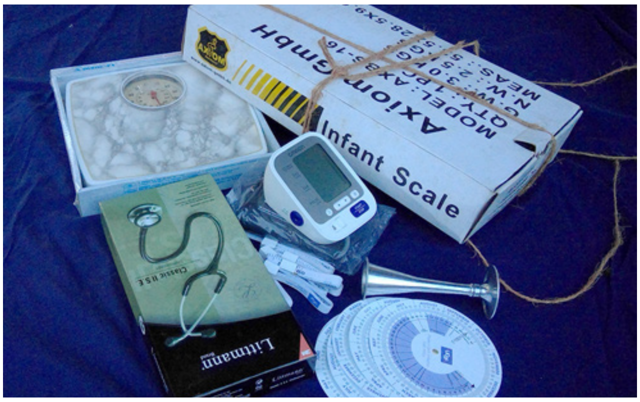

Tegura amazi meza Ababyeyi baza kunywa nibahagera; tereka ayo mazi ku meza n'udukombe twa palasitiki; egeranya kandi ibyo uza kwifashisha mu gusuzuma.

Umukozi w'ivuriro akenera:

- Umukozi w'ivuriro, asabwa gutegura ibikoresho bikurikira:
  - » Agakoresho kifashishwa mu kumva uko umutima w'umwana uri mu nda utera;
  - » Metero ikoreshwa mu gupima indeshyo ya nyababyeyi;
  - » Igipimo cy'ubushyuhe.

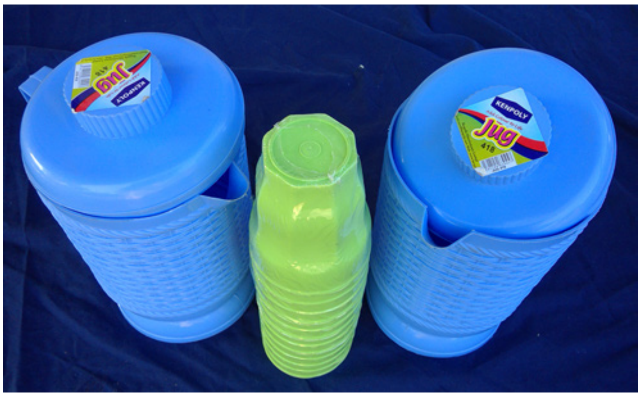

- Umujyanama w'ubuzima akenera:
  - » Umunzani;
  - » Agakoresho gapima umuvuduko w'amaraso;
  - » Agakoresho gapima umuzenguruko w'ikizigira cy'ukuboko (MUAC).

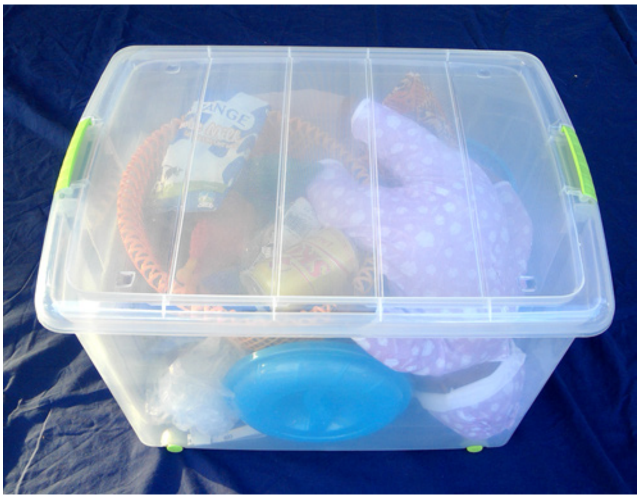

Ugomba kuzana imfashanyigisho uri bukenere uwo munsi mu gusutanga inama ku bagize itsinda. Egeranya imfashanyigisho zijyanye na gahunda y'umunsi. (Niba utabizi neza, reba mu ndangaminsi nurangiza urebe inshuro baba bajeho, hanyuma urebe muri iyi mfashanyigisho umenye imfashanyigisho zikenewe).

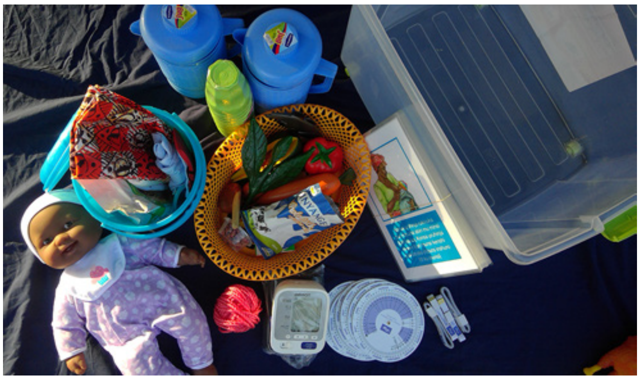

3. Gutegura aho itsinda riri bukorere

Mbere na mbere hitamo ahantu haboneye mu cyumba itsinda riza guhuriramo, unateganye ahandi hasa n'ahiherereye wasuzumira ababyeyi buri umwe umwe. Ubusanzwe ureba imfuruka yitaruye ugereranije n'aho umuryango n'idirishya biri. Shyirayo agatanda ko gusuzumiraho n'agatebe umubyeyi yicaraho asuzumwa biri ahiherereye. Iyegereze inkingo za tetanosi umuforomo/umubyaza ari buze gukoresha igihe bibaye ngombwa.

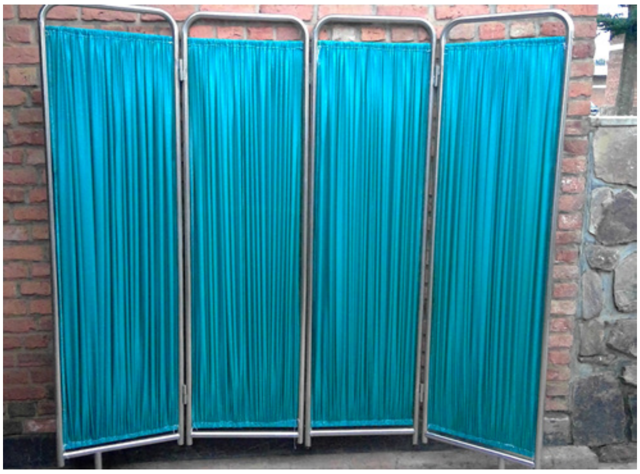

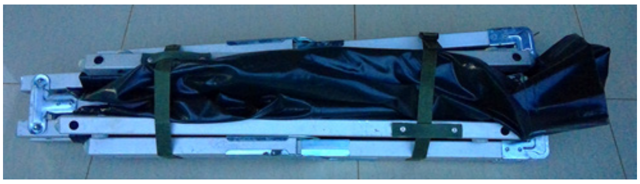

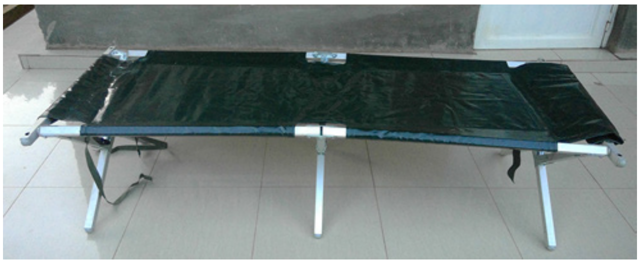

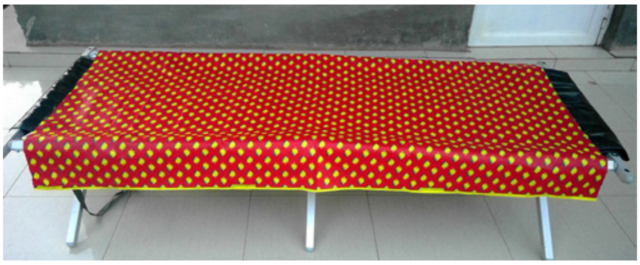

Uburyo icyumba ababyeyi bisuzumirashamo mu matsinda kiba giteye

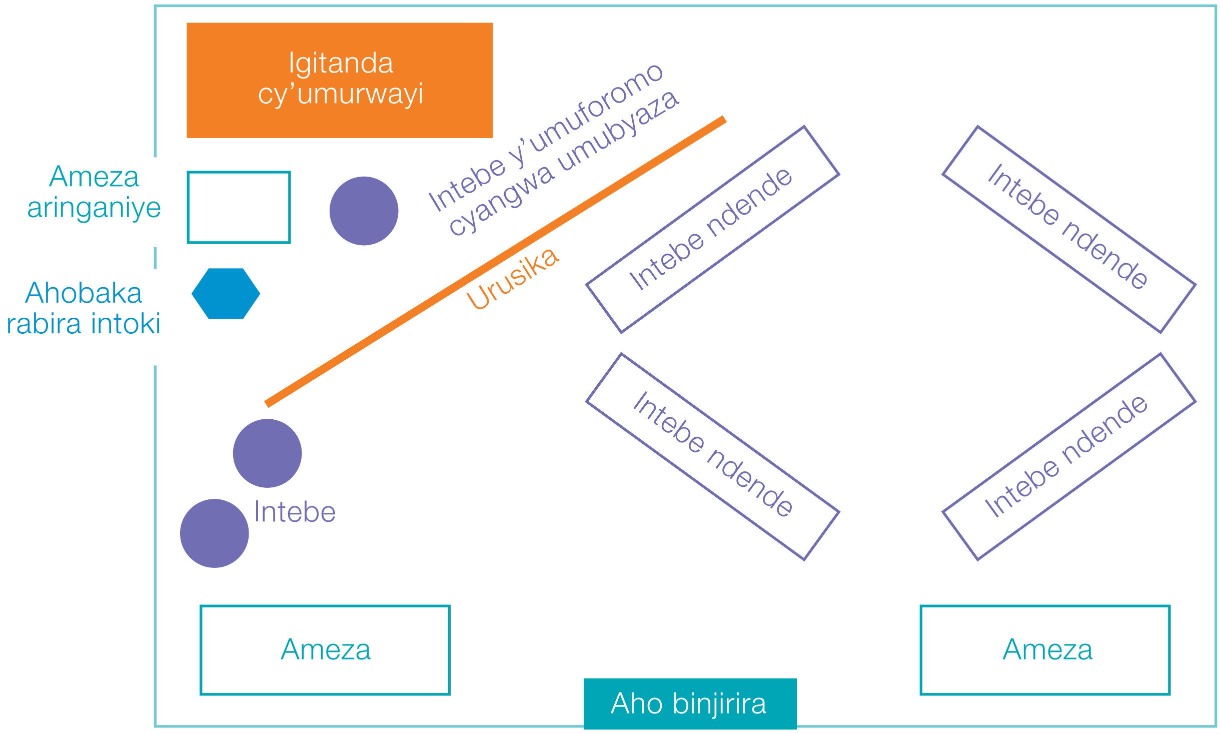

Noneho, tegura aho kwicara (intebe zitambitse) mu buryo bwa mpande enye cyangwa ubwa mpande eshatu mu cyumba, usiga umwanya uhagije w'inzira ngo ababyeyi binjiremo ariko nanone ugerageza kuzegeranya bihagije kugira ngo bize koroshya ikiganiro nyuma. Uburyo bwa mpande enye zingana buba bwiza cyane iyo ufite ababyeyi 11 cyangwa barengaho, noneho mpande eshatu ikaba nziza iyo hari buze abari munsi ya 11. Buri mubyeyi yagombye kuba yicaye iruhande rw'undi mubyeyi. Ntabwo hagombye kubamo intera nini hagati y'ababyeyi igihe bicaye. Niba ufite za ntebe zicaraho umuntu umwe, zitere ku ruziga.

Mu gusoza imyiteguro, shyiraho ameza n'intebe nk'imwe cyangwa ebyiri imbere hafi y'aho binjirira mu cyumba itsinda riri buteraniremo. Shyira ibikoresho bikurikira ku meza:

a. Akagakoresho gapima umuvuduko w'amaraso;
b. Ikaramu;
c. Udupaki tw'ibinini bya feri kuri buri muntu;
d. Agakombe k'ibinini by'inzoka/Mebendazole bya mg 500.

Shyira umunzani hasi hafi aho. Niba bishoboka, Tereka amazi kuri ayo meza n'udukombe twa palasitiki duhagije ku mubare w'ababyeyi bari buze.

4. Ibibanza kuganirwaho n'abayobora ibiganiro bombi

Abayobora ibiganiro bombi bahura iminota 5 kugira ngo bategure uko imigendekere y'ibiri bukorwe uwo munsi. Mu gihe cyo kubiganiraho abayobora ibiganiro bavugana ibiri bukorwe n'uko bikurikirana; bakanavugana uri buyobore buri gikorwa. Bagomba gusaranganya inshingano ku buryo bungana; mu gihe cyo gutanga ibiganiro, bombi bagenda bakuranwa mu bikorwa birimo inyigisho.

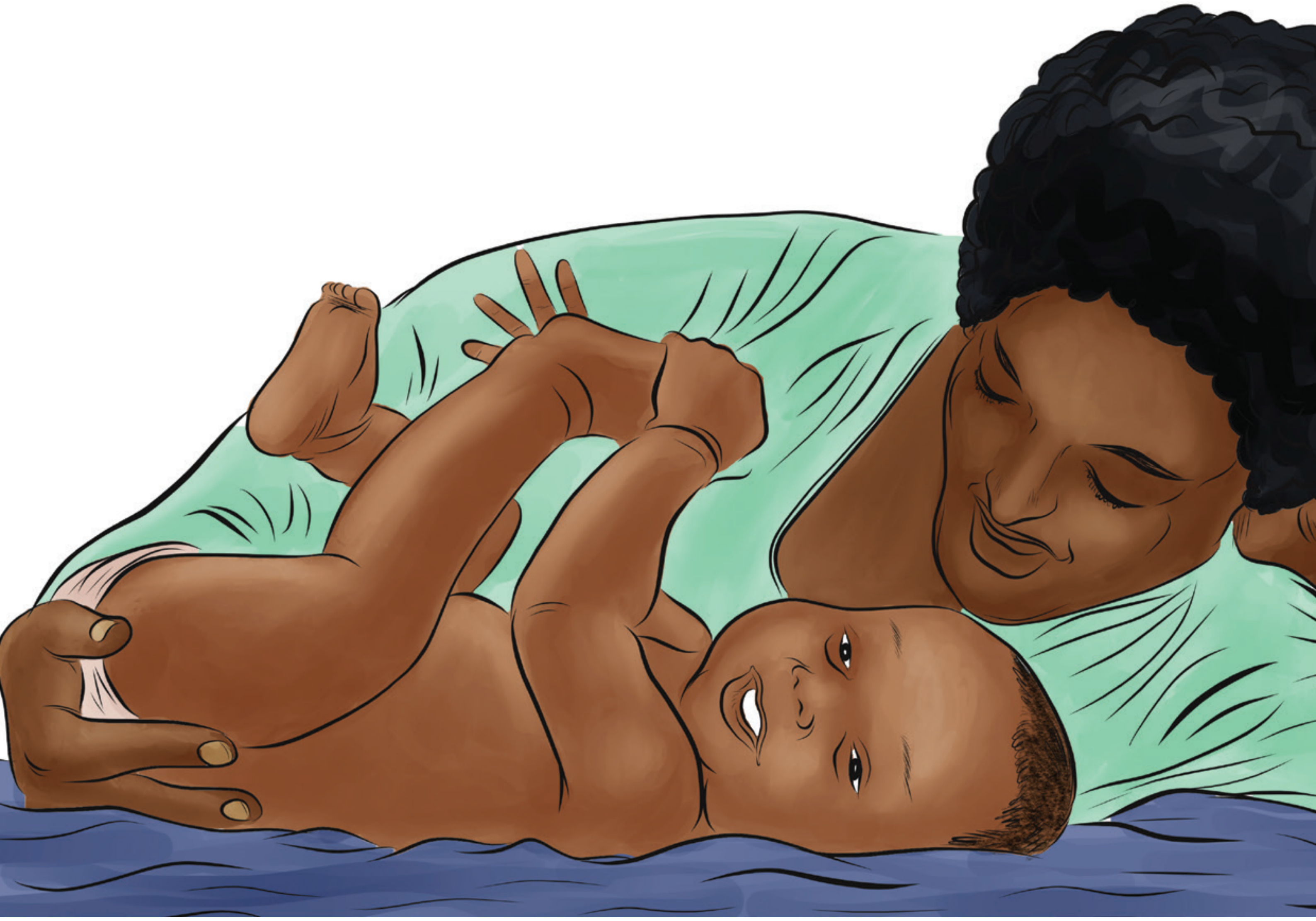

Gusekesha, kuririmbira, kuganiriza,
no gusekera uruhinja

Amashusho yatunganijwe muri
gahunda ya Ibaruke Neza Mubyeyi

Uko izusuma ry'ababyeyi rikorwa

Iyo ababyeyi bahageze ku isaha itsinda ryumvikanyeho, buri wese agira uruhare mu gikorwa cyo gufata ibipimo by'ingenzi by'ubuzima bw'umubyeyi utwite. Habanza umujyanama w'ubuzima hagakurikiraho umubyaza/umuforomo. Ababyeyi basobanurirwa birambuye ku byerekeye gusuzumwa mu itsinda igihe baje kwisuzumisha inda ku nshuro ya 2, bityo bakaba bakora ayo masuzuma igihe bahageze (kwisuzumisha inda ku nshuro ya 3 n'iya 4 no guhabwa inama n'ubufasha nyuma yo kubyara) batarinze gutegereza ngo buri wese mu bagize itsinda ahagere ngo babone gutangira.

Uruhare rw'umujyanama w'ubuzima

Umujyanama w'ubuzima ashinzwe gukurikirana:

a. Ibiro;

b. Umuvuduko w'amaraso;

c. Umuzenguruko w'ikizigira (MUAC);

d. Gutanga ibinini by'inzoka (Mebendazole ya mg 500);

e. Gutanga ibinini bya feri mu dupaki.

Umujyanama w'ubuzima yicara hafi y'ameza ari aho binjirira aho isuzuma mu itsinda riri bubere. Umujyanama w'ubuzima aha ikaze buri mubyeyi akagenzura ibikorerwa aho ngaho; akibutsa umubyeyi uko bakoresha umunzani, ibikoresho bipima umuvuduko w'amaraso n'ibipima umuzenguruko w'ukuboko, akabashishikariza kugira uruhare mu ifatwa ry'ibipimo. Itariki, ibiro, umuvuduko w'amaraso n'ibipimo by'umuzenguruko w'ukuboko (ku kizigira) bigomba kwandikwa ku ifishi umubyeyi akurikiranirwaho.

Umujyanama yibutsa umubyeyi kwandika itariki ku ikarita ahegeranye n'inimero y'inshuro y'iryo suzuma. Umubyeyi ashobora kwandika ibiro, umuvuguko w'amaraso n'ibipimo by'umuzenguruko w'ikizigira cy'ukuboko ahabugenewe ku ifishi, iburyo handitseho «Icyitonderwa/Icyongerwaho ».

Ababyeyi bamwe na bamwe ntibazi gusoma kandi ntibagomba kurobanurwa mu bandi cyangwa ngo bakozwe isoni. Umujyanama w'ubuzima cyangwa abandi bagore batwite bagomba kubafasha uko bashoboye kose kugira ngo bagire uruhare rungana mu bushobozi bafite.

Umujyanama w'ubuzima kandi ashishikariza buri mubyeyi kunywa ikinini cy'inzoka (Mebendazole) akanywa kandi amazi ahagije. Umujyanama w'ubuzima asobanuurira ababyeyi ko ari ngombwa kunywa icyo kinini kuko akamaro kacyo ari ukwica inzoka zose zaba ziri mu mubiri we, agire ubuzima bwiza.

Umujyanama w'ubuzima ashishikariza buri mubyeyi gufata agapaki k'ibinini bya feri kugira ngo ajye abifatira mu rugo; yibutsa abagize itsinda ko ababyeyi batwite bagomba gufata ibini bya feri buri munsi kandi ko ibi binini bigombwa kubikwa kure aho abana batagera kuko bishobora gutera ingaruka zikomeye umwana aramutse abinyoye ari byinshi.

Buri mubyeyi amara iminota nk'itatu muri iki gikorwa cyo gufata ibipimo. Nyuma y'aho Umujyanama w'ubuzima yibutsa buri mugore kugana ahateguwe agasuzumwa n'umuforomo cyangwa umubyaza. Mu gihe bategereje kugerwaho cyangwa barangije gusuzumwa n'umuforomo/umubyaza, ababyeyi bicara hamwe bateguriwe bakaganira ku birebana n'ubuzima bw'umugore utwite muri rusange.

Umuforomo/Umubyaza ahamagara umubyeyi umwe umwe akaza gusuzumwa. Umuforomo /umubyaza asaba umubyeyi kwicara ku gatanda basuzumiraho noneho agapima umugore nk'uko byateganyijwe.

Uruhare rw'umuforomo/umubyaza

Umubyeyi wese umaze gukorerwa isuzumwa n'umujyanama w'ubuzima, ahabwa amahirwe yo kubonana n'umuforomo/umubyaza kugira ngo amukorere irindi suzuma riciriritse.

Asaba umubyeyi kwicara ku gitanda cyo gusumiraho, akareba ku ikarita yisuzumishirijeho, akagenzura ibiro, umuvuguko w'amaraso n'ibipimo by'umuzenguruko w'ikizigira umugore afite ubundi akamubaza ku bimenyetso mpuruza bikurikira:

- « Waba ufite ibibazo bijyanye no guhumeka bigoranye, kuva, kuribwa mu nda bikomeye, ukunda gufatwa n'imbwa (Crampes), kubabara umutwe bikabije, kutabona neza, kuzana ibintu by'uruzi bidasanzwe mu gitsina cyangwa kutakaza amazi menshi, kubabara igihe wihagarika, kugira udusebe mu myanya ndangagitsina, kuzana ibintu binuka mu gitsina cyangwa ubundi uburwayi bukomeye? ».

Niba umubyeyi agusubije yego kuri kimwe muri ibi bibazo, asabwa gusigara kugira ngo asuzumwe neza itsinda risojwe. Umubyeyi aramutse agize ikibazo cy-ihutirwa, ushobora guhita umushakira uko asuzumwa ako kanya. Urugero:

- Uramutse ubonye hari ikintu cyihutirwa nko kuba yagira umuvuduko w'amaraso uri hejuru cyane, ushobora gusaba bagenzi bawe cyangwa se umuyobozi w'ikigo nderabuzima bagashaka uko baha uwo mubyeyi ubufasha bukenewe ku buryo bwihuse. Shaka uko yasuzumwa neza cyangwa se n'uko yakoherezwa ku bitaro.
- Igihe hari ikibazo kitihutirwa cyane, nko kuba agira uruzi mu gitsina runuka nabi, muhe gahunda yihariye yo gusuzumwa nyuma y'igikorwa cy'itsinda kandi bikorwe mu ibanga rikomeye ku buryo abagize itsinda bandi batamenya ikibazo umubyeyi afite.
- Igihe umubyeyi yaba akeneye ubundi bujyanama cyangwa kwitabwaho mu rwego rwo kumurinda kwanduza uwo atwite virusi itera SIDA, musabe kuza gusigara abandi batashye kugira ngo yitabweho byihariye.

Umuforomo/Umubyaza akurikirana kandi ibibazo byihariye byagaragaye ku babyeyi runaka. Niba umubyeyi avuze ko atameze neza, mupime umuriro ukoresheje igipimo cy'umuriro ako kanya. Umuforomo/umubyaza mu isuzuma kandi abaza umugore niba umwana akina mu nda neza. Dore ibyo ashobora kumubaza:

- « Inda ironka neza? ».

Niba umubyeyi avuze ko yumva inda itonka neza, kurikirana ikibazo, ukore isuzuma ryimbitse ni biba ngombwa wohereze umubyeyi ku bitaro.

Kuzuza inyandiko zose zijyanye n'ibyo wasuzumye mu ifishi y'ubuzima bw'umubyeyi ku itariki y'uwo munsi:

- Imiti ya ngombwa ku mubyeyi wese utwite
    - » Baza umubyeyi niba yarafashe ikinini kimwe cy'inzoka (Mebendazole) yahawe ahageze kandi ubyandike ku ifishi ye;
    - » Baza umubyeyi niba yarahawe ibinini bya feri na aside folike, byandike;
    - » Niba umubyeyi agomba guhabwa urukingo rwa tetanosi uwo munsi, musabe aze gusigara itsinda risoje kugira ngo akingirwe.
- Gusuzuma inda
    - » Iri suzuma rikubiyemo isuzuma ry'uburebure bwa nyababyeyi, n'uko umutima w'umwana utera mu nda.

Mu gusoza baza umubyeyi ibi bikurikira:

- « Hari ibindi bibazo cyangwa ibintu biguhangayikishije ». Niba ubona bikwiye, baza umugore niba ikibazo cye waza kugisubiriza mu kiganiro uza kugirana n'itsinda ryose kugira ngo abandi babyeyi babe bakumviraho mu gihe hari undi baba bahuje ikibazo.

Igikorwa cyo gusuzuma inda kuri buri mubyeyi gitwara iminota itarenze itatu. Buri suzuma ryangombye kuba rigufi kandi ryihariye kuri buri mubyeyi, ariko na none ntibibuze umukozi kwita ku mubyeyi no kumuha amakuru yaba mu mvugo no mu kuvugisha ibimenyetso by'umubiri, amwereka ko yishimiye kumubona yaje kwisuzumisha mu itsinda. Ushobora gukenera gukoresha isaha yabugenewe (Horloge) kuri buri suzuma kugira ngo wubahirize igihe.

Igikorwa kibanza cyo gufatwa ibipimo by'ingenzi no gusuzuma ababyeyi ntikigomba kurenza iminota 30 muri rusange iyo ababyeyi bose bubahirije igihe bahawe. Igikorwa cyo gusuzuma cyose kiramutse kirengeje iminota 30, isuzuma ry'itsinda ntiryagenda neza kuko ababyeyi bararambirwa kandi ntibaze kugira umwanya uhagije wo kugirana ibiganiro byubaka mu itsinda.

Igihe buri mubyeyi wese amaze gusuzumwa, ababyeyi bose n'abayoboye ikiganiro bicara hamwe ku ruziga. Abayobora ibiganiro bombi bagomba kwicara aho bateganye ku mpamvu ebyiri:

1. Ababyeyi n'abayobora ibiganiro baba « bagiye ku rwego rumwe» iyo bicaye ku ruziga;
2. Abayobora ibiganiro kandi bashobora guhana

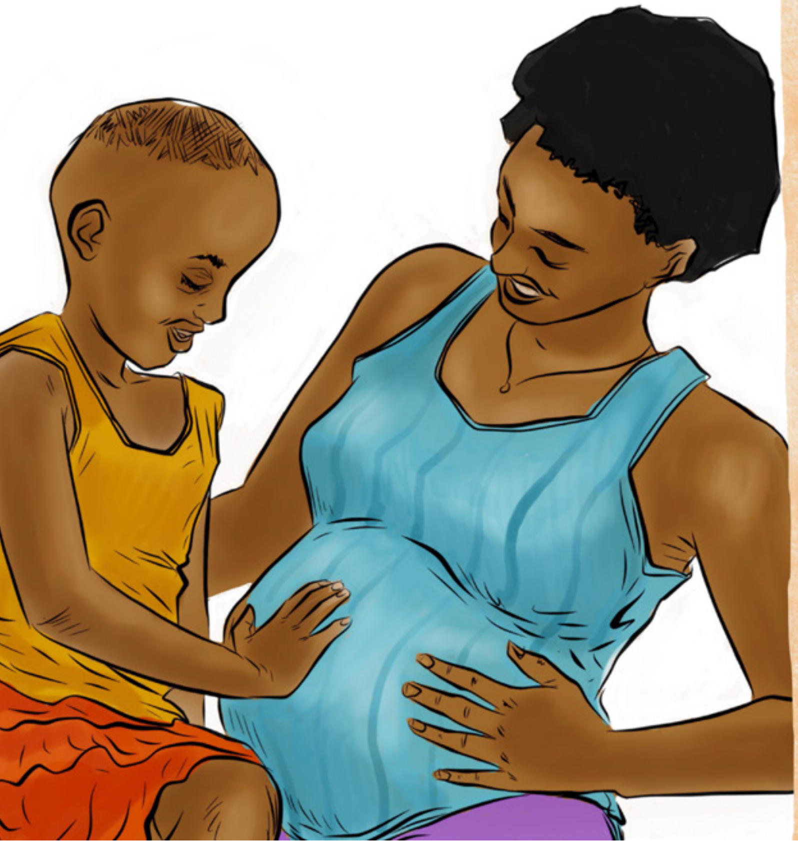

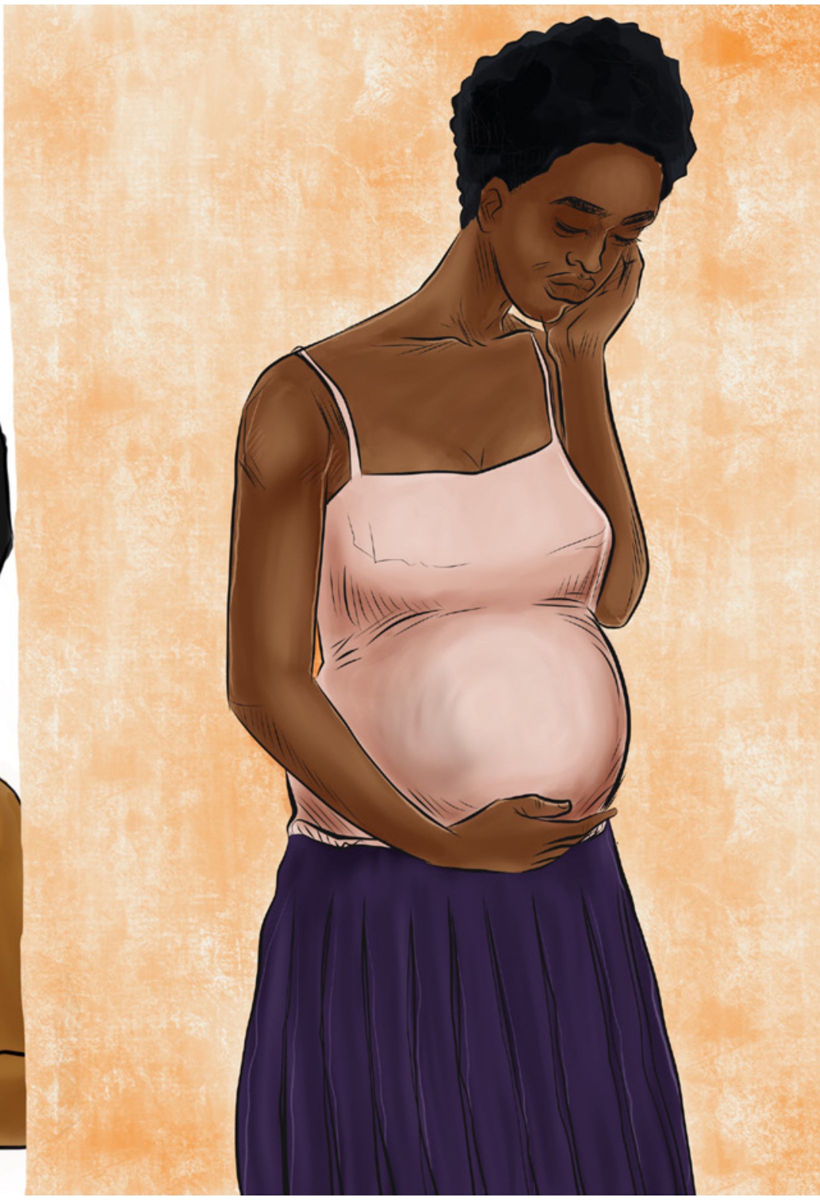

Inda yonka buhoro cyangwa itonka

Amashusho yatunganijwe muri
gahunda ya Ibaruke Neza Mubyeyi

Kwita ku babyeyi batwite no kubaha inama mu itsinda ku nshuro ya kabiri ya 2

Inda y'ibyumweru 20-24

Intego z'iri suzuma

- Gusobanurirwa ibijyanye no gukurikiranwa no guhabwa inama mu tsinda;
- Gushyiraho uburyo bwo gukurikirana amasuzuma y'ubuzima nk'itsinda;
- Kubaka icyizere n'ubusabane mu itsinda;
- Kwita ku mirire no kwiyitaho ku mubyeyi utwite;
- Kwirinda indwara;
- Kuringaniza no kuboneza urubyaro;
- Gutahura ibimenyetso mpuruza mu gihe cyo gutwita;
- Gusoza ikiganiro no gushishikariza ababyeyi kuzagaruka mu isuzuma rikurikira.

Ibikenewe muri iri suzuma

Ibyifashishwa mu gusuzuma ubuzima

- Agakoresho gapima umuvuduko w'amaraso
- Umunzani upima abantu bakuru
- Agakoresho gapima uko umutima w'umwana utera mu nda (Foetoscope)
- Agakoresho gapima umuzenguruko w'ikizigira (MUAC)
- Igipimo cy'ubushyuhe (Thermometre)
- Ikaramu na dosiye
- Isaha yagenewe kubara igihe (Horloge)

Imiti n'ibindi wakwiyegereza

- Ijage nini cyane y'amazi meza yo kunywa n'udukombe twa palasitiki dukwiriye ababyeyi bose bari buze
- Ibinini by'inzoka bya Mebendazole mg 500
- Ibinini bya feri, bigabanije mu dupaki tugenewe kamwe kuri buri muntu kakaba karimo ibinini 30 cyangwa 60

Imfashanyigisho

- Ingero z'amashusho ku mirire myiza ziri mu gaseke kabugenewe
- Amashusho mfashanyigisho: agaragaza ibimenyetso mpuruza mu gihe cyo gutwita
- Inzitiramibu

Igikorwa cya 1: Gusobanura nda yo kwita ku babyeyi batwite no kubaha inama mu itsinda

Iki gikorwa kigabanyije mu bice bitandatu kigenewe iminota 45

Igice cya 1: Gutanga ikaze

Ufashe ijambo rya mbere, aha ikaze ababyeyi bagize itsinda muri aya magambo: « Murakaza neza muri gahunda yo kwipimisha inda mu itsinda. Ikigamijwe muri iri suzuma, ni ukwiga, gusangira no gufashanya mu gihe cyo gutwita. Muzakorerwa isuzuma ryose kandi muhabwe n'ubuvuzi bwose bw'ingenzi butuma mugira ubuzima buzira umuze mu gihe mutwite. Igihe mwitabiriye kwisuzumisha nk'itsinda nibwo mwiga byinshi kurushaho kandi ku buryo bushimishije ».

Igice cya 2: Umukoro-ngiro

Abagize itsinda bicara ku ruziga barebana. Buri wese atekereza ku kintu akunda gukora agatekereza n'akantu yakora kagaragaza icyo kintu. Urugero, umubyeyi ashobora kuba akunda gusenga, ubwo rero afatanya ibiganza bye nk'usenga. Undi mubyeyi ashobora kuba akunda kumva umwana we akina mu nda igihe yirambitse aruhuka, akaba rero yakina asa n'uwirambika ngo asinzire.

Uyoboye ikiganiro abanza kubanyuremo buri wese amubwira igikorwa akunda anamwereka uko yacyigana. Buri wese amaze kuvuga icyo yagiye yigana, noneho ahera ku murongo abagize itsinda bose bagenda bavuga amazina yabo, ndetse banigana igikorwa yakoze mu kugaragaza icyo akunda.

Igice cya 3: Amabwiriza agenga itsinda

Uyoboye ikiganiro abwira ababyeyi ati: « Ni ngombwa ko buri mubyeyi yumva yisanzuye gusangira n'abandi ibitekerezo bye, ibyiyumviro bye ndetse n'ibibazo yifuza kubaza. Kubera ko rero tuzahura inshuro zigera kuri enye cyangwa buri byumweru 8, reka dushyireho amabwiriza azagenga itsinda ryacu, ajyanye n'ukuntu twafasha buri mubyeyi kumva yisanzuye mu gusangira n'abandi ibitekerezo hano. Ni ayahe mabwiriza mwumva yaba meza ku itsinda ryacu rero? ».

Mubiganireho mu minota 5. Ikigamijwe muri iki kiganiro ni ugusobanura ko:

- Ibyaganiriweho mu itsinda bigirwa ibanga; nta n'umwe wemerewe kubwira amakuru y'undi umuntu uri hanze y'itsinda kandi abagize itsinda batozwa kugirirana icyizere;
- Buri mubyeyi agomba kugira uruhare mu biganiro;
- Buri mubyeyi agomba kubahwa kandi ibitekerezo bye ni ingenzi.

Igice cya 4: Kubara inkuru

Uyoboye ikiganiro abwira ababyeyi ati: « Mu minota 30 ikurikira, umuforomo/umubyaza azajya ahamagara buri wese, umwe umwe, kugira ngo amusuzume hariya inyuma hateganijwe hihereye kugira ngo yumve uko umutima w'umwana utera kandi agire inama zihariye buri mubyeyi. Buri wese azajya ahabwa iminota itatu. Mu gihe buri mubyeyi agitegereje ko umwanya we ugerwaho, umujyanama w'ubuzima araba amufasha gupima umuvuduko w'amaraso n'ibiro. Umubyeyi ushaka kunywa amazi cyangwa kujya kwihagarika nawe yabikora. Mu gihe buri mubyeyi agitegereje ko bamugeraho ngo usuzumwe, byaba byiza agize undi mubyeyi, mu itsinda rye, baganira bakaba bahana ubuhamya ku byaba byarababayeho kuri iyi nda bitandukanye n'ibyo bari biteze. Noneho mwumve niba hari abandi mu itsinda baba barahuye n'ibisa na byo ».

Igice cya 5: Gusuzuma umugore utwite

Kora isuzuma ryuzuye nk'uko biteganijwe ahari umutwe uvuga ngo «**Isuzuma ry'ubuzima bw'umubyeyi utwite** ».

Igice cya 6: Ikiganiro

Mu gihe ababyeyi bose bagize itsinda barangije gusuzumwa umwe umwe, abayobora ibiganiro bombi basanga ababyeyi aho bicaye, bakicara bateganye mu kiganiro kugira ngo bashobore kuba bahererekanya ubutumwa batavuze (urugero ni nko kwikiriza bakoresheje umutwe) kandi bigafasha abagize itsinda bose kwiyumvamo ko bangana.

Umuforomo/umubyaza niwe uyobora ikiganiro ku bibazo ababyeyi bamubajije igihe yabasuzumaga atavuze umubyeyi wabajije ikibazo iki n'iki. Mu gusoza ikiganiro, abaza niba mu itsinda hari uwaba afite ikindi kibazo. Iki kiganiro kirakomeza ariko ntikirenze iminota 10.

Igikorwa cya 2: Ni ikihe kintu kiboneye cyo kurya no kunywa igihe umubyeyi atwite? (Imirire)

Igihe: Igikorwa cya kabiri kimara iminota 15

Intego z'iki gikorwa

Iki gikorwa gifite intego zikurikira:

- Gusobanukirwa indyo yuzuye ya ngombwa kugira ngo umubyeyi utwite n'umwana we bagire ubuzima bwiza;
- Gushishikariza umubyeyi gufata buri munsi ibinini bya aside folike na feri nk'inyunganiramirire;
- Gushimangira ko imboga zirimo vitamin A zituma umubyeyi agira ubuzima bwiza akaba ari nziza kuzirya;
- Kugira umubyeyi inama yo kwirinda kurya bikabije;
- Kwigisha ububi bwo gufata ibisindisha, ibiyobyabwenge n'ibindi byagira ingaruka mbi ku buzima bw'umubyeyi n'umwana atwite;
- Kugira umubyeyi inama yo kwirinda gukoresha imiti ya gakondo igihe atwite;
- Gushishikariza umugore gukora imyitozo ngororamubiri yoroheje.

Uko umuyobozi w'ibiganiro yitegura

- Asoma neza kandi asobanukirwa aya mabwiriza;
- Ashyira mu gaseke kabugenewe ibikoresho byose ari bwifashishe.

Ibikenewe

- Ingero umuyobozi yatanga z'ibiribwa bitera ubuzima bwiza
- Ingero z'imiti ya gakondo abantu bajya bakoresha rimwe na rimwe igihe batwite

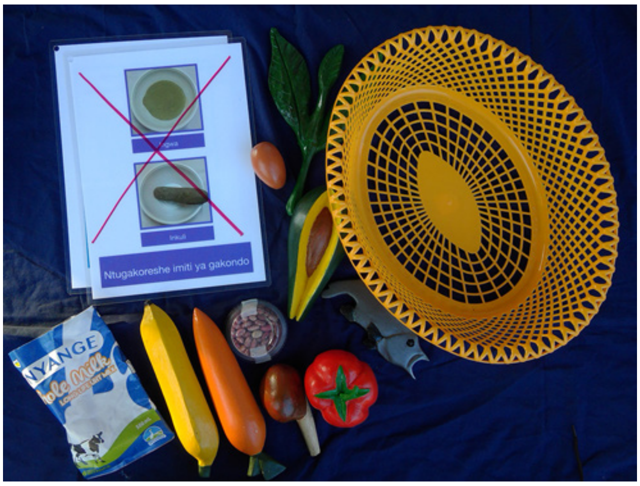

Igice cya mbere

Ugiye gutanga ikiganiro afata igikoresho yakusanyirijemo ibiri bumufashe kugitanga, ababyeyi bakagenda bagihererekanya, umwe umwe afatamo ikintu kimwe.

Igice cya kabiri

Uyoboye ikiganiro asonabura iyi ngingo muri make agira ati: « Buri kintu dushyira mu kanwa mu gihe dutwite kiba gifite ububasha bwo kutugirira neza cyane cyangwa kwangiza cyane umwana uri mu nda. Tugiye kuganirira hamwe ku bintu byiza umubyeyi agomba kurya cyangwa kunywa igihe atwite, ndetse turebe n'ibyo abujijwe kurya cyangwa kunywa igihe atwite ».

Umuyobozi w'ikiganiro asaba buri mubyeyi kuvuga izina ry'ikintu afashe mu ntoki; abaza abagize itsinda agira ati: « Icyo ufite ni cyiza cyangwa kibi kukirya cyangwa kukinywa igihe utwite?».

Igice cya gatatu
Umuyobozi w'ikiganiro abonye gisa n'icyacogoye, akurikizaho ibibazo nk'ibi bikurikira:

- Kuki ababyeyi bamwe babirya? (ibiryo, imfashamirire ya feri);
- Kuki ababyeyi batabifata n'ubwo ari byiza ku buzima kandi biryoha? (imboga, amazi);
- Kuki ababyeyi babifata? (Imiti ya gakondo cyangwa kwivura);
- Hari ingorane yateza (inkuri/inkuri, ingwa, igitaka);
- Byashoboka kurya cyangwa kugwa ivutu igihe utwite? Kubera iki?
  - » Aha umuyobozi w'ikiganiro ashimangira ko niba umugore yarabyibushye cyane igihe atwite, bishoboka ko yaba arya birenze urugero kandi bikaba byateza ingorane ku buzima bwe ndetse n'ubw'umwana atwite.

Igice cya kane (Niba hari akanya)
Uyoboye ikiganiro abaza abagize itsinda agira ati: « Muri mwe hari abazi indirimbo ijya ica kuri radio ivuga ngo "Imbaraga z'iminsi igihumbi ?" ». Akabasaba kuyiririmbira hamwe.

Igikorwa cya 3: Kwirinda no gukumira uburwayi

Igihe: Iki gikorwa kimara minota 15

Intego z'igikorwa:
- Kwibukiranya ingorane zaterwa n'uburwayi mu gihe umuntu atwite;
- Gushishikariza ababyeyi gukoresha inzitiramubu iteyemo umuti, harimo no kuyishyira ku buriri uko bikwiye;
- Kuganira ku ndwara zifata mu myanya ndangagitsina, uko zirindwa n'uko zivurwa;
- Gutahura indwara zandurira mu byo kurya.

Imyiteguro y'uyobora ibiganiro:
- Kwimenyereza iyi nkuru n'ibibazo byo kuganiraho.

Ibikoresho/ibikenerwa:
- Inzitiramibu

Igice cya mbere: Uyoboye ikiganiro abwira abagize itsinda aka gakuru
Hashize igihe gito umugabo witwa Kamanzi ashakanye n'umugore w'umunyabwenge. Mu gihe gito bashakanye, umugore yamenye ko atwite niko kubigeza ku mugabo we Kamanzi kandi bombi barabyishimira cyane. Kamanzi yabwiye umugore we ati: « Tuzagira umuryango munini cyane! ». Umugore we aramusubiza ati: « Ndifuza kugira abana bakomeye kandi bafite ubuzima bwiza, bityo uyu mwana navuka tuzategereza imyaka 2 cyangwa 3 tubone kubyara undi ». Kamanzi atungurwa n'ubumenyi umugore we afite.

Umunsi umwe, umugore yagiye kuvoma amazi meza; mbere y'uko agenda abwira Kamanzi ati: « Ngusigiye ifunguro uri bufate, ariko ntiwibagirwe gukaraba intoki n'isabune mbere yo gufungura ». Kamanzi ntiyigeze akaraba intoki, yewe ntiyigeze anapfundikira ibyo kurya, n'uko arwara impiswi araremba, ariko umugore we amwitaho mu minsi mikeya aba amaze gukira.

Umugore wa Kamanzi yasubiye kuvoma amazi, mbere yo kugenda arongera abwira Kamanzi ati: « Mu gihe mba nagiye, nagusabaga ko wamanika inzitiramibu ku buriri; sinshaka ko umubu utera Malariya waza kunduma ntwite kuko bishobora kugira ingaruka mbi cyane kuri njye no ku mwana ntwite». Atashye yinjiye mu nzu, abona umugore atazi asohotse mu cyumba cyabo yihuta cyane naho Kamanzi ari imbere mu cyumba yambaye ubusa. Umugore wa Kamanzi yaketse ko umugabo we amuca inyuma n'uko yihutira ku kigo nderabuzima ngo apimwe amaraso banamupime imyanya myibarukiro barebe ko nta ndwara yaba yaratewe n'uwo mugabo we umuca inyuma.

Agarutse mu rugo, yimanikiye inzitiramibu hejuru y'uburiri bwe, ayishyiraho neza we ubwe. Abwira Kamanzi ko badashobora kongera kuryama mu buriri bumwe, keretse abanje kujya ku kigo nderabuzima kwisuzumisha indwara. N'uko akaraba intoki, afata ibyo kurya bye bya nijoro, birimo ibishyimbo, isombe, ibijumba, n'ifi yitwa tilapia, afata ibinini bya aside folike na feri, hanyuma araryama asinzirira mu nzitiramibu.

Igice cya 2
Muri iki gice uyoboye ibiganiro abaza abagize itsinda ikibazo gikurikira agira ati: « Ibyo umugore wa Kamanzi yavuze n'ibyo yakoze mubitekerezaho iki? » Mubiganireho.

Igice cya 3
Uyoboye ikiganiro ashyira inzitiramibu hasi hagati mu ruziga akayirambura; asaba buri mubyeyi gufata ku musozo wa ya nzitiramubu kugira ngo bose bafatanye kuyikurura. Akavuga ati: « Iyi nzitiramibu ituma imibu itakugeraho bityo ikakurinda Malariya, indwara mbi cyane». Arabaza ati: «Ni akahe kaga gashobora kugera ku mubyeyi n'umwana uri mu nda, umubyeyi aramutse yanduye Malariya, n'iyo yaba atariyumvamo uburwayi? ». Aha ababyeyi umwanya wo gutanga ibisubizo noneho akaza kubabwira ibisubizo by'ukuri, birimo kuba umwana yapfa mbere cyangwa nyuma yo kuvuka, kuvuka adashyitse, kuvuka ari muto cyane cyangwa se ananiwe.

Uyoboye ibiganiro arongera akabza ati:« Dukoresha dute inzitiramibu mu ngo zacu?». Umuyobozi w'ikiganiro arongera agasubiramo neza, asobanura ukuntu bamanika inzititamibu neza hejuru y'uburiri, n'igihe bagomba kuyihindura. Agasobanura ko ubusanzwe igihe imara kiba cyanditse ku nzitiramibu iteyemo umuti; icyo gihe kiba kiri hagati y'umwaka 1 n'imyaka 3.

Igice cya 4

Umuyobozi w'ikiganiro arabaza ati: « Ni iki umugore wa Kamanzi yakoze kugira ngo yirinde uburwayi?». Akongera agasaba abagize itsinda ko babiganireho. Ashobora gukomeza kubabaza ibibazo bikurikira:

- Ni iki wakora kugira ngo urinde umuryango wawe uburwayi?
  - » Gukaraba intoki;
  - » Isuku n'isukura;
  - » Gukoresha amazi asukuye;
  - » Kuronga imboga zavuye mu murima n'amazi asukuje umuti wabugenewe, kutarya ibyo kurya bidahiye neza, gushyushya cyane ibyo kurya mbere yo kubifungura;
  - » Kujya kwa muganga ako kanya ukimara gufatwa n'uburwayi.
- Gukora imibonano mpuzabitsina utwite mubitekerezaho iki?
  - » Ntacyo bitwaye?
  - » Ni gute wirinda indwara zandurira mu mibonano mpuzabitsina?
  - » Ubudahemuka ku bashakanye bombi, kwifata, agakingirizo.
  - » Niba Kamanzi n'umugore we barasanze nta gakoko gatera SIDA bafite mu gihe cyo kwisuzumisha inda ku nshuro ya mbere, ntibashobora kwandura mu gihe umugore we agitwite?
    - › Yego. Niba umugabo cyangwa umugore bakiryamana n'abandi bantu, umugore n'umwana atwite bashobora kwandura agakoko gatera SIDA;
    - › Uburyo rukumbi bwo kwirinda izo ngaruka ni ugukoresha agakingirizo igihe cyose ukora imibonano mpuzabitsina.
  - » Wabona udukingirizo ute? Ni gute wateganya gukoresha agakingirizo ufatanije n'uwo mwashakanye?

Mu gihe bishoboka, uyoboye ikiganiro agenda ahereza ababyeyi udukingirizo tw'abagabo, haba hari n' abagabo baje mu itsinda nabo akatubaha. Ashaka uko abereka uko bambara neza agakingirizo k'abagabo yifashishije igitsina cy'umugabo cy'igikorano (Imfashanyigisho).

Igice cya 5

Umuyobozi w'ikiganiro yongera kubaza ikibazo gikurikira: « Umugore wa Kamanzi afite umugambi wo gutegereza imyaka 2 cyangwa 3 kugira ngo abone kubyara undi mwana? ». Ababaza icyo batekereza kuri iki kibazo.

Igikorwa cya 4: Ibimenyetso mpuruza ku mugore utwite

Igihe: Iki gikorwa kimara minota 15

Intego z'iki gikorwa

- Gusubiramo ibimenyetso mpuruza n'ibintu bishobora gutera ingorane ku mugore utwite;
- Gusuzuma ubumenyi bafite no kubigisha igihe bibaye ngombwa;
- Gushishikariza ababyeyi guhita bajya kwivuza kwa muganga igihe bafite ibimenyetso mpuruza.

Imyiteguro y'uyobora ibiganiro

- Gusoma no kumva neza ibikubiye muri iki gikorwa;
- Gukusanya amashusho mfashanyigisho yo gukoresha muri iki gikorwa.

Ibikoresho/ ibikenewe: Amashusho mfashanyigisho yerekana ibikurikira «Ibimenyetso mpuruza k'umugore utwite»

Igice cya 1

Uyoboye ikiganiro aravuga ati: « Buri wese nafate ishusho imwe muri izi ngiye kubaha ». Atanga ya mashusho, bakayahererekanya azenguruka uruziga, buri mubyeyi afata ishusho imwe.

Igice cya 2

Buri mubyeyi agenda yigana ingorane abonye ku ikarita yafashe; uyoboye ikiganiro nawe agakora ku buryo kuri buri kimenyetso-mpuruza ibi bibazo bikurikira biganirwaho:

- Ni ibiki birimo kuba kuri uyu mubyeyi?
- Ni izihe ngaruka ibirimo kumubaho bishobora kumugiraho?
- Umubyeyi yabikoraho iki cyangwa se umuryango we wabikoraho iki?"

Umusozo: Kurushaho kumenyana no kunga ubumwe

Igihe: Igikorwa cyo gusoza ikiganiro kimara iminota 5

Intego z'iki gikorwa

- Gushimangira ko ababyeyi barushaho kunga ubumwe cyane kandi bagafashanya;
- Kwibutsa ababyeyi ko bazagaruka mu rindi suzuma ry'itsinda uko bari bari kumwe muri iri tsinda, nyuma y'ibyumweru 8;
- Abagize itsinda bemeza niba abagabo babo cyangwa abandi bafitanye isano ya bugufi bazatumirwa mu isuzuma ry'ubutaha;
- Kwibutsa ababyeyi kwitabira ubwisungane mu kwivuza.

Imyiteguro y'uyobora ikiganiro

- Kwimenyereza ibikubiye muri iki kiganiro;
- Gutegura igipupe n'umupira ubanze mu budodo.

Ibikoresho/ibikenewe: igipupe n' umupira ubanze mu budodo

Igice cya 1: Kwemeza gutumira abagabo babo cyangwa abandi bafitanye isano ya bugufi mu masuzuma ataha

Umuyobozi w'ikiganiro asaba ababyeyi guhagarara ku ruziga; akabashimira kuba bitabiriye gahunda y'uwo munsi, anababwira muri make ubutumwa bukubiye mu isuzuma bagize uwo munsi. Abibutsa ko biyemeje ko ibyavugiwe mu isuzuma ry'uwo munsi byose bigirwa ibanga. Akomeza abaza ababyeyi ati: « Gutumira abagabo banyu cyangwa abandi mufitanye isano ya bugufi mu masuzuma y'ubutaha murabivugaho iki ? ». Ababwira ko gutumira abashyitsi mu masuzuma ataha aribo bigomba guturukamo. Akababwira ko mu isuzuma rikurikira, bazaganira ku bijyanye no kuboneza urubyaro ndetse n'imyiteguro yo kubyara. Ababaza uko babyumva; niba baguma bonyine cyangwa cyangwa b azatumira abagabo babo n'abo bafitanye isano rya bugufi mu kiganiro. Abaha umwanya wo kubyunguranaho ibitekerezo, nyuma y'umunota 1 bagafata umwanzuro kuri ubwo butumire.

Igice cya 2: Gushyiraho urubuga rubahuza

Uyobora ibiganiro afata umupira ubanze mu budodo; agasaba buri mubyeyi kuvuga muri make isomo akuye mu kiganiro bagize uwo munsi, n'uburyo azazishyira mu bikorwa kugira ngo agire ubuzima buzira umuze. Vuga ikintu kigufi waba wize uzigisha undi muntu mu buzima bwawe. Noneho afata umupira ku kadodo k'inyuma gasaguka agakomeza ntakarekure. Anagira uwo mupira w'ubudodo undi muntu wo mu itsinda, abanje kumureba mu maso kugira ngo arebe ko yiteguye kuwufata. Uruhererekane rugenda neza cyane iyo buri wese umupira umugeraho nawe agahita awunaga vuba; ashobora gutangira awunagira uwo bafatanyije mu kuyobora ibiganiro niba ahagaze aho bateganye, kugira ngo n'ababyeyi barebereho uko bikorwa.

Igice cya 3

Asaba umuntu wafashe umupira kuvuga ikintu yungutseho ubumenyi mu isuzuma ry'uwo munsi mu itsinda cyangwa se icyo yazigisha undi muntu akamenya amakuru y'ukuri. Noneho anagira umupira undi muntu ku ruziga, afata agashumi gasaguka akagakomeza. Buri muntu agenda afata akanya ko gutanga igitekerezo ubundi akawunagira mugenzi we kugeza hagiyeho akantu kameze nk'agatanda gakozwe n'urusobe rwa bwa budodo ababyeyi bagiye basigarana mu ntoki.

Igice cya 4

Umuyobozi w'icyo gikorwa asaba abagize itsinda kugarura uwo mupira ubanze mu butambaro umaze guhetura ababyeyi bose. Ababwira ko amasuzuma arangira, ababyeyi bose bamaze kuba inshuti kandi buri wese afite ibyo yagiye yigira kuri mugenzi we. Asobanura ko noneho bose bamaze kunga ubumwe bahujwe n'ako kantu gasa n'agatanda kakozwe n'urusobe rw'ubudodo kandi buri wese yabigizemo uruhare.

Igice cya 5

Uyoboye icyo gikorwa ashyira igipupe ku gatanda k'ubudodo kugira ngo kibe gishyigikiwe neza kitagwa. Akababwira ati: « Muratekereza iki kuri kano gatanda twakoze? ». Iyo habuze usubiza, arabahwitura akabasobanurira ukuntu bafatanyije barushaho kugira ingufu mu kwiyitaho ubwabo, kwita kuri bagenzi babo ndetse no kwita ku bana babo.

Igice cya 6: Umusozo

Umuyobozi w'ikiganiro akura cya gipupe kuri ka gatanda. Akabwira ababyeyi kumanura utudodo bari bafashe mu ntoki badushyira hasi imbere y'ibirenge byabo ariko bakabikorana ubwitonzi ku buryo bwa budodo bugize agatanda budasobana. Mu gihe bamanura utwo tudodo, abibutsa ko bazongera bagahura nyuma y'ibyumweru 8 baje kwisuzumisha kugira ngo baganire ku ngingo z'ingirakamaro zigendanye no gutwita. Abasonanurira ko isuzuma rizaba risa n'iry'uwo munsi; hazakorwa isuzuma, ubuvuzi n'ibiganiro kandi akabashishikariza kuzaza nta n'umwe ubuze nk'uko batangiranye itsinda.

Yibutsa ababyeyi itariki n'isaha itsinda rizongera guhuriraho; akabibutsa ko bagomba kubika neza ikarita yo kwisuzumishirizaho inda kandi bakazayizana mu isuzuma ry'ubutaha. Ababwira ko haramutse hagize urwara, ashobora kuza igihe icyo ari cyo cyose, ariko igihe ari bazima bagomba gutegereza isuzuma ry'ubutaha igihe bazaba bazanye n'itsinda ryabo, ari ryo riri ku gihe cyashyizweho cyo kuzongera guhura. Mu gusoza, uyoboye ikiganiro yibutsa ababyeyi kwitabira ubwisungane mu kwivuza.

Igice cya nyuma

Iyo hari ukeneye ubufasha bwihariye, nko gusuzumirwa ahiherereye, gufatwa amaraso cyangwa guterwa urushinge, uyoboye ikiganiro amubwira icyo yakora. Hari igihe byaba ngombwa ko asigara kugira ngo yitabweho wenyine.

Mu gihe ababyeyi bose bamaze kugenda, abayoboye isuzuma n'ibiganiro buzuza igitabo cyagenewe ibijyanye no kwita ku babyeyi batwite no kubagira inama.

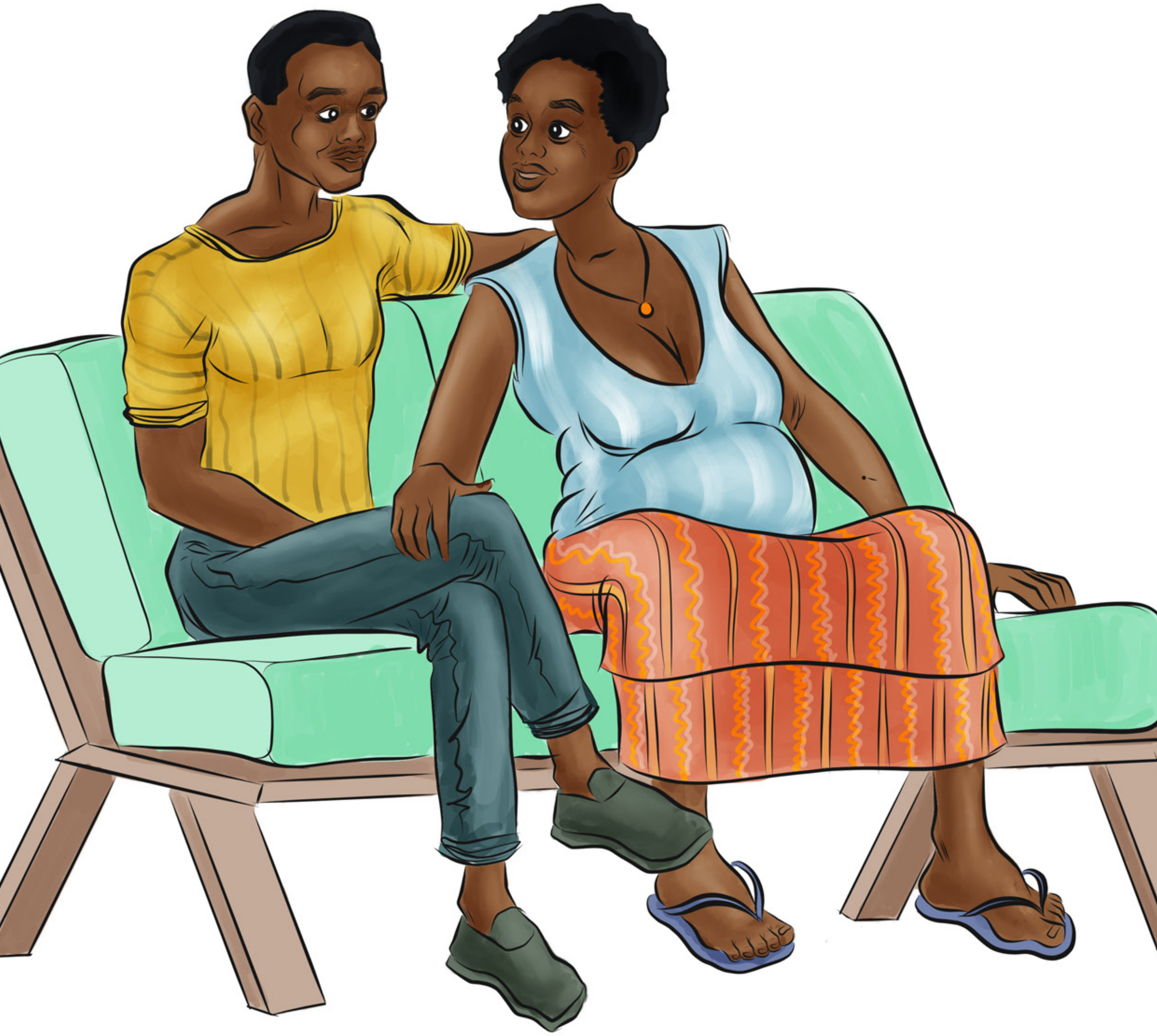

Guhitamo uburyo bwo kuboneza urubyaro
mbere y'uko ubyara—nk'uburyo bwo
kuboneza urubyaro wafata ukimara kubyara

Amashusho yatunganijwe muri
gahunda ya Ibaruke Neza Mubyeyi

Kwita ku babyeyi batwite no kubaha inama mu itsinda ku nshuro ya kabiri

Inda y'ibyumweru 28-32

Intego z'iki gikorwa

- Gusuzuma ubuzima bw'ababyeyi;
- Kongera icyizere n'ubusabane mu itsinda;
- Kuganira ku buzima bwo mu mutwe bw'umubyeyi utwite na nyuma yo kubyara;
- Imyiteguro yo kubyara n'ibimenyetso by'uko umubyeyi ari ku nda
- Kuringaniza no kuboneza urubyaro, by'umwihariko gahunda yo kuruboneza umuntu akimara kubyara (PPFP);
- Ibimenyetso mpuruza ku mubyeyi utwite;
- Umukoro-ngiro no guhamagarira ababyeyi kuzitabira isuzuma ry'ubutaha mu itsinda.

Ibikenewe muri iri suzuma

Ibikoresho bikoreshwa mu gusuzuma ubuzima bw'umubyeyi ni ibi bikurikira:

- Agakoresho gapima umuvuduko w'amaraso;
- Umunzani upima abantu bakuru;
- Agakoresho gapima uko umutima w'umwana utera mu nda (Foetoscope);
- Agakoresho gapima umuzenguruko w'ikizigira cy'ukuboko (MUAC);
- Igipimo cy'ubushyuhe (Thermometre);
- Ikaramu na dosiye z'ubuvuzi;
- Isaha yagenewe kubara igihe (Horloge), si ngombwa cyane;

Imiti n'ibindi wakwiyegereza:

- Ijage nini cyane y'amazi meza yo kunywa n'udukombe twa palasitiki dukwiriye abagore bose bari buze;
- Ibinini by'inzoka bya Mebendazole mg 500;
- Ibinini bya feri, bigabanije mu dupaki tugenewe buri muntu kamwe kakaba karimo; ibinini 30 cyangwa 60;
- Inkingo za Tetanosi.

Imfashanyigisho

- Ingero z'amarangamutima/imbamutima cyangwa amashusho;
- Ingero z'imyiteguro yo kubyara;
- Amashusho agaragaza ibimenyetso byo kujya ku nda;
- Ingero z'uburyo butandukanye bwo kuboneza urubyaro;
- Amashusho y'ibimenyetso mpuruza ku mubyeyi utwite.

Gutangira gusuzuma ababyeyi bakihagera

Igihe: Gusuzuma ababyeyi bimara iminota 30

1. Iyo ababyeyi bahageze, ushinzwe kubasuzuma no kubaha inama abakirana urugwiro, agahita atangira kubasuzuma;
2. Aganiriza ababyeyi mu buryo bukurikira mu gihe bagitegereje ko umuforomo/umubyaza aza kubasuzuma. Arababwira ati: « Mu gihe mugitegereje gusuzumwa n'umuforomo/umubyaza, buri mubyeyi aganire na mugenzi wawe begeranye ku kintu cyamubangamiye ku nda atwite. Amubwire ikintu cyaba cyaramuguye nabi. Buri wese abaze mugenzi we niba nawe yarahuye n'ikibazo nk'icyo; munaganire n'uko mwabyitwayemo.
3. Akora isuzuma ry'ubuzima bwa buri mubyeyi arirangize (nk'uko bisobanuye mu ntangiriro z'iyi nyandiko).
4. Ashishikariza ababyeyi kunywa amazi igihe bakiri muri iki kiganiro.

Ikiganiro kibanza

Igihe: Iki kiganiro kimara iminota 10

Igice cya 1: Amabwiriza agenga itsinda

Uyoboye ikiganiro abaza ababyeyi niba bibuka amabwiriza bashyiriyeho itsinda ryabo igihe bahuraga bwa mbere; ababyeyi bagombye gutanga ibisubizo bakanasubiramo amabwiriza agenga itsinda ryabo, babiganiraho.

Ikigamijwe muri iki kiganiro ni ugusobanura neza ko:

- Ikintu cyose cyaganiriwe mu itsinda kigirwa ibanga;
- Buri mubyeyi agomba kugira uruhare mu biganiro;
- Umuyobozi w'ikiganiro agomba gutega amatwi kandi akubaha buri mubyeyi.

Igice cya 2: Ikiganiro kigendeye ku gakino k'ababyeyi

Kuba ababyeyi bagize itsinda barahuye ku nshuro ya kabiri muri gahunda yo kwitabira no kubagira inama bikorewe mu itsinda, ntibibujije ko kuri iyi nshuro hashobora kuba hari abaje ubwa mbere cyangwa se abatumirwa (abashakanye n'ababyeyi bagize itsinda cyangwa abo bafitanye isano ya bugufi). Iki gikorwa kigomba gukomeza kurema icyizere n'ubufatanye mu itsinda.

Abagize itsinda bicara ku buryo bw'uruziga barebana; buri wese atekereza ku kintu akunda gukora n'igikorwa cyagaragaza icyo akunda. Dufate nk'urugero, umubyeyi umwe ashobora kuba akunda gusenga, ubwo afatanya ibiganza bye nk'usenga. Undi mubyeyi ashobora kuba akunda kumva umwana we akina mu nda igihe aryamye afata akaruhuko; ubwo akaba yakina asa n'uryamya ngo asinzire. Uyoboye ikiganiro agenda abanyuriramo, buri wese amubwira igikorwa akunda anamwereka uko yacyigana.

Buri wese iyo amaze kuvuga icyo yagiye yigana, uyoboye ibiganiro ahera ku murongo akagenda abaza abagize itsinda kugenda bavuga amazina ya buri muntu ndetse baganire ku gikorwa yakoze mu kugaragaza icyo akunda.

Igice cya 3: ibibazo n'ibisubizo by'ababyeyi

Umuyobozi w'ikiginiro azirikana ibibazo ababyeyi babajije igihe basuzumiraga ahiherereye uwo munsi, akabizana mu itsinda ryose kugira ngo arebe niba ababyeyi bazi ibisubizo byabyo; yasanga batabizi akabaho amakuru akwiriye. Abaza ababyeyi niba hari icyo buri wese yaba yigiye kuri mugenzi we baganiriye uwo munsi ndetse n'uko bahangana n'ibibazo ababyeyi batwite bakunze guhura nabyo. Abasangiza amakuru akwiye niba ari ngombwa.

Igikorwa cya 1: Ubuzima bwo mu mutwe

Igihe: Iki gikorwa kimara iminota 15

Intego z'iki kiganiro

- Gusobanukirwa impamvu ababyeyi bagaragaza ibimenyetso byo kwiheba no guhangayika;
- Gutahura ibimenyetso biteye impungenge niba hari ibihari;
- Kuganira ku cyo ababyeyi n'imiryango bashobora gukora igihe ibimenyetso nk'ibyo bigaragaye; Kuganira ku buryo ababyeyi bakwifashisha kugira ngo bagire ubuzima bwo mu mutwe bwiza.

Imyiteguro y'uyobora ibiganiro

Uyoboye ikiganiro asoma kandi akumva neza ibikubiye mu kiganiro kandi akegeranya ingero z'uburyo bunyuranye bwo kuboneza urubyaro, akazishyira mu gikoresho cyabugenewe.

Ibikoresho/ibyifashishwa

Ingero z'amarangamutima mu gaseke kabugenewe cyangwa amashusho ku miterere y'amarangamutima.

Igice cya 1

Uyoboye ikiganiro aha ikaze ababyeyi bagize itsinda; atembereza agaseke yashyizemo ya mashusho mfashanyigisho mu babyeyi bose bicaye ku buryo bw'uruziga, buri wese agafatamo ishusho. Abwira abagize itsinda ko ababyeyi bamwe batwite bakunda kugira agahinda n'ubwoba muri bo. Ibyo bikaba biterwa n'uko baba badafite ubuzima bwiza. Akomeza ababwira ati: « Nimureke tuganire kuri iyo mimerere y'umubyeyi utwite kugira ngo twumve neza icyakorwa ».

Igice cya 2

Uyoboye ikiganiro abaza buri mubyeyi ku rugero yafashemo, akabaza umwe umwe ikibazo gikurikira: « Ni ayahe marangamutima ubona agaragazwa muri urwo rugero wahisemo? ». Arongera akamubaza ikindi kibazo gikurikira, agira ati: « Iyo ufite amarangamutima nk'ayo wumva umerewe ute mu mubiri wawe? ». Ashishikariza abandi babyeyi kugira icyo bavuga kuri ayo marangamutima kugira ngo umubyeyi ufashe iyo nshusho ataba ariwe wenyine uyavugaho.

Igice cya 3

Umuyobozi w'ikiganiro abaza ababyeyi ati: « Muri mwe haba hari uwigera agira amarangamutima nk'aya kuva yasama? ». Asaba buri mubyeyi wese wasubije abyemeza gusobanura birambuye uko byamugendekeye. Aramubaza ati: « Byagenze bite? ». Akongera kumubaza ati: « Ukora iki iyo wumva umerewe gutyo?». Niba ubona abayeyi batinya kuvuga, ashyira izo ngero mu kiganiro. Ashishikariza ababyeyi kubiganiraho, akemeza niba uko baba biyumva ari ibisanzwe. Abaganiriza mu magambo make, ibintu bisanzwe bikunze guhinduka mu marangamutima igihe umubyeyi atwite.

Igice cya 4

Umuyobozi w'ikiganiro abaza ababyeyi ati: « Muri mwe hari uwaba azi umubyeyi wari utwite cyangwa nyuma yo kubyara akagira kwiheba, agahangayika cyangwa akagira guhungabana mu bitekerezo? ». Abaza ababyeyi basubije gusobanura neza uko uwo mubyeyi babonye yitwararga. Arababaza ati: « Uwo muntu mwabonaga yitwaye ate cyangwa yavugaga iki? ». Arongera akabaza ati: « Muri ibi bikurikira ni ikihe kintu kihariye cyatumye ubona ko uwo mubyeyi atari ameze neza: kwiheba, guhangayika cyangwa guhungabana mu bitekerezo? ». Niba ababyeyi batinye kuvuga, ababaza uwaba afite ishusho yababera urugero rwerekana neza uko umubyeyi urembye yaba agaragara.

Igice cya 5

Umuyobozi w'ikiganiro abaza ababyeyi ati: « Mutekereza ko ari iyihe mpamvu yatuma umuntu agira ibibazo byo guhungabana mu mitekerereze ye n'ubuzima bwe bwo mu mutwe? ». Abasaba kuganira ku mpamvu

zinyuranye zishobora kubitera, arizo: impinduka zikomeye mu mikorere y'ubwonko, ihungabana, kubura umuntu w'ingenzi mu buzima bwe cyangwa ikintu cy'agaciro n'ibindi. Ashishikariza ababyeyi kuganira niba batekereza ko izo mpinduka mu marangamutima no mu mubuzima bwo mu mutwe zishobora kuvurwa ndetse niba bazi aho bashobora kubonera ubuvuzi nkenerwa. Abasaba kuganira ku ihezwa n'ubwoba bishobora gutuma badasaba ubufasha.

Yongera kubabaza ati: « Uramutse wiyumvise gutyo, wabyifatamo ute? ». Arongera akabaza ati: « Ni iki umuryango wawe cyangwa abandi bantu bo muryango bagufasha? ». Asoza abasaba kuganira ku bufasha, uko umuntu ufite ibibazo byo mu mutwe yarindwa akato n'uko yabona ubuvuzi.

Igice cya 6 (Yabikora ku bushake niba afite umwanya)

Niba abona bikwiriye, uyoboye ikiganiro ashobora gukoresha umwitozo wo guhaguruka no gutoranya akoresheje uburyo bwo gusuzuma ihungabana ry'umubyeyi nyuma yo kubyara. Abasaba bose bahaguruke, akababwira ati: « Ngiye kugenda mvuga interuro zimwe na zimwe zijyanye n'imiterere y'amarangamutima; nimwumva nsomye urugero rw'amarangamutim mufite ku ikarita muzamure ikiganza ». Ababyeyi nibazamura ikiganza, abasabe kujya ku murongo umwe, abafite amashusho ariho amarangamutima meza ku ruhande rumwe, n'abafite amashusho ariho amarangamutima ateye impungenge ku rundi ruhande. Birangira hari imirongo ibiri ku ruziga. Aba babyeyi baba bagiye bitoranyamo uruhande rw'abantu bakomeye mu marangamutima n'uruhande rw'abafite ihungabana.

Umuyobozi w'ikiganiro asoma izi nteruro, imwe imwe:

- Nabashije guseka no kwishimira ibintu;
- Nagiye numva nishimiye ubuzima;
- Niciriye urubanza bitari ngombwa ku bintu bitagenze neza;
- Narahangayitse nkuka umutima kandi nta mpamvu ifatika;
- Numvise nikanze mfite impagarara nta mpamvu ifatika ibiteye;
- Nagiye numva ndemerewe n'ibibazo cyane;
- Nabuze umunezero cyane ku buryo nsinzira bigoranye;
- Numvise mfite agahinda kandi nkiheba ku buryo bukomeye;
- Nagize umubabaro ku buryo akenshi naniraga.
- Hari igihe numvise nakwigirira nabi ndetse nshaka no kwiyahura.

Kugeza aha ababyeyi baba bamaze kwigabanya mu mpande ebyiri: abishimye n'ababaye. Yumvisha ababyeyi ko niba hari uwagize amarangamutima menshi nk'ayo akaba nta yo kwishima afite, agomba gusaba ubufasha ako kanya. Igihe ayo marangamutima y'umubabaro akomeye cyane, ibyo babyita ihungabana kandi akaba ari uburwayi bushobora kuvurirwa ku bitaro by'Akarere.

Igikorwa cya 2: Imyiteguro yo kubyara n'ibise

Igihe: iki gikorwa kimara iminota 15

Intego z'iki gikorwa

- Kuganira ku byo buri mubyeyi agomba gukora cyangwa kumenya mu rwego rwo kwitegura igihe cyo kubyara;
- Kuganira ku buryo ababyeyi baganira n'abagabo babo n'abandi bo mu muryango ku myiteguro yo kubyara;
- Kuganira ku nyungu zo kubyarira kwa muganga.

Imyiteguro y'uyobora ibiganiro

Uyobora ikiganiro asoma kandi akumva neza ibikubiye muri iki kiganiro; yegeranya ingero z'imyiteguro yo kubyara azishyira mu gaseke yateguye.

Ibikoresho/ibyifashishwa

- Ingero z'ibikoresho by'imyiteguro yo kubyara:
  - » Akagofero k'uruhinja;
  - » Icyemezo cy'amavuko y'umwana;
  - » Agasengeri k'uruhinja;
  - » Isuwime;
  - » Igitenge kizinze neza;
  - » Ikanzu y'umubyeyi izinze neza;
  - » Ikarita y'ubwisungane mu kwivuza;
  - » Isabune;
  - » Akadobo (Gato);
  - » Igikoma (Ishashi ifunze);
  - » Agakapu ko mu ntoke;
  - » Ikintu cy'amafuti kidafite aho gihuriye n'imyiteguro yo kubyara kiri busetse ababyeyi.

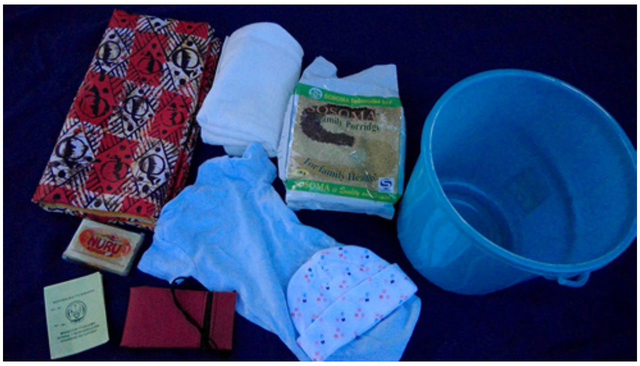

- Amashusho
  - » Umugabo cyangwa undi ufitanye isano ya bugufi n'umubyeyi umufasha kwinjira mu modoka (Ambulance) igihe ari ku nda;
  - » Amashusho agaragaza ibimenyetso by'umubyeyi ugiye kwibaruka.

Igice cya 1

Umuyobozi w'ikiganiro azengurutsa mu babyeyi ka kantu yashyizemo ibikoresho, uko bicaye ku ruziga agasaba buri mubyeyi gufatamo ikintu kimwe.

Igice cya 2

Umuyobozi w'ikiganiro avuga mu magambo make insanganyamatsiko. Abaza abagize itsinda ikibazo gikurikira: « Ni iki buri mubyeyi akora yitegura kubyarira kwa muganga? ».

Abaza buri mubyeyi izina ry'icyo afashe mu ntoki; akabaza itsinda agira ati: « Kuki ukenera icyo kintu igihe wabyaye? ».

Igice cya 3

Iyo ikiganiro gitangiye gukonja, umuyobozi wacyo abaza abari mu itsinda ibibazo bikurikira kugira ngo barusheho gusobanukirwa neza:

- Kuki ari ngombwa ko ababyeyi babikora?
- Niba warigeze kubyarira kwa muganga, ni iki wicujije uti kuki ntacyitwaje?
- Ni iki wifuza kuzaba ufite igihe uzaba wabyaye?
- Ese waba waraganiriye n'umufasha wawe ku by'imyitaguro yo kubyara? Abitekerezaho iki?
- Umuryango wawe utekereza iki ku bijyanye no kwitegura kubyara?

Igice cya 4

Umuyobozi w'ikiganiro abanyuzamo cya gikoresho cyarimo imfashanyigisho kirimo ubusa asaba buri mubyeyi gusubizamo icyo yari yafashe. Ako kanya, atangira kubahereza amashusho agaragaza ibimenyetso byerekana umubyeyi uri ku nda cyangwa watangiye kugira ibise; agasaba buri wese gufata ishusho imwe.

Igice cya 5

Ababyeyi bagenda berekana ibimenyetso babona ku makarita, umubyeyi umwe umwe. Umuyobozi akora ku buryo ingingo zikurikira zivugwa kuri buri kimenyetso cy'uko umubyeyi ari ku nda:

- Uyu mubyeyi ni iki kirimo kumabaho?
- Ni iki wakora igihe bigenze bityo cyangwa ni iki umuryango w'uyu mubyeyi wakora?

Igikorwa cya 3: Koboneza urubyaro

Igihe: Iki gikorwa kimara iminota 20

Intego z'iki gikorwa

- Kuganira ku ngaruka mbi zo kubyara indahekana byaba ku mubyeyi ndetse no ku mwana atwite;
- Gushishikariza ababyeyi gufata gahunda n'abagabo babo yo gutegereza ko umwana agira nibura imyaka 2 y'amavuko umugore akabona kongera gusama niba bateganya kubyara undi mwana;
- Kuganira no kubeshyuza amakuru ku bihuha bivugwa ku buryo bunyuranye bwo kuboneza urubyaro.

Imyiteguro y'uyobora ibiganiro

- Uyoboye ikiganiro asoma kandi akumva neza ibikubiye muri iki kiganiro;
- Ategura ingero z'uburyo butandukanye bwo kuboneza urubyaro (azishyira muri ka gaseke yateguye).

Ibikoresho/ibyifashishwa

- Ategura agaseke karimo uburyo bwose bushoboka bwo kuboneza urubyaro;
- Afata akanya akabaganiriza kuri gahunda yo kuboneza urubyaro umubyeyi akibyara (PPFP).

Igice cya 1: Ikiganiro cyifashishije inkuru

Umuyobozi w'ikiganiro atangira abaza abagize itsinda niba bibuka ko ubwo bahuraga ubushize nk'itsinda bavuze ku nkuru y'umugore wa Kamanzi; ababwira ko abafitiye indi nkuru kuri uwo mugore w'umunyabwenge.

Akomeza ababwira ko umugore wa Kamanzi yibarutse umwana mwiza cyane. Uwo mugore yari afite murumuna we washakanye n'umugabo witwa Mugenzi, nawe akaba yarabyaye muri uwo mwaka. Umugore wa Kamanzi yari amaze kubyara abana batatu; umwana wa mbere yari afite imyaka 7, uwa kabiri afite imyaka 4, naho uwa gatatu afite umwaka 1. Umugore wa Mugenzi nawe yari afite abana 3; umukuru yari afite imyaka 3, umukurikira afite imyaka 2, naho umuto afite umwaka 1. Nyuma y'iyi nkuru ababaza ikibazo gikurikira: « Muratekereza iki ku myaka y'abana ba Kamanzi n'iy'aba Mugenzi? ». Akabasaba kuganira ku bijyanye no kwirinda kubyara indahekana.

Igice cya 2

Umuyobozi w'ikiganiro abaza ababyeyi, umwe umwe, ati: « Mwebwe ibyo kuboneza urubyaro mubitekerezaho? ». Akomeza abaza ati: « Ni ikihe gihe cyiza ku buzima, bategereza mbere yo kongera gusama? ». (Imyaka 2). Yongera kubabaza ati: « Kubera iki wumva ko umubyeyi n'umwana bagira ubuzima bwiza aramutse asize intera ihagije hagati y'imbyaro? ».

Igice cya 3

Uyoboye ikiganiro abaza ababyeyi ati: « Ni gute abashakanye bashobora gufatanya muri gahunda yo kuringaniza no kuboneza urubyaro mu muryango wabo? ».

Igice cya 4

Umuyobozi w'ikiganiro abanyuzemo ka gakoresho karimo ingero z'uburyo bwo kuboneza urubyaro; buri mubyeyi agenda afatamo urugero rumwe agasa nk'uruzamuye kugira ngo abandi bagize itsinda barubone. Arababaza ati: « Iki ni iki kandi gikoreshwa gute?

». Agakora ku buryo baganira ku ngingo zikurikira kuri buri buryo bwo kuboneza urubyaro:

- Igihe ubwo buryo bwatangira gukoreshwa. Ashimangira gahunda yo kuboneza urubyaro umubyeyi akimara kubyara kandi akayisobanura ku buryo burambuye. Aha umwanya wihariye wo kurushaho gusobanurira buri wese uko bikorwa, hagira umubyeyi uhitamo kuzaboneza urubyaro akimara kubyara akaza kwandika uburyo yahisemo ku ifishi ye.
- Aho yakura ubwo buryo;
- Amafaranga ubwo buryo bwishyurwa, n'uburyo buhendutse ubwo ari bwo.

Igice cya 5
Uyoboye ikiganiro abaza ababyeyi ati: « Hari uwaba afite ikibazo ku bijyanye no kuboneza urubyaro? ». Ikibazo babaza akigarurira itsinda ryose kugira ngo bagishakire igisubizo. Niba batabishoboye, abaha igisubizo cy'ukuri.

Igice cya 6
Umuyobozi w'ikiganiro asaba ababyeyi kuvuga ku makuru bumvise aho batuye yerekeye kuboneza urubyaro. Abashishikariza kuvuga ibyo babwiwe na bagenzi babo, ababyeyi bakuze bo mu muryango, cyangwa undi wese bumvanye amakuru atandukanye n'ibyaganiriweho mu itsinda.

Kuri buri makuru ababyeyi bavuze atariyo, akagenda abaha ibisobanuro birambuye kugira ngo baze gutahana amakuru nyayo kandi anabasaba kuzajya bayasangiza bagenzi babo bafite amakuru atari yo ku kuboneza urubyaro.

Dore ingero z'amakuru atari yo abantu bakunda kuvuga ku kuboneza urubyaro:

- Umuntu waboneje urubyaro biba birangiye ntiyongera kubyara;
- Umwana wavutse ku mubyeyi waboneje urubyaro avukana ibibazo;
- Iyo bagushyizemo agapira gashobora kukwica cyangwa kakakumugaza;
- N'ibindi.

Izi ngero zivuzwe haruguru azifashisha mu kunganira ababyeyi mu gihe batinye kugira icyo bavuga kuri iyi ngingo.

Igikorwa cya 4: Ibimenyetso mpuruza ku mugore utwite

Igihe: Iki gikorwa kimara minota 15

Intego z'iki gikorwa

- Gusubiramo ibimenyetso mpuruza n'ibintu bishobora gutera ingorane ku mugore utwite;
- Gusuzuma ubumenyi bafite no kubigisha igihe bibaye ngombwa;
- Gushishikariza ababyeyi guhita bajya kwivuza kwa muganga igihe bafite ibimenyetso mpuruza.

Imyiteguro y'uyobora ibiganiro

- Gusoma no kumva neza ibikubiye muri iki gikorwa;
- Gukusanya amashusho mfashanyigisho yo gukoresha muri iki gikorwa.

Ibikoresho/ ibikenewe: Amashusho mfashanyigisho yerekana ibikurikira «Ibimenyetso mpuruza k'umugore utwite»

Igice cya 1
Uyoboye ikiganiro aha ababyeyi abamashusho ariho ibimenyetso mpuruza ku mubyeyi utwite, bakayaherekanya ku ruziga.

Igice cya 2
Abaza ababyeyi ibintu bikunze kugwa nabi ababyeyi baba baraganiriyeho bagitangira mu isuzuma rya mbere. Agahera aho abafasha gusobanukirwa itandukaniro riri hagati yo kugubwa nabi bidafite icyo bitwaye n'ibimenyetso mpuruza.

Arababaza ati: « Hari uwa kwigisha abandi ku bibazo biba ku mubyeyi utwite biri ku ishusho mfashanyigisho afite mu ntoki? ». Abasaba gufashanya cyangwa umubyeyi umwe akagurana n'undi ikarita niba babishaka. Ashishikariza ababyeyi kwigishanya kuri buri kimenyetso, icyo kimenyetso icyo ari cyo, icyo gishatse gusobanura n'icyo bagikoraho. Arareka ababyeyi bagatanga ibitekerezo. Iyo ikiganiro gisa n'icyacogoye, abaza ikindi kibazo kugira ngo barusheho gusobanukirwa neza.

Igice cya 3
Umuyobozi w'ikiganiro ashimangira ko ibinini bya feri na aside folike bifatwa buri munsi mu rwego rwo gusigasira ubuzima bw'umubyeyi utwite ndetse n'umwana.

Igikorwa gisoza: Kumenyana n'abandi

Igihe: Igikorwa kimara iminota 5

Intego z'iki gikorwa

- Gushimangira ko ababyeyi barushaho kunga ubumwe kandi bagafashanya;
- Kwibutsa ababyeyi ko bazagaruka mu rindi suzuma hamwe n'abo bari bari kumwe mu itsinda nyuma y'ibyumweru 8;
- Abagize itsinda bemeza niba abagabo babo cyangwa abandi bafitanye isano ya bugufi bazatumirwa mu isuzuma ry'ubutaha;
- Kwibutsa ababyeyi kwitabira ubwisungane mu kwivuza.

Imyiteguro y'uyobora ibiganiro

- Gusoma no kumva neza ibikubiye muri iki kiganiro;
- Gutegura igipupe n'umupira ubanze mu budodo.

Ibikoresho/ibyifashishwa

- Gutegura igipupe n'umupira ubanze mu budodo.

Igice cya 1: Guhuriza hamwe

Uyobora ibiganiro afata mu ntoki umupira ubangishije ubudodo akabaza ikintu cy'ingenzi yungukiye mubyaganiriweho mu itsinda ry'uwo munsi n'uburyo bazakifashisha kugira ngo bagire ubuzima bwiza. Noneho agafata umupira ku kadodo kanyuma akagakomeza ntakarekure. Akanagira umupira undi muntu mu itsinda, abanje ku mureba mu maso kugira ngo arebe ko yiteguye kuwufata. Uruhererekane rugenda neza cyane iyo buri wese umupira umugeraho nawe agahita awunagira mugenzi we vuba vuba. Ashobora gutangira awunagira uwo bafatanyije mu kuyobora ibiganiro niba ahagaze aho bateganye, kugira ngo n'ababyeyi babarebereho uko bikorwa.

Igice cya 2

Uyoboye ibiganiro asaba umuntu ufashe wa mupira kuvuga ikintu yungutseho ubumenyi yazigisha undi muntu akamenya amakuru y'ukuri ajyanye n'ubuzima bw'umubyeyi utwite kimwe n'ajyanye no kubyara neza. Yarangiza nawe akawunagira undi muntu uri ku ruziga. Buri wese mbere yo kunagira umupira mugenzi we agenda afata akadodo ko ku mpera y'umupira akagafata mu ntoki agakomeje ntikamucike. Buri muntu agenda afata akanya ko gutanga igitekerezo iyo umupira umugezeho ubundi akawunagira mugenzi we kugeza bose barangije.

Igice cya 3

Uyoboye ikiganiro asaba ababyeyi kugarura wa mupira igihe wahetuye ababyeyi bose; akababwira ko bafite inshingano zo kwigisha abandi ibyiza bigiye mu itsinda ry'uwo munsi. Ababwira kandi ko gusangira ubumenyi birushaho kubafasha kunga ubumwe no gufashanya.

Igice cya 4

Umuyoboziw'ikiganiro ashyira igipupe kuri ka kantu kameze nk'agatanda gakozwe n'urusobe rwa bwa budodo ababyeyi bagiye basigarana mu ntoki igihe batereranaga umupira. Igipupe agishyigikira neza kugira ngo kitagwa. Hanyuma akababaza ati: « Muratekereza iki kuri aka gatanda twakoze? ». Iyo nta muntu usubije, ababanzirize maze abasobanurire ukuntu iyo bafatanije barushaho kugira ingufu mu kwiyitaho ubwabo, kwita kuri bagenzi babo ndetse no kwita ku bana babo.

Igice cya 5: Kwibukiranya

Umuyobozi w'ikiganiro akura cya gipupe kuri ka gatanda; akabwira ababyeyi kumanura utudodo bari bafashe mu ntoki bakadushyira hasi imbere y'ibirenge byabo ariko bakabikorana ubwitonzi ku buryo bwa budodo bugize agatanda budasobana. Mu gihe bamanura utwo tudodo, bibutswa ko bazongera bagahura nyuma y'ibyumweru 8 baje kwisuzumisha kugira ngo baganire ku ngingo z'ingirakamaro zigendanye no gutwita. Basonanurirwa ko isuzuma rizaba risa n'iry'uwo munsi: bazisuzumisha, bavurwe kandi baganire. Abashishikariza kandi kuzitabira bose nk'uko batangiranye itsinda.

Yibutsa ababyeyi itariki n'isaha itsinda rizongera guhuriraho; akabibutsa ko bagomba kubika neza ikarita yo kwisuzumishirizaho inda kandi bazayizana mu isuzuma ry'ubutaha. Ababwira ko haramutse hagize urwara, ashobora kuza igihe icyo ari cyo cyose, ariko igihe ari bazima bagomba gutegereza isuzuma ry'ubutaha ku itariki itsinda ryabo rizongera guhura. Ubwa nyuma, yibutsa ababyeyi kwitabira ubwisungane mu kwivuza, kurara mu nzitiramibu no kurya indyo yuzuye

Igice cya nyuma

Umuyoobozi w'ikiganiro abaza niba hari ukeneye ubufasha bwihariye, nko gusuzumirwa ahiherereye, gufatwa amaraso cyangwa guterwa urushinge; akamubwira icyo yakora. Hari igihe byaba ngombwa ko asigara kugira ngo yitabweho wenyine. Mu gihe ababyeyi bose bamaze kugenda, abayoboye isuzuma n'ikiganiro buzuza igitabo cyajyenewe ibijyanye no kwita ku babyeyi batwite no kubagira inama.

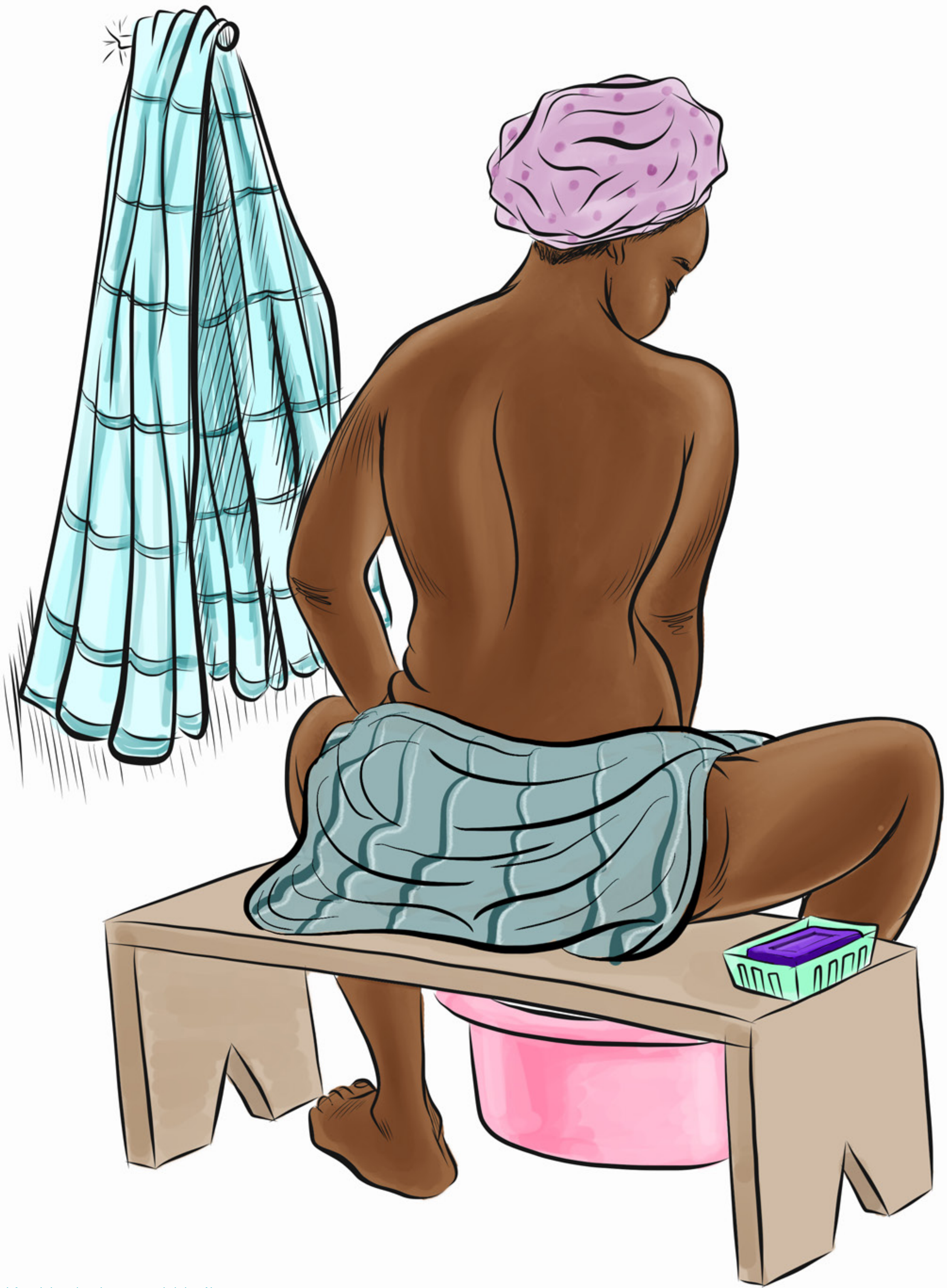

Kugirira isuku umubiri n’imyanya;
Ndangagitsina, woga isabune
n’amazimeza

Amashusho yatunganijwe muri
gahunda ya Ibaruke Neza Mubyeyi

Kwita ku babyeyi batwite no kubaha inama mu itsinda ku nshuro ya kane

Inda y'ibyumweru 36 kugeza ku byumweru 40

Intego y'iki kiganiro

- Gusuzuma uko ubuzima bw'ababyeyi buhagaze;
- Kwitegura ibise no kubyara ndetse no kwandikisha umwana mu bitabo by'iranga mimerere;
- Kwita ku ruhinja no kurera;
- Ibimenyetso mpuruza ku mwana;
- Konsa;
- Kuringaniza no kuboneza urubyaro;
- Ibimenyetso mpuruza ku mubyeyi utwite no ku wamaze kubyara;
- Ikiganiro cyo gusoza no gushishikariza ababyeyi kuzagaruka kwisuzumisha nyuma yo kubyara.

Ibikoresho bikenerwa muri iri suzuma

Igikoresho gipimaumuvuduko w'amaraso;

- Umunzani upima abantu bakuru;
- Ikaramu na dosiye z'ubuvuzi;
- Agakoresho gapima uko umutima w'umwana utera mu nda (foetoscope);
- Agakoreshagapima umuzenguruko w'ikizigira cy'ukuboko (MUAC);
- Igipimo cy'ubushyuhe (Thermometre);
- Ikaramu na dosiye z'ubuvuzi;
- Isaha yagenewe kubara igihe (Horloge), si ngombwa cyane.

Imiti n'ibindi wakwiyegereza

- Ijage nini cyane y'amazi meza yo kunywa n'udukombe twa palasitiki dukwiriye abagore bose bari buze
- Ibinini by'inzoka bya Mebendazole mg 500
- Ibinini bya feri, bigabanyije mu dupaki tugenewe kamwe kuri buri muntu, karimo ibinini 30 cyangwa 60;
- Inkingo za tetanosi.

Imfashanyigisho

- Igipupe;
- Amashusho mfashanyigisho y'ibi bikurikira:
  - » Ay'ibimenyetso mpuruza ku mubyeyi utwite;
  - » Amashusho agaragaza uko umubyeyi umaze kubyara yitabwaho n'ibimenyetso mpuruza ku mubyeyi nyuma yo kubyara;
  - » Amashusho agaragaza uko bita ku ruhinja n'ibimenyetso mpuruza.

Isuzuma ry'ibanze ku babyeyi

Igihe: Iki gikorwa kimara iminota 30

1. Umuyobozi w'ikiganiro asaba ababyeyi bitabiriye isuzuma gukora uyu mukoro mu gihe bategereje gusuzumwa.

- Arababwira ati: « Mu gihe mugitegereje gusuzumwa n'umuforomo/umubyaza, murebe bagenzi banyu mwegeranye muganire ku bijya bibahangayikisha. Muganire uko mubyifatamo iyo muhuye nabyo igihe mutwite. Nimusanga abandi babyeyi mu itsinda nabo bajya bahura n'ibibazo nk'ibyo, mubabaze muti: « Mbese mubyitwayemo mute? ».

2. Akora isuzuma ryuzuye yongereho no gusuzuma neza inda kugira ngo amenye uko umwana ahagaze mu nda (Maneoevre de Leopold).

Gutangiza ibiganiro

Igihe: Iki gikorwa kimara iminota 10

Igice cya 1: Amabwiriza agenga itsinda

Umuyobozi w'ikiganiro abaza ababyeyi niba bibuka amabwiriza bashyiriyeho itsinda ryabo igihe bahuraga bwa mbere. Ababyeyi bagombye gutanga ibisubizo bakanasubiramo amabwiriza agenga itsinda ryabo, akabasaba kubiganiraho.

Iki kiganiro kigamije gusobanura neza uko:

- Ikintu cyose cyaganiriwe mu itsinda kigirwa ibanga
- Buri mubyeyi agomba kugira uruhare mu biganiro;
- Buri wese agomba gutega amatwi no kubaha buri mubyeyi.

Igice cya 2: Umukoro-ngiro w'amazina

N'ubwo itsinda ryamaze guhura ku isuzuma ryo mu itsinda ku nshuro ya 2, hari ubwo haba hari abashyitsi (abagabo cyangwa abo bafitanye isano ya bugufi). Uyu mukoro utuma abantu barushaho kugirirana icyizere n'ubusabane. Abagize itsinda bicara ku ruziga barebana. Buri wese atekereza ku kintu akunda gukora agatekereza n'akantu yakora kagaragaza icyo kintu. Urugero, umubyeyi ashobora kuba akunda gusenga, ubwo rero afatanya ibiganza bye nk'usenga. Undi mubyeyi ashobora kuba akunda kumva umwana we akina

mu nda igihe yirambitse aruhuka, akaba rero yakina asa n'uwirambika ngo asinzire.

Uyoboye ikiganiro arabanza akabanyuramo buri wese amubwira igikorwa akunda anakwereka uko yagikina. Buri wese amaze kuvuga icyo yakinnye, noneho ahera ku murongo abagize itsinda bose bakavuga amazina ya buri muntu n'igikorwa yakinnye.

Igice cya 3: Ibibazo n' ibisubizo by'ababyeyi

Uyoboye ikiganiro abaza ababyeyi ibibahangayikishije baganiriyeho mu gihe cy'amasuzuzuma. Iyo asanze ibibahangayikishije biterwa no kutamenya, babiganiraho kugeza basobanukiwe amakuru nyakuri. Arababaza ati: «Ni izihe ngamba twafata zadufasha koroherwa igihe dufite ikintu runaka kiduhangayikishije? ». Abasaba kubiganiraho muri make. Ingamba zishoboka ni izi zikurikira: kuganira n'inshuti ijya ikuba hafi, inshuti ubusanzwe ijya igira ibitekerezo byiza n'ubugwaneza; gusenga, kuririmba, cyangwa se kwirangaza mu bitekerezo ukora ikintu kigushimisha; cyangwa se no gutekereza ibintu byiza bikwerekeyeho, umwana wawe, ndetse n'ejo hazaza hawe.

Uyoboye ikiganiro azirikana ibibazo ababyeyi babajije cya gihe basuzumwaga buri umwe umwe, akabizana mu itsinda ryose kugira ngo arebe niba ababyeyi bazi ibisubizo byabyo. Ariko na none yirinda kujya avuga uwabajije ikibazo runaka igihe yasuzumwaga yihereye. Iyo asanze batabizi abaha amakuru akwiriye.

Igikorwa cya 1: Kujya ku nda no kubyara (n'ibimenyetso mpuruza)

Igihe: iki gikorwa kimara iminota 15

Intego z'iki gikorwa

- Gushishikariza buri mubyeyi kuza aherekejwe n'umuntu ujya umufasha kandi yizeye igihe aje kwa muganga umuntu ushobora kumukorera ubuvugizi;
- Gusubiramo ibijyana n'imyiteguro yo kubyara;
- Gusubiramo ibijyanye n'ibise;
- Gusubiramo icyo wakora mu gihe wabyarira mu rugo bigutunguye;
- Kwibutsa ababyeyi ko ari inshingano zabo kwandikisha umwana wese ukivuka.

Imyiteguro y'uyobora ibiganiro

- Umuyobozi w'ikiganiro asoma kandi akumva neza ibikubiye muri iki kiganiro.

Ibikoresho/ibyifashishwa

- Amashusho mfasha nyigisho yerekana ibikurikira: Ibimenyetso by'uko ari kunda
- Ibikoresho biri mu gaseke kabugenewe: imyiteguro yo kubyara.

Igice cya 1

Uyoboye ikiganiro ahereza ababyeyi ya mashusho ariho ibimenyetso by'umugore uri ku nda bakayaherekanya. Buri mubyeyi afata ishusho imwe.

Mu gihe bayahererekanya, arababaza ati: «Ni iki uba wizeye gukorerwa mu rwego rwo kwitabwaho igihe uje kubyarira kwa muganga? ». Abasaba kuganira ku cyizere, impungenge n'ibibazo ababyeyi baba bafite ku byerekeranye n'uko bafatwa kwa muganga. Niba ari ngombwa arongera akababaza ati: « Ni gute ababyeyi babyara babigenza kugira ngo bitabweho neza? ». Abasaba kuganira ku kuba baherekezwa n'abagabo babo cyangwa abandi bafitanye isano ya bugufi bashobora kubavugira, kuvuga beruye icyo bashaka n'icyo bakeneye, no kuba baganiriza inshuti n'abagize umuryango ibyerekeye imyiteguro yo kubyara.

Igice cya 2

Umuyobozi w'ikiganiro asaba buri mubyeyi kwerekana ishusho yafashe iriho ibimenyetso by'umugore uri ku nda bakayiganiraho mu itsinda. Ababyeyi bagomba gusubiza, yaba ufashe iyo karita cyangwa abandi boi mu itsinda. Uyoboye ibiganiro agenda yongeramo amakuru igihe ababyeyi bo ubwabo batayafite yuzuye; iyo birangiye yegeranya ya mashusho akayabika.

Igice cya 3

Umuyobozi w'ikiganiro abaza ababyeyi ati: « Ubu se ni ibiki mwakoze mwitegura kubyara? ». Abasaba kuganira ku ngingo z'ingenzi zirebana no kwitegura kubyarira kwa muganga, bagasubiramo amakuru y'ingenzi ku myiteguro yo kubyara yaganiriweho mu gihe cyo kwisuzumisha inda mu itsinda ku nshuro ya 3. By'umwihariko, yongera kwibutsa ababyeyi ku nshingano zabo zo kwandikisha abana bakivuka, abasobanurira uko bikorwa n'uruhare rw'abagize umuryango mu gikorwa cyo kwandikisha umwana.

Igice cya 4: Ibimenyetso mpuruza ku mugore utwite

Umuyobozi w'ikiganiro abahereza amashusho ariho ibimenyetso mpuruza ku mubyeyi utwite; bakayahererekanya ku ruziga hanyuma bakayaganiraho. Ashishikariza ababyeyi kwigishanya kuri buri kimenyetso mpuruza. Icyo kimenyetso icyo ari cyo, icyo gisobanura n'icyo umubyeyi yakora mu gihe abonye icyo kimenyetso.

Igikorwa cya 2: Kwita ku ruhinja n'ibimenyetso mpuruza

Igihe: Iki gikorwa kimara iminota 15

Intego z'iki gikorwa

- Iki gikorwa kigamije gusubiramo ukamaro wo:
    - » Konsa umwana akivuka kandi ntihagire ikindi kintu icyo ari cyo cyose ahabwa mu mezi atandatu ya mbere (Ibi biraza gusubirwamo ku buryo burambuye mu gikorwa rya 3 uyu munsi).
        - › Mu gihe cy'iminota 30 nyuma yo kubyara n'igihe uruhinja rubishatse nibura nyuma ya buri saha mu masaha 24 ya mbere umwana akivuka;

    - Kurinda umwana w'uruhinja ubukonje;
    - Kugumisha umwana mu gituza cy'umubyeyi imibiri ikoranaho mu masaha 24 ya mbere no kubikora kenshi gashoboka nyuma yaho. Kwita ku mwana mu buryo bwiswe Kangaroo (Mother Care (KMC));
    - Kugumisha uruhinja hafi ya nyina no kutarushyira kure mu buriri bwarwo;
    - Gutegereza nibura amasaha 24 mbere yo kuhagira umwana w'uruhinja bwa mbere;
    - Gukinga amadirishya;
    - Kwambika umwana no kumufubika bihagije; ariko kandi nanone umubyeyi akirinda ko umwana yashyuhirana;
  - Gusukura umukondo w'uruhinja no kuwumutsa; kwirinda kuwushyiraho imiti ya gakondo;
  - Isuku y'ibanze;
  - Kuririmbira uruhinja, kuruganiriza, kurukinisha no kurupfumbata; ibi bizafasha mu mikurire myiza y'ubwonko bw'umwana;
  - Kuryama mu nzitiramibu;
  - Kwitabwaho nyuma yo kubyara;
  - Inkingo umwana ahabwa akivuka, nyuma y' ibyumweru 6 nyuma yo kubyara ndetse na gahunda yagutse y'ikingira rusange;

- Gushishikariza ababyeyi kwihutira kujya kwa muganga igihe umwana yaba agaragaje kimwe muri ibi bikenyetso:
  - Guhumeka nabi cyangwa guhumeka vuba vuba;
  - Kutonka neza;
  - Kugaragaza intege nke ku buryo budasanzwe cyangwa no kuba umwana agaragara nk'udatekanye ku buryo budasanzwe;
  - Uruhu rw'umwana rwahinduye ibara bidasanzwe urugero: gusa n'umuhondo;
  - Umukondo w'umwana usa n'uwatukuye, uhinda umuriro cyangwa uva amaraso;
  - Gushyuha bikabije cyane cyangwa gukonja cyane.

Imyiteguro y'uyobora ibiganiro

- Umuyobozi w'ikiganiro asoma kandi akumva neza ibikubiye mu gikorwa kandi agategura n'imfashanyigisho.

Ibikoresho/ibyifashishwa

- Igipupe;
- Amashusho mfashanyigisho y'ibimenyetso mpuruza ku ruhinja.

Igice cya 1

Ushinzwe ikiganiro asobanura ko muri iki kiganiro bari buganire uko umubyeyi agomba kwita ku ruhinja. Ahereza igipupe abari ku ruhande rwe rumwe, akababaza ati: « Ni iki uyu mwana akeneye cyane kugira ngo akure neza? ». Abaza umubyeyi niba yatanga igitekerezo. Niba ntacyo afite, arababaza ati: «Nta waduha igitekerezo ?».

Ubutumwa bw'ingenzi bwo gusubirwamo ni ubu:

- Konsa uruhinja hakiri kare kandi nta kindi uruha;
- Kumushyira ku ibere mu gihe kitarenze isaha avutse n'igihe uruhinja rubishatse nibura buri saha mu masaha 24 ya mbere umwana akivuka;
- Gufasha uruhinja kugumana ubushyuhe:

  - Kugumisha umwana mu gituza imibiri ikoranaho mu masaha 24 ya mbere no kubikora kenshi gashoboka nyuma yahoo; kwita ku mwana mu buryo bwa Kangaru (KMC);
  - Kugumisha uruhinja hafi ya nyina no kutarushyira kure mu buriri bwarwo;
  - Gutegereza gukarabya uruhinja rukivuka nibura amasaha 24;
  - Gukinga amadirishya;
  - Kwambika umwana imyambaro isukuye no ku murinda imbeho ariko kandi umubyeyi akirinda ko umwana ashyuhirana cyane.

- Gusukura umukondo w'uruhinja no kuwumutsa; kwirinda kuwushyiraho imiti ya gakondo;
- Isuku y'ibanze;
- Kuririmbira uruhinja, kuruganiriza, kurukinisha no kurupfumbata —Ibi bifasha mu mikurire myiza y'umwana;
- Kuryama mu nzitiramibu.

Igice cya 2

Umuyobozi w'ikiganiro arababaza ati: « Ni ryari umwana agomba kujyanwa ku kigo nderabuzima kugirango asuzumwe n'umuforomo cyangwa umubyaza? ». Abaza ababyeyi basanzwe bafite abana ati: « Niba warigeze kubyara, ni gute witaweho nyuma yo kubyara? Ibyo ari byo byose hari abasubiza; nibadasubiza arababaza iki kibazo: « Ni nk'ibihe bibazo wari ufite mu minsi ya mbere umaze kubyara? ».

Umuyobozi w'ikiganiro abasobanurira ko ubufasha bahabwa nyuma yo kubyara bushobora kubafasha gusubiza ibyo bibazo. Ababaza ibyo umubyeyi akorerwa nyuma yo kubyara biboneka n'akamaro bimufitiye ndetse n'umwana. Umujyanama w'ubuzima asobanura birambuye uko umwana na nyina bakurikiranirwa mu rugo, aho basurwa n'umujyanama w'ubuzima nyuma y'iminsi 3 n'iminsi 7 ikurikira ivuka ry'umwana.

Igice cya 3
Uyoboye ikiganiro abaza umubyeyi ufashe igipupe ati: « Uratekereza ko uru ruhinja rwumva cyangwa rusobanukiwe icyo mama warwo arimo gukora? ». Yongera kubaza agira ati: « Ni gute ibyo umubyeyi akora bishobora kugira ingaruka ku mwana? ». Abashishikariza kuganira ku kamaro ko kuganiriza no gukinisha umwana kandi ko no kuririmbira umwana ntako bisa! Ababwira ko kurebana n'umwana mu maso ari ingenzi kuko ubutumwa bwose umubyeyi aha umwana bumugeraho bityo ubwonko bwe bugakura neza kandi akazakura ari umwana ufite ubwenge. Umuyobozi w'ikiganiro asaba ufite igipupe kuba agishyize ku ruhande.

Igice cya 4
Umuyobozi w'ikiganiro abahereza amashusho mfashanyigisho ariho ibimenyetso mpuruza ku ruhinja. Asaba buri mubyeyi gufata ishusho imwe hanyuma agahereza mugenzi we, nawe akagenda afata imwe imwe uko bicaye ku ruziga. Buri mubyeyi, umwe umwe, agenda abwira abandi ikibazo kiri ku ishusho gishobora kugaragara ku mpinja. Abandi babyeyi ku ruziga bashobora nabo kugira icyo bongeraho. Uyoboye ikiganiro agomba gukora ku buryo ingingo zikurikira zivugwaho ku bimenyetso bikomeye by'uburwayi:

- Ubuzima bw'uruhinja bumeze bute?
- Ni izihe ngorane umwana w'uruhinja ashobora kugira mu gihe ikibazo gikomeye afite kidakemuwe?
- Ababyeyi b'uwo mwana bakwiye kumukorera iki? (kujya ku kigo nderabuzima).

Ikiganiro kirangiye umuyobozi w'ikiganiro asubirana ya mashusho mfashanyigisho.

Igice cya 5
Uyoboye ikiganiro arabaza ati: « Ni ibihe bibazo impinja zikunze kugira bikaba bidakomeye cyane? Ni ubuhe buryo bwiza bushoboka bwo gufasha izo mpinja ngo zoroherwe? ». Asaba abagize itsinda kuganira ku bibazo bikunze kubaho nko kwituma impatwe, gusa n'uwababutse ku ijosi, cyangwa kuzana uduheri. Ababwira ko ari bibi rwose gukoresha imiti ya gakondo, guca imyotso, kurasaga, cyangwa gukura ibyinyo no guca ibirimi. Abashishikariza kujya bajyana impinja kwa muganga igihe babona ko hari ibintu biteye impungenge bigaragara ku mwana.

Igikorwa cya 3: Konsa

Igihe: Iki gikorwa kimara iminota 15

Intego z'iki gikorwa

- Gushishikariza ababyeyi kujya bashyira impinja mu gituza imibiri ikoranaho kugira ngo zigumane ubushyuhe kandi akababwira kuzashyira uruhinja ku ibere bakimara kubyara nibura mu gihe kitarenze isaha imwe uruhinja rukivuka;
- Kubashishikariza kujya bonsa impinja nta kindi bazihaye mu gihe cy'amezi 6 abanza;
- Kuganira ku buryo bwiza bushoboka bwo gufata umwana no kumuha ibere;
- Kuganira ku myumvire abantu bagira itari yo ku byerekeye konsa;
- Kubabwira kwirinda gutangira guha abana imfashabere y'ibyo kurya cyangwa kunywa bataruzuza amezi atandatu;
- Kubaganiriza uko umubyeyi yakwifata iyo aribwa amabere yonsa;
- Kubashishikariza ko umubyeyi yajya afata amafunguro akungahaye ku ntungamubiri kandi kenshi ariko mu buryo buboneye, ntarenze urugero;
- Kubashishikariza kujya bakomeza gufata ibinini bya feri na aside folike kugeza uruhinja rwujuje amezi 3.

Imyiteguro y'uyobora ibiganiro

- Umuyobozi asoma kandi akumva neza ibikubiye mu kiganiro.

Imfashanyigisho

- Igipupe.

Igice cya 1
Umuyobozi w'ikiganiro abaha icyo gipupe kikabazengurukemo; asaba buri mubyeyi ugiherejwe kuvuga ikintu kimwe yumvise bavuga ku bijyanye no konsa, cyangwa se akigisha bagenzi be konsa, akabibereka akoresheje icyo gipupe. Iyo umubyeyi avuze akantu gato cyane, abaza itsinda ikibazo cyo kubatera umwete ngo batange urugero, abikora agira ati: «Mwese ibyo mwabyumviseho iki? ».

Niba umubyeyi avuze ikintu kitari cyo ku bijyanye no konsa, asaba abandi babyeyi mu itsinda kugira icyo bongeraho, bagatanga ibitekerezo cyangwa bakavuga uko bumva icyo gitekerezo atanze igihe yumvise ko atari cyo. Niba nta muntu n'umwe mu itsinda ukosoye iryo kosa kandi akaba ari ingingo ya ngombwa kuvugaho, umuyobozi w'ikiganiro niwe ugomba gutanga amakuru nyayo.

Igice cya 2
Niba nta makuru y'ibihuha abantu bakunze kuvuga bijyanye no konsa yagaragaye, uyoboye ikiganiro ashobora kubaza ati: « Mwaba mwarigeze kumva bavuga ko . . . ». Ashobora kubaza se ati:« Muratekereza iki kuri ibi bivugwa? ». Ashobora se kubaza ati: « Umukecuru cyangwa umubyeyi ukiri muto akubajije atyo wamusubiza iki, wamukosora ute? ».

Ayo makuru y"ibihuha ashobora kuba amwe muri aya:

- Konsa buri gihe birababaza;
- Mu minsi ibiri ukimara kubyara nta mashereka uba ufite, umwana agomba guhabwa ibindi bintu byo kunywa;
- Ibere ryanjye rishobora kuba ryumagaye, nta mashereka ahagije yabonekamo;
- Umubyeyi urwaye ntakwiye konsa umwana we;
- Iyo umwana arira ntabwo aba yakunze amashere-

ka cyangwa se iyo umwana arutse aba ari ukubera amashereka;

- Si byiza gushyira uruhinja ku ibere rukimara kuvuka ahubwo ugomba gutegereza amashereka akuzura mu mabere.

Igice cya 3

Umuyobozi w'ikiganiro asaba ababyeyi kuganira ku gutangira konsa hakiri kare n'imyumvire itariyo abantu bakunze kugira ku bijyanye no kugira amashereka umuntu akimara kubyara. Arababaza ati: « Ni ryari umwana yagombye gutangira konka nyuma yo kuvuka? ». Ashishikariza ababyeyi konsa umwana mu isaha ya mbere bakibyara. Agashimangira ko uruhinja rugomba guhabwa ibere kenshi ku munsi wa mbere rwavutseho kugira ngo umubiri w'umubyeyi ubashe kuyobora amashereka menshi. Arongera akababaza ati: « Ni gute wamenya ko umwana wawe yonka amashereka ahagije? ». Akabasaba kubiganiraho.

Igice cya 4

Umuyobozi w'ikiganiro abaza ababyeyi niba muri bo hari uzi uko konsa bikorwa. Abaza umwe muri bo kubyerekana akoresheje igipupe. Ababaza ibibazo kugira ngo bose barusheho gusobanukirwa. Ashobora kubaza ati: « Umubyeyi uri konsa aba agomba gufata uruhinja ate? ». Akongera akabaza ati: « We yifata ate? ». Hanyuma agasoza abaza: « Ni iki kikwereka ko uruhinja rutamiye ibere neza? ».

Igikorwa cya 4: Kwita ku mubyeyi nyuma yo kubyara n'ibimenyetso mpuruza ku mubyeyi

Igihe: Iki gikorwa kimara iminota 15

Intego z'iki gikorwa

- Gusubiramo umumaro wo:
  - » Guhorana isuku ku mubiri;
  - » Konsa uruhinja rwawe buri munsi inshuro nyinshi zishoboka (nibura inshuro 10 ku munsi);
  - » Kurya nibura inshuro enye ku munsi no kunywa ibintu bihagije;
  - » Gufata ibinini bya feri na aside folike buri munsi;
  - » Kuryama mu nzitiramibu hamwe n'uruhinja rwawe;
  - » Kujya kwisuzumisha nyuma yo kubyara no gusubira kwisuzumisha nyuma y'ibyumweru 6 ubyaye;
  - » Guteganya igihe uzamara mbere yo kongera gusama, nibura imyaka ibiri;
  - » Guhitamo uburyo bwo kuboneza urubyaro mbere yo kubyara umwana utwite, nk'uburyo bwo kwifashisha ukimara kubyara (PPFP);
  - » Inyunganizi n'ubufasha bw'abavandimwe n'inshuti;
- Ashishikariza umubyeyi kwihutira kujya kwa muganga mu gihe agaragaweho n'ibimenyetso bikurikira:
  - » Kuva bikabije;
  - » Umutwe udakira;
  - » Ububabare bukomeye aho ari ho hose mu mubiri;
  - » Kugagara;
  - » Umuriro cyangwa kumva urembye;
  - » Amabere yahishije cyangwa ahinda umuriro;
  - » Kwihagarika bigoranye cyangwa ubababara;
  - » Kugira ikibazo cyo kujojoba;
  - » Kwiheba bikabije cyangwa guhungabana mu mitekerereze.

Imyiteguro y'uyobora ibiganiro

- Umuyobozi w'ibiganiro asoma kandi akumva neza ibikubiye mu kiganiro no kwegeranya imfashanyigisho ari bugikoreshemo.

Imfashanyigisho

- Amashusho mfashanyigisho akurikira:
  - » Amashusho y'ababyeyi bafite ubuzima bwiza nyuma yo kubyara;
  - » Amashusho y'ibimenyetso mpuruza ku mubyeyi nyuma yo kubyara.
- Ingero z'uburyo bunyuranye bwo kuboneza urubyaro bwashyizwe mu gaseke yateguye.

Igice cya 1

Umuyobozi w'ikiganiro asaba ababyeyi bose guhagarara ku ruziga; abahereza amashusho mfashanyigisho yose icyarimwe. Asaba buri mubyeyi kureba ku ikarita ye akavuga niba igaragaza umubyeyi ufite ubuzima bwiza nyuma yo kubyara cyangwa umubyeyi ufite ikibazo cy'ubuzima gikomeye.

Asaba ababyeyi bafite amakarita kugaragaza ababyeyi bafite ubuzima bwiza kujya iburyo bwe, naho abafite ay'abafite ibimenyetso mpuruza bakajya ibumoso bwe. Ababyeyi bagomba gufashanya niba harimo abatazi neza aho baherereye. Bahindura aho bari bahagaze mu ruziga akabashyira mu bice bibiri ari byo: icy'abafite ubuzima bwiza n'icy'abafite ibimenyetso mpuruza; hanyuma akabasaba kongera kwicara.

Igice cya 2

Uyoboye ikiganiro ahera ku gice cy'ababyeyi bafite amakarita y'abafite ubuzima bwiza ku ruziga; agasaba buri mubyeyi kugenda asobanura ikarita afite. Ashimangira uko umubyeyi agomba kwitwara kugira ngo agire ubuzima bwiza. Iyo bageze ku bijyanye n'isuku y'umuntu ku giti cye, ababaza ibibazo bikurikira kugira ngo barusheho gusobanukirwa:

- Ibijyanye no gukaraba kenshi nyuma yo kubyara mubitekerezaho iki? Abasaba kuganira ku mico gakondo, imigenzereze n'imyemerere ku bijyanye no gukaraba. Abashishikariza koza mu myanya myibarukiro kenshi n'amazi meza;

- Iyo batagize icyo bavuga ku mibonano mpuzabitsina ababazaho ibibazo. Ababaza agira ati: « Mutekereza iki ku mibonano mpuzabitsina, ishingiye ku muco nyarwanda, ikorwa ku munsi wo kwita umwana izina? ». Arongera akabaza ati: « Ese umuntu aba yihuse cyane iyo yongeye gukora imibonano mpuzabitsina nyuma yo kubyara? ». Abasaba kubiganiraho.

Nibavuga ku bijyanye no kuboneza urubyaro, arababza ati: « Ni uwuhe mubare w'imyaka ukwiye wategereza ngo ubone gukurikiza umwana? ». Igisubizo ni imyaka 2 kugeza kuri 3. Arongera akabaza ati: « Ni ubuhe buryo bwo kuboneza urubyaro ababyeyi bashobora guhita bahabwa igihe babyariye kwa muganga? ». Abasaba kuganira ku buryo bunyuranye bwo kuboneza urubyaro bukoreshwa umubyeyi akimara kubyara.

Igice cya 3

Noneho, ababyeyi bafite amakarita ariho ibimenyetso mpuruza barayasobanura. Uyobora ikiganiro akabaza umwe mu bagize itsinda ati: « Wabigenza ute ibyo biramutse bikubayeho? ». Igisubizo kigomba ni ukujya kwa muganga. Arongera akabaza ati: « Ibi biramutse bikubayeho ariko ukaba ufite intege nke ntacyo ubashije kwikorera wabigenza ute? ». Yongera kubaza ati: « Ni nde wagufasha?». Ese uwo muntu wagufasha azi igihe agomba kukujyanira kwa muganga? Umuyobozi ashimangira ko ababyeyi bashobora kwigisha abagabo babo cyangwa abo bafitanye isano ya bugufi ibyerekeranye n'ibimenyetso mpuruza.

Igikorwa gisoza: Ubusabane no kumenyana

Igihe: Iki gikorwa kimara iminota 5

Intego z'iki gikorwa

- Gushimangira ko ababyeyi bunga ubumwe kandi bagafashanya;
- Kwibutsa ababyeyi ko bazagaruka mu rindi suzuma hamwe n'abo bari bari kumwe muri iri, nyuma y'ibyumweru 8;
- Abagize itsinda bemeza niba abagabo babo cyangwa abandi bafitanye isano ya bugufi bazatumirwa mu isuzuma ry'ubutaha;
- Kwibutsa ababyeyi kwitabira ubwisungane mu kwivuza.

Imyiteguro y'uyobora ibiganiro

- Uyobora ibiganiro asoma kandi akumva neza ibikubiye mu kiganiro;
- Ategura igipupe n'umupira ubanze mu budodo.

Imfashanyigisho

- Igipupe;
- Umupira ubanze mu budodo.

Igice cya 1: Guhuriza hamwe

Uyobora ikiganiro afata mu ntoki umupira ubangishije ubudodo akabaza ikintu cy'ingenzi yungukiye mubyaganiriweho mu itsinda ry'uwo munsi n'uburyo bazakifashisha kugira ngo bagire ubuzima bwiza. Noneho agafata umupira ku kadodo kanyuma akagakomeza ntakarekure. Akanagira umupira undi muntu mu itsinda, abanje kumureba mu maso kugira ngo arebe ko yiteguye kuwufata. Uruhererekane rugenda neza cyane iyo buri wese umupira umugeraho nawe agahita awunagira mugenzi we vuba vuba; ashobora gutangira awunagira uwo bafatanyije mu kuyobora ikiganiro niba ahagaze aho bateganye, kugira ngo n'ababyeyi barebereho uko bikorwa.

Igice cya 2

Uyoboye ikiganiro asaba umubyeyi ufashe wa mupira kuvuga ikintu yungutseho ubumenyi yazigisha undi muntu akamenya amakuru y'ukuri. Yarangiza nawe akawunagira undi muntu ku ruziga; buri wese mbere yo kuwunagira mugenzi we agenda afata mu ntoki akadodo ko ku mpera y'umupira akagakomeza kugira ngo katamucika. Buri muntu agenda afata akanya ko gutanga igitekerezo iyo umupira umugezeho ubundi akawunagira mugenzi we kugeza bose barangije.

Igice cya 3

Umuyobozi w'ikiganiro asaba ko bamugarurira wa mupira umaze guhetura ababyeyi bose; akababwira ko bafite inshingano zo kwigisha abandi ibyiza bigiye mu itsinda ry'uwo munsi. Yongera kubibutsa ko gusangira ubumenyi birushaho kubafasha kunga ubumwe no gufashanya.

Igice cya 4

Umuyobozi w'ikiganiro ashyira igipupe kuri ka kantu kameze nk'agatanda gakozwe n'urusobe rwa bwa budodo ababyeyi bagiye basigarana mu ntoki igihe batereranaga umupira. Igipupe aragishyigikira neza kitagwa kugira ngo kitagwa. Hanyuma agasubiramo inyigisho zose z'uwo munsi.

Uyoboye ikiganiro akomeza afata cya gipupe akagishyira kuri ka gatanda, agasaba ababyeyi bose kumanura atudodo bari bafite mu ntoki bakadushyira hasi imbere yabo, ariko bakabikorana ubwitonzi kugira ngo bwa budodo budasobana. Mu gihe bamanura twa tudodo abibutsa ko bazongera guhurira mu itsinda nyuma y'ibyumweru 8 kugira ngo bahabwe ubufasha n'inama z'umubyeyi umaze igihe gito abyaye. Abasobanurira ko ibizaganirwaho ari ibyerekeye guhabwa inkingo, kimwe n'izindi ngingo zirebana n'umubyeyi umaze kwibaruka. Abasobanurira kandi ko ibizakorwa mu itsinda rikurikira bisa n'ibyakozwe uwo munsi; harimo: isuzuma, guhabwa ubuvuzi no kungurana ibitekerezo. Abasaba kandi ko buri wese agomba kuzitabira, ntawe ugomba gusiba, kugira ngo abonane na bagenzi.

Igice gisoza

Niba harimo ukeneye ubufasha bwihariye; urugero hari nk'uwifuza gusuzumirwa ahihereye, gufatwa amaraso cyangwa guterwa urushinge, amubwira icyo yakora. Hari igihe byaba ngombwa ko asigara kugira ngo yitabweho wenyine. Ababyeyi bose bamaze gutaha, umuyobozi yuzuza mu gitabo cy'ubuzimab w'umubyeyi utwite.

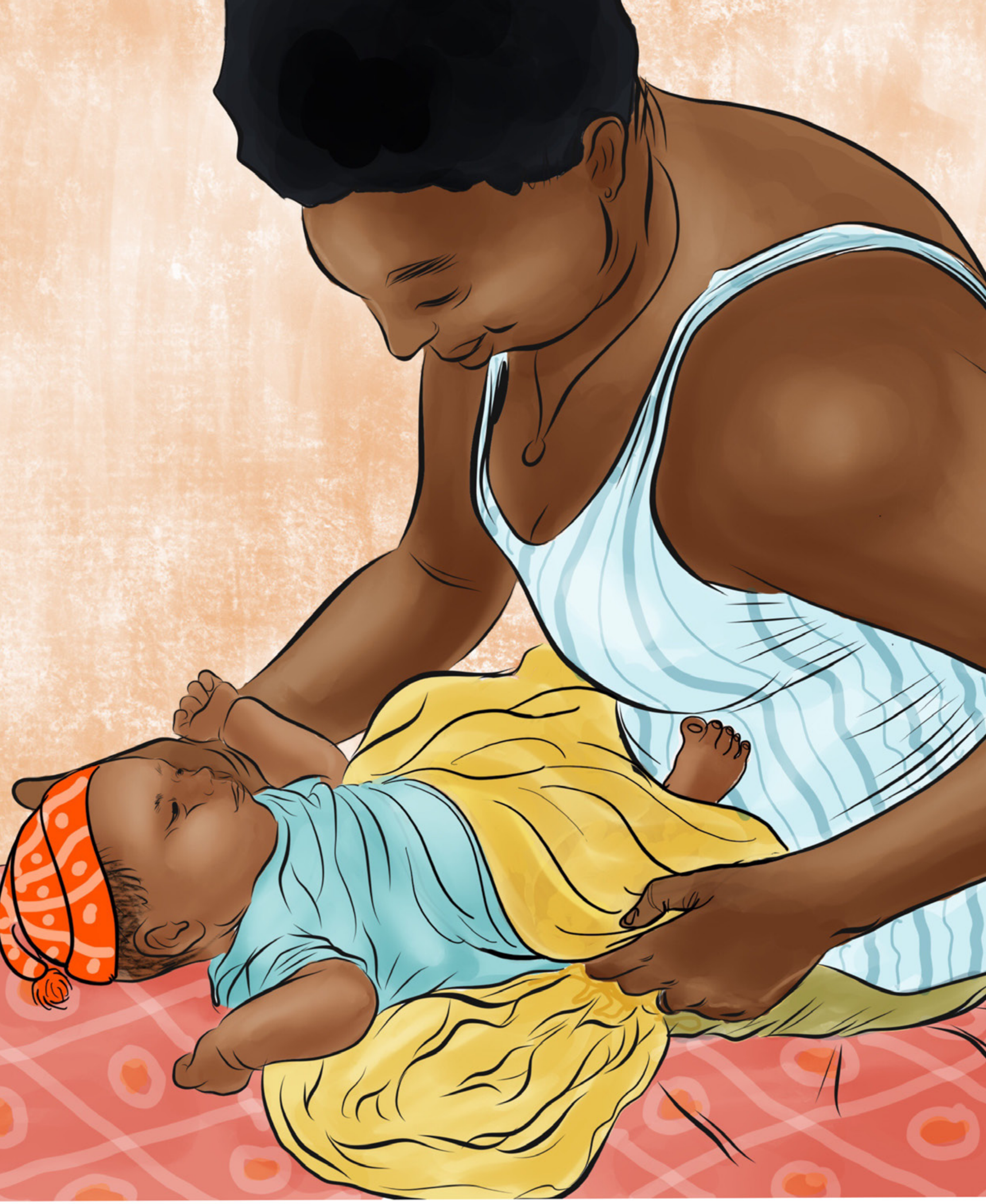

Kwambika uruhinja imyenda ikwiriye no kurworosa—ntirushyuhirane cyangwa ngorukonje cyane

Amashusho yatunganijwe muri gahunda ya Ibaruke Neza Mubyeyi

Kwita ku babyeyi no kubagira inama nyuma yo kubyara mu itsinda

Igihe: Iki gikorwa kimara ibyumweru 6 nyuma yo kubyara

Intego z'iki kiganiro

- Gusuzuma ubuzima bw'umwana n'umubyeyi;
- Ababyeyi barasabana kandi bakagenda berekana abana babo mu itsinda;
- Gukoresha inzitiramibu iteyemo umuti;
- Kuboneza urubyaro;
- Kwita ku ruhinja no kurera;
- Konsa;
- Ibimenyetso mpuruza ku ruhinja;
- Ikiganiro cyo gusoza no gusezeranaho.

Igihe gikenewe muri iki kiganiro: amasaha 2

Ibikoreshwa muri iri suzuma

Ibikoresho mu gusuzuma iby'ibanze ku buzima

- Umunzani upima impinja;
- Agakoresho gapima umutima, ibihaha ku mpinja;
- Agashumi gapima umuzenguruko w'ikizigira cy'ukuboko (MUAC);
- Igipimo cy'ubushyuhe;
- Ikaramu na dosiye z'ubuvuz;i
- Isaha yagenewe kubara igihe (Horloge), si ngombwa cyane.

Inkingo ku ruhinja

- Urukingo rw'imbasa;
- Urukingo rwakaniga, umwijima (Hepatite B), agakwega (Tetanos), umusonga (Pinemokoke), n'urukingo rw'impiswi (Rotavirus).
- Inkingo ku babyeyi igihe biteganijwe
- Urukingo rwa Tetanosi.

Imiti n'ibindi wakwiyegereza

- Vitamine A ibinini 200,000 ku babyeyi batahise babihabwa bakimara kubyara; niba hatariho impungengee ko umubyeyi yaba yarongeye agasama.

Imfashanyigisho

- Uburyo bunyuranye bwo kuboneza urubyaro mu gaseke kabugenewe;
- Igipupe;
- Amashusho mfashanyigisho y'ibimenyetso mpuruza ku ruhinja

Gusubiramo amafishi mbere y'isuzuma akoresha amadosiye y'ubuvuzi ari mu gifuniko cyabugenewe:

- Umubyeyi yahawe urushinge rumukingira Tetanosi?
    - » Yaba yarahawe urukingo rwa Tetanosi rwa 1 n'urwa 2?
    - » Yaba akeneye urukingo rwa Tetanosi rwa 3?
    - » Niba atararuhawe, ararwiyegereza kugira ngo abe yiteguye kurumuha itsinda risoje isuzuma. Niba yararuhawe, ategura itariki azagarukira kugira ngo ahabwe urundi rukingo rwa tetanosi, akanamwibutsa kuzubahiriza iyo tariki yo guhabwa urukingo.
- Umubyeyi yaba yarahawe uburyo bwo kuboneza urubyaro butangwa umuntu akimara kubyara? Yahawe ubuhe buryo? Akamubaza niba hari ikibazo afite.
    - » Niba umubyeyi yarifuje kuboneza urubyaro nyuma yo kubyara akaba atarabuhawe, amubaza uburyo yifuza gukoresha akamugira inama zikwiriye;
    - » Niba akeneye uburyo bwo kuboneza urubyaro uwo munsi umubyeyi agomba kubutahana nyuma yo kumusuzumwa no kwihitiramo ibimubereye.
- Umubyeyi yaba afite ingorane zituma akeneye koherezwa ku bitaro nyuma yo kubyara?

Amasuzuma y'ibanze ku babyeyi

Igihe: Iki gikorwa kimara iminota 30

Ababyeyi baruhukira ku ruziga uwo munsi mu gihe bagitegereje, nta bindi bikorwa biteganijwe muri aya masuzuma y'ubuzima kuko bagomba kuba baganira kandi banasabana. Uyoboye ibiganiro akora amasuzuma y'ubuzima yarangiza agasaba umubyeyi kumwereka ikarita yo kwisuzumisha inda. Yifashisha uru rutonde:

Ubuzima bw'umwana

- Haba hari ikibazo umwana yagize kuva avutse?
- Umwana yaba yarasuwe n'umujyanama w'ubuzima nyuma yo gusezererwa kwa muganga?

- Umwana yaba yaravukiye igihe? (Yavutse ku byumweru 37cyangwa nyuma yabyo)?
- Haba hari ikibazo umwana afite gituma ashobora koherezwa ku bitaro bikuru?
- Umwana yigeze ahabwa inkingo?
  - » Akimara kuvuka: igituntu, imbasa;
  - » Umwana yaba yarahawe izi nkingo: akaniga, umwijima, agakwega, impiswi, urukingo rwa 2 rw'imbasa, urw'umusonga?
  - » Niba atarazihawe, zigomba kuba ziteguwe kugira ngo umwana azihabwe, iyo agejeje ku byumweru 6.

Ubuzima bw'umubyeyi

- Hari ibibazo yigeze agira?
- Umubyeyi yaba afite ikibazo cyatuma yoherezwa ku bitaro?
- Hari uburyo umubyeyi yaba yarahawe bwo kuboneza urubyaro? Niba ntabwo, yaba ashaka guhabwa uburyo bwo kuboneza urubyaro uwo munsi?
- Umubyeyi yaba yarahawe Vitamini A?
  - » Niba ntayo yahawe ayimuhe uwo munsi;
  - » Ntayo amuha niba amaze ibyumweru 8 abyaye kandi akaba nta buryo bwo kuboneza urubyaro yakoreshaga; agomba kubanza gusuzuma ko umubyeyi yaba atarongeye gusama.

Gusuzuma uruhinja

- Asuzuma uko umwana amerewe muri rusange;
- Abaza uko umwana yonswa kandi agashishikariza umubyeyi kumwonsa gusa inshuro nyinshi ku munsi nta kindi amuha;
- Yongera gupima kandi agasuzuma ibiro by'uruhinja;
- Asuzuma uko umwana aseka, uko arebana n'umuntu mu maso ndetse n'uko akurikiza amaso umuntu cyangwa ikintu.

Gusuzuma umubyeyi: Mubaze niba hari ibibazo afite

- Abaza niba ajya yumva yihebye mu mitekerereze ye;
- Amubaza niba yarateganyije gukoresha uburyo bwo kuboneza urubyaro;
- Bitewe n'uko yabonye umubyeyi ameze, amusaba kuza gusigara itsinda ritashye kugira ngo ahabwe uburyo bwo kuboneza urubyaro yihitiyemo cyangwa ahabwe gahunda y'igihe azagarukira kubuhabwa.

Ikiganiro kibanza

Igihe: Iki kiganiro kimara iminota 10

Igice cya 1: Amabwiriza agenga itsinda

Abaza ababyeyi niba bibuka amabwiriza bashyiriyeho itsinda ryabo igihe bahuraga bwa mbere. Ababyeyi bagombye gutanga ibisubizo bakanasubiramo amabwiriza agenga itsinda ryabo. Abasaba kubiganiraho iminota 5. Ikigamijwe muri iri suzuma ni ugusobanura neza ko:

- Ikintu cyose cyaganiriwe mu itsinda kigirwa ibanga;
- Buri mubyeyi agomba kugira uruhare mu biganiro;
- Gutega amatwi no kubaha buri mubyeyi.

Igice cya 2: Amakuru y'impinja

Asaba ababyeyi guhagarara ku ruziga bagenda babwira bagenzi babo amazina y'abana babo, bavuga izina n'ikintu umwana yihariye. Icyo kintu gishobora kuba ikintu umwana ajya akora, cyangwa se akavuga ikintu kijyanye n'abavandimwe b'umwana cyangwa undi muntu mubagize umuryango, cyangwa ikintu ashima kuri uwo mwana.

Igice cya 3: Ibibazo n'ibisubizo by'ababyeyi

Uyobora ikiganiro azirikana ibibazo ababyeyi babajije igihe basuzumwaga umwe ku wundi uwo munsi, akabizana mu itsinda ryose areba niba ababyei bazi ibisubizo byabyo. Yasanga batabizi akabaha amakuru akwiriye.

Igikorwa cya 1: Konsa

Igihe: Iki gikorwa kimara iminota 15

Intego z'iki gikorwa

- Gushishikariza ababyeyi konsa impinja nta kindi bazihaye mu gihe cy'amezi 6 abanza;
- Kuganira ku myumvire itari yo abantu bakunze kugira ku bijyanye no kugaburira abana;
- Gukangurira ababyeyi kwirinda ingeso yo gutangira guha abana imfashabere y'ibyo kurya cyangwa kunywa hakiri kare;
- Kuganira ku bibazo ababyeyi bonsa bahura nabyo (urugero kubabara amabere).

Imyiteguro y'uyobora ibiganiro

- Uyobora ikiganiro agomba gusoma no kumva neza ibikubiye mu kiganiro.

Ibikoresho/ibyifashishwa

- Igipupe.

Igice cya 1

Umuyobozi w'ikiganiro arabaza ati: « Hari uwaba afite ikibazo ku bijyanye nokonsa umwana? ». Iyo hari umubyeyi wigeze abaza ikibazo, asaba itsinda kugisubiza niba babishobora. Urugero, ashobora kubabaza ati: « Mwese ibyo mwabyumvise mute? ». Ashobora no kubaza ati: « Hari uwaba afite icyo abiziho muri mwe? ».

Iyo umubyeyi avuze ikintu kitari cyo kubijyanye no konsa, uyoboye abaza abandi niba bagira icyo bongeraho, niba hari ibitekerezo batanga cyangwa bakavuga uko bumva icyo gitekerezo kitari cyo. Areka abagize itsinda bakaba ari bo bakosora iryo kosa. Iyo nta muntu n'umwe mu itsinda ukosoye iryo kosa kandi akaba ari ingingo ya ngombwa kuvugaho, ubwo ni uyoboye ikiganiro ugomba gutanga amakuru nyayo.

Igice cya 2
Iyo nta makuru y'ibihuha abantu bakunze kuvuga bijyanye no konsa yagaragaye, uyoboye ikiganiro abivugaho. Ashobora kubaza ati: « Mwaba mwarigeze kumva bavuga ko . . . ». Ashobora no kubaza ati: « Muratekereza iki kuri ibi bavuga? ». Yabaza kandi ati: « Umukecuru cyangwa umubyeyi ukiri muto akubajije atyo wamusbiza iki? ». Cyangwa ati: « Wamukosora ute? ». Ayo makuru y'ibihuha ashobora kuba amwe muri aya akurikira:

- Konsa buri gihe birababaza;
- Mu minsi ibiri ukimara kubyara nta mashereka uba ufite, umwana agomba guhabwa ibindi bintu byo kunywa;
- Amabere yanjye ashobora kuba yumagaye, nta mashereka ahagije yabonekamo;
- Umubyeyi urwaye ntakwiye konsa umwana we;
- Iyo umwana arira ntabwo aba yakunze amashere ka cyangwa se iyo umwana arutse aba ari ukubera amashereka.

Igice cya 3
Uyoboye ikiganiro arababaza ati: « Kubera iki tugirwa inama yo konsa umwana byonyine nta kindi tumuha mu mezi atandatu abanza? ». Arongera akababaza ati: « Ni ryari umwana akenera imfashabere? ». Akabasaba kuganira ku buryo buboneye bwo guha umwana imfashabere.

Igikorwa cya 2: Umuryango ufite ubuzima bwiza

Igihe: Iki gikorwa kimara iminota 20

Intego z'iki gikorwa

- Gushimira ababyeyi kugaragaza imyitwarire myiza mu kurera abana babo;
- Kuganira ku myitwarire yo gutanga uburere ifasha imikurire ikwiye y'ubwonko bw'umwana, irimo kuririmbira umwana, kumubwira udukuru, kumuganiriza, kumusomera udutabo tw'abana, kumukinisha no kumwereka urukundo. Ibi bikorwa na se na nyina, abavandimwe, abagize umuryango ndetse n'abandi barera umwana;
- Gushishikariza ababyeyi kuryama mu nzitiramibu mu rwego rwo kwirinda malariya
- Gutegura neza gahunda yo kuboneza urubyaro (kongera gusama hashize nibura imyaka ibiri nyuma yo kubyara) n'uburyo bwo kuboneza urubyaro;
- Gushishikariza ababyeyi kugira ubwisungane mu kwivuza ku bagize umuryango bose.

Imyiteguro y'uyobora ibiganiro

- Uyobora ikiganiro asoma kandi akumva neza ibikubiye mu kiganiro yegeranya n'ibikoresho.

Ibikoresho/ibyifashishwa

- Igipupe;
- Ingero z'uburyo bunyuranye bwo kuboneza urubyaro ziri mu gakoresho kateganyijwe;
- Amashusho mfashanyigisho y'ibimenyetso mpuruza ku ruhinja;
- Inzitiramibu.

Igice cya 1
Umuyobozi w'ikiganiro atangiza ikiganiro ku bijyanye n'inzitiramibu iteye umuti akoresheje ibibazo bituma ababyeyi batanga ibisobanuro birambuye. Nk'urugero ashobora kubaza ati: « Hari ababyeyi mwaba muzi baryama mu nzitiramibu iteyemo umuti? ». Ashobora no kubaza ati: « Mwaba muzi ababyeyi bafite abana babo bigeze kurembywa na malariya? ».

Abaza ibibazo bisaba ababyeyi gutanga ibisobanuro bigufi kugira ngo bakomeze kunguka ubumenyi ku nzitiramibu iteye umuti. Nk'urugero, ashobora kubaza ati: «Birakomeye kubona inzitiramibu iteye umuti? ». Ashobora kandi kubaza ati: « Bimerera nabi umuntu kuryama mu nzitiramibu iteye umuti? ». Ashobora no kubaza ati: « Ni gute wayiryamamo kugira ngo bikugwe neza? ». Asaba umubyeyi umwe kwerekana uko bamanika inzitiramibu n'uko bayikoresha.

Igice cya 2
Umuyobozi w'ikiganiro abaza umwe mubagize itsinda ati: « Hari umuntu muri mwe waba azi umubyeyi ufite abana b'indahekana? ». Umuyobozi asaba ko babiganiraho. Arongera akabaza ati: « Ni nyuma y'igihe kingana iki umubyeyi wabyaye yakongera gusama ntibihungabanye ubuzima bwe? ». Arongera akabasaba kuganira ku gutegereza nibura imyaka ibiri mbere yo kongera gusama.

Dore ikindi bibazo yakwifashisha: « Ni gute umubyeyi agena igihe gikwiriye cyo kwongera gusama? ». Ashobora no kubaza ati: « Ni ubuhe buryo bwagufasha kwirinda gusama mu gihe cy'imyaka ibiri? ». Nyuma y'ibyo bibazo abasaba kuganira ku buryo bunyuranye bwo kwirinda gusama. Iyo ababyeyi batabashije kuvuga uburyo bwo kuboneza urubyaro bwose bubaho, uyoboye ikiganiro atambutsa ka gakoresho karimo uburyo bunyuranye bwo kuboneza urubyaro, agasaba ababyeyi kuvuga uko buteye. Ikindi gishoboka, niba ababyeyi hari uburyo runaka bafiteho ikibazo, abufata mu ntoki akabubasobanurira ku buryo burambuye.

Igice cya 3
Umuyobozi abaza ibibazo bikurikira kugira ngo barusheho gusobanukirwa. Ashobora kubaza ati: « Ukiri umwana, hari umuntu wakuririmbiraga, wakuganirizaga cyangwa wagukinishaga? ». Agakomeza agira ati: « Byakugizeho izihe ngaruka? ». Hanyuma akongera akabaza ati: « Mwigeze mubona ababyeyi baririmbira, baganiriza cyangwa bakinisha abana babo? ». Agasoza abaza ati: « Bene abo bana bitwara bate igihe bakuze? ». Nyuma y'ibyo bibazo, asaba abagize itsinda kubiganiraho.

Abaza ababyeyi niba hari akaririmbo bazi ko kuririmbira impinja; hagira ushaka kukababwira akamusaba kukigisha itsinda. Hanyuma itsinda ryose rikakaririmba. Ubasaba kukitoza inshuro runaka niba ari ngombwa; hanyuma agafata cya gipupe cyenda kumera nk'umwana nya mwana, akaberaka uko umubyeyi aririmbira umwana n'uko aba amureba mu maso go amwereke ko ariwe aririmbira.

Iyo nta mubyeyi wemeye kwigisha abandi akaririmbo, uyoboye ikiganiro niwe ukabigisha. Nk'urugero, umuyobozi w'ikiganiro akaririmba: «Tuje kuvuza impundu Uwera, z'umwana wibarutse Mwiza, tuje kubyinira uwo mushyitsi waje atugana…».

Arababaza ati: « Mwari muzi ko kuririmbira umwana bimufasha gukura mu bwonko? ». Ababwira ko ari ibintu bifasha ubwonko bw'umwana gukura no gukora neza n'ubwo umwana aba asa n'utabisobanukiwe; ubwonko bw'umwana buba bukeneye udukorwa dushimishije nko kumva nyina amuririmbira, amuganiriza cyangwa amukinisha. Iyo umwana adakorewe bene utwo tuntu, ntabwo ubwonko bwe bukura neza uko bikwiriye.

Ababwira ko igihe baririmbiye, baganirije kandi bagakinisha abana babo, bibafasha gukura bafite ubwenge bwinshi; iyo ikaba intambwe ya mbere yo kubafasha kugera ku byiza ababyeyi babifuriza mu buzima bwabo.

Igikorwa cya 3: Ibimenyetso mpuriza ku ruhinja

Igihe: Iki gikorwa kimara iminota 15.

Intego z'iki gikorwa

- Uyoboye ikiganiro abasubiriramo akamaro ko kuryama mu nzitiramibu;
- Agashishikariza buri mubyeyi kwihutira kujya kwa muganga mu gihe umwana agaragaje ibimenyetso bikurikira:
  - » Guhumeka nabi cyangwa guhumeka vuba vuba;
  - » Kutonka neza;
  - » Kugira intege nke bidasanzwe cyangwa kubona umwana yabuze amahoro bidasanzwe;
  - » Uruhu rwabaye umuhondo;
  - » Umukondo wahishije, uhinda umuriro cyangwa uva;
  - » Gushyuha bikabije cyane cyangwa gukonja cyane.

Imyiteguro y'uyobora ibiganiro

- Umuyobozi w'ikiganiro asoma kandi akumva neza ibikubiye mu kiganiro.

Ibikoresho/ibyifashishwa

- Amashusho mfashanyigisho y'ibimenyesto mpuruza ku ruhinja/umwana.

Igice cya 1

Uyoboye ikiganiro abahereza amashusho mfashanyigisho ariho ibimenyetso mpuruza ku ruhinja. Akabwira ati: « Buri wese afatemo ishusho imwe ». Amashusho barayaherezanya aho bari ku ruziga, buri wese afatamo imwe. Ababyeyi bagenda bavuga, umwe umwe, ibibazo bigaragara ku ishusho afite; abandi babyeyi bagize itsinda bashobora gusabwa kugira icyo bongeraho.

Buri mubyeyi umwe umwe agende avuga ikibazo kiri ku ishusho gishobora kugaragara ku mpinja. Abandi babyeyi ku ruziga bashobora nabo kugira icyo bongeraho. Uyoboye ikiganiro agomba gukora ku buryo ingingo zikurikira zivugwaho ku bimenyetso bikomeye by'uburwayi:

- Ubuzima bw'uruhinja uumeze bute?
- Ni ibihe bibazo umwana ashobora kugira mu gihe agize uburwayi ubwo ari bwo bwose ntavurwe uko bikwiriye?
- Ababyeyi b'uwo mwana bakwiye kumukorera iki? (kujya kwa muganga).

Nyuma yo kurangiza ikiganiro, ukiyoboye asubirana ya mashusho mfashanyigisho.

Igice cya 2

Umuyobozi w'ikiganiro arabaza ati: « Ni gute umubyeyi ashobora kurinda uruhinja rwe uburwayi? ». Akora ku buryo mu kiganiro ingingo zikurikira zivugwaho, niba zitigeze zivugwaho uwo munsi:

- Kuryama mu nzitiramibu;
- Gukaraba intoki kenshi n'amazi meza n'isabune;
- Konsa umwana nta kindi umuhaye mu mezi atandatu abanza yose;
- Inkingo.

Igice cya 3

Umuyobozi arabaza ati: « Ni ibihe bibazo bikunze kuba ku mpinja nyinshi nyamara zitarwaye mu by'ukuri? ». Arongera akabaza ati: « Ni iki wakora igihe ibyo bibayeho? ». Asaba abagize itsinda kubiganiraho. Akora ku buryo igikunze kubaho cyane kuruta ibindi ari cyo: « icyo mu nda »; kivugwaho, gusa n'uwababutse ku ijosi, kwituma impatwe n'uduheri ku ruhu.

Abasaba kuganira ku bisubizo byiza bishoboka ku bibazo bikunze kubaho; akababwira ko ari bibi rwose gukoresha imiti ya gakondo, guca imyotso, kurasaga, guca ibirimi, gukura ibyinyo no gutangira guha umwana imfashabere hakiri kare.

Igice cya 4

Uyoboye ikiganiro arabaza ati: « Hari ibindi bibazo mwabaza uyu munsi?». Ashishikariza ababyeyi kubaza ibibazo bijyanye no kubyara no kurera, noneho ibyo babajije akabigarura mu itsinda rigashaka ibisubizo. Iyo nta muntu utanze igisubizo, uyoboye itsinda niwe ugisubiza.

Igikorwa cya 4: Inkingo

Igihe: Iki gikorwa kimara iminota 5.

Intego z'iki gikorwa

- Gusobanura inkingo zigiye gutangwa uwo munsi;
- Gushishikariza ababyeyi gukingiza abana inkingo zose uko zateganyijwe muri gahunda y'ikingira mu Rwanda.

Imyiteguro y'uyobora ibiganiro

- Umuyobozi w'ikiganiro asoma kandi akumva neza ibikubiye mu kiganiro kandi agatunganya ibijyanye n'inkingo.

Igice cya 1

Umuyobozi w'ikiganiro aravuga ati: « Uyu munsi dufite inkingo ziri buze guhabwa abana; izo nkingo zirinda abana banyu uburwayi bwinshi kandi bubi cyane ». Avuga ko abo bana bagomba kugaruka gukingirwa igihe bujuje ibyumweru 10 cyangwa amezi abiri n'igice); igihe bujuje ibyumweru 14 cyangwa amezi atatu n'igice; igihe bafite amezi 9 n'igihe bafite amezi 15.

Buri mubyeyi agomba kuba afite ifishi y'ikingira y'umwana; mu gihe cyo gusobanurira umubyeyi andi matariki azagarukiraho gukingiza, uyoboye ikiganiro abyerekana ku ifishi y'ikingira. Hanyuma akababaza niba hari ibibazo baba bafite ku nkingo.

Ikiganiro cyo gusoza: Ibyo ababyeyi bifuza kuzageraho

Igihe: Iki kiganiro kimara iminota iva kuri 5 kugeza ku 10.

Intego z'iki kiganiro

- Ababyeyi baganira ku byiyumvo, amarangamutima n'ubuhamya ku byababayeho;
- Ababyeyi bagenda bagira ibyo bashima kuri bagenzi babo nk'ababyeyi n'inshuti.

Ibikoresho/ibyifashishwa:

- Muri iki kiganiro nta gikoresho uyoboye ikiganiro ateganya.

Igice cya 1

Uyoboye ikiganiro yibutsa ababyeyi kubika no gufata neza ikarita y'ubwisungane mu kwivuza kuko hari ubwo umwana arwara agakenera kuvuzwa.

Igice cya 2

Buri wese uko bari ku ruziga asangiza abandi ubutumwa batahana; bushobora kuba ari ikintu yizeye kuzageraho mu gihe cyizaza kijyanye n'umwana we, ikintu cy'ingenzi yigiye mu itsinda ndetse n'isuzuma cyangwa se ikintu cyamubayeho gikomeye gifuza kubwira abandi.

Igice cya 3

Umuyobozi ashimira ababyeyi kuba bitabiriye isuzuma ry'itsinda no kuba bagize uruhare mu byakozwe mu itsinda. Abwira ababyeyi ko agiye gukingira abana babo (niba gukingira bikorwa nyuma y'isuzuma rikorwa mu itsinda).

Igice cya nyuma

Niba hari umubyeyi ukeneye ubufasha bwa wenyine, kugira ikizamini afatirwa cyangwa guterwa urushinge, amubwira icyo agomba gukora. Ababyeyi bose bamaze gutaha, azirikana kuzuza mu gitabo cyo gukurikirana umubyeyi nyuma yo kubyara.

Nyuma ya buri suzuma ryo mu itsinda, uyoboye ikiganiro:

- Asaba ababyeyi mu kinyabupfura kuva aho itsinda ryari ryahuriye;
- Areba niba hari ibintu bikigomba gukorwa ku mubyeyi n'uruhinja;
- Abayoboye ibiganiro bombi bafatanya mu kuzuza ifishi yo kureba uko isuzuma ryagenze;
- Buzuza inyandiko zijyana no kwisuzumisha inda cyangwa kwisuzumisha nyuma yo kubyara;
- Bafata ibikoresho bikoreshwa mu isuzuma ry'itsinda bakabibika ahantu hatekanye;
- Basubiza ibintu mu mwanya wabyo mu cyumba, niba ari ngombwa.

Ibindi biganiro by'inyongera (ibi bikorwa iyo bikenewe)

Kugwa no guhita kw'imvura

Muri uyu mukoro-ngiro, itsinda rirahimba amajwi y'uko imvura igwa n'uko ihita; anahagarara kandi anagendagenda hagati mu ruziga rw'itsinda. Arababwira ati: « Twese hamwe tugiye kwigana imvura igwa ». Akuba mu ntoki gutya (abyerekane), noneho yakureba nawe ukabikora. Arakomeza akabikora kugeza ahindukiye akongera kumureba akamwereka ikindi akora. Ahindukira buhoro buhoro aho ahagaze akareba buri wese mu bagize itsinda kugeza batangiye gukubana ibiganza byabo hagati.

Iyo abonye ko ababyeyi bose barimo gukuba mu biganza byabo, arebana n'umubyeyi watangiye ukamwereke ahindure icyo yakoraga nko gukoma mu mashyi byoroheje. Abandi babyeyi bose bakomeje igikorwa cya mbere (gukuba mu biganza) kugeza arebanye na buri wese uko agenda ahindukira mu ruziga. Buri mubyeyi arahindura agatangira gukoma mu mashyi byoroheje.

Komeza gutyo kugeza bakoze ibi bikurikira byose:

- Gukoma ibiganza ku matako;
- Kudiha ibirenge hasi;
- Bagaruke ku gukoma ibiganza ku matako;
- Bagaruke ku gukoma mu mashyi byoroheje;
- Bagaruke ku gukuba ibiganza;
- Bagaruke ku guceceka; azamura ibiganza byombi akabereka imbere yerekana ko adasakurisha ibiganza na gato.

Imiyaga ikomeye ihuha

Umuyobozi w'ikiganiro akura intebe ye ku ruziga kugira ngo umubare w'intebe uriho ube uri munsi ho gato ugereranyije n'abantu bagomba kwicara. Ahagarara hagati mu ruziga noneho akavuga ikintu. Urugero, ashobora kuvuga ati: « Umuyaga mwinshi urahuha umuntu wese hano agira umuvandimwe w'igitsina gore ». Aho umuntu wese ugira umuvandimwe w'igitsina gore ari agomba guhaguruka ku ntebe ye agashakisha indi; usigaye adafite intebe yo kwicaraho niwe uhagararamo hagati. Wa muntu urimo hagati nawe ashaka ikindi cyaranga abantu, akavuga wenda ati: « Umuyaga mwinshi urahuha umuntu wese hano wambaye umwenda urimo ibara ry'umutuku ». Icyo gihe umuntu wese wambaye umwenda urimo ibara ry'umutuku agomba guhaguruka ku ntebe ye agashakisha indi, hagasigara undi muntu hagati mu ruziga. Abisubiremo inshuro zose ashaka. Uyu ni umukino mwiza ufasha mu kwiga urunyuranyurane rw'abantu mu buryo buryoshye kandi butagize icyo bwica.

Umwitozo ngororangingo

Buri mubyeyi aho ari mu ruziga arahaguruka maze akazamura ukuboko kw'ibumoso, nyuma ukw'iburyo; azamura ukuguru kw'ibumoso, nyuma akazamura ukw'iburyo. Abisubiramo inshuro 8, nyuma inshuro 4, agakurikizaho inshuro 2, agasoza abikora inshuro 1. Umuyobozi w'ikiganiro akabara cyane kuri buri nshuro, bakora uwo mwitozo. Hanyuma barongera bagasubiramo uwo mwitozo umuyobozi w'ikiganiro akabara mu rundi rurimi.

Gusangira ibyabanejeje

Buri muntu avuga ikintu gishimishije giherutse kumubaho. Dore ingero: « Nasaruye amashaza ya mbere y'umwaka ». Cyangwa: « Inshuti yanjye yaje kunsura ». Ibi bitera ababyeyi kurushaho kumva bashyikiranye no gusangira ibyo bishimira mu buzima bwabo; buri wese agomba kuvuga icyamunejeje muri make.

Haguruka niba . . .

Atangiza amagambo agira ati: « Haguruka niba ». Akongeraho ikintu icyo ari cyo cyose kugira ngo urangize iyi nteruro, abe ari igifasha ababyeyi kugira amakuru make batanga aberekeyeho cyangwa abe ari n'ikintu cyabasetsa. Urugero ni nka: « Haguruka niba ukunda igitoki ». Cyangwa se: « Haguruka niba ufite umwuzukuru ». Gerageza kuvuga ku bibazo rusange cyangwa byihariye bijyanye no gutwita no kuba umubyeyi. Niba bishoboka, asaba ababyeyi guhera ruhande bagenda babwirana iyo nteruro itangirwa na: « Haguruka niba . . .».

Bara ugeze ku icumi

Uyu ni umukino mwiza ufasha itsinda kugira ubumwe no guseka. Uko bicaye ku ruziga, abasobanurira ko bagiye kubara bazamuka kugera ku icumi. Gusa hari bimwe biri bubagore! Umuntu umwe gusa ni we ushobora kuvuga umubare uwo ari wo wose. Haramutse hagize aho abantu babiri cyangwa batatu bavugira rimwe, ubwo itsinda ryose rihita ritangira bundi bushya kubara uhereye kuri rimwe. Kandi nta muntu wemerewe kuvuga imibare ibiri ikurikiranye. Birashobora ko ahari uwashaka gufasha abandi wenda akoresheje ibimenyetso cyangwa aca amarenga n'umutwe; ababwira ko bidakwiriye. Ikigamijwe aho, ni ukuryoherwa n'iryo hurizo ririmo, ntabwo ari uko bagera ku 10.

East Africa
Preterm Birth
Initiative
UCSF
University of California
San Francisco

UNIVERSITY OF RWANDA

RWANDA
BIOMEDICAL
CENTER
A Healthy People. A Wealthy Nation

REPUBULIKA Y'U RWANDA
UBUMWE - UMURIMO - GUKUNDA IGIHUGU
