# Supplementary material for: Use of a Facilitated Group Process to Design and Implement a Group Antenatal and Postnatal Care Program in Rwanda
Source: J Midwifery Womens Health. 2018 Sep 25;63(5):593–601. doi: 10.1111/jmwh.12871 (PMC6220997; doi:10.1111/jmwh.12871)
Supplement: Supplementary file 3 — Appendix S3. Ibaruke Neza Mubyeyi: Group Antenatal and Postnatal Care in Rwanda [model manual, English translation]. San Francisco, CA: University of California, San Francisco, and East Africa Preterm Birth Initiative, 2017. [file JMWH-63-593-s003.pdf]

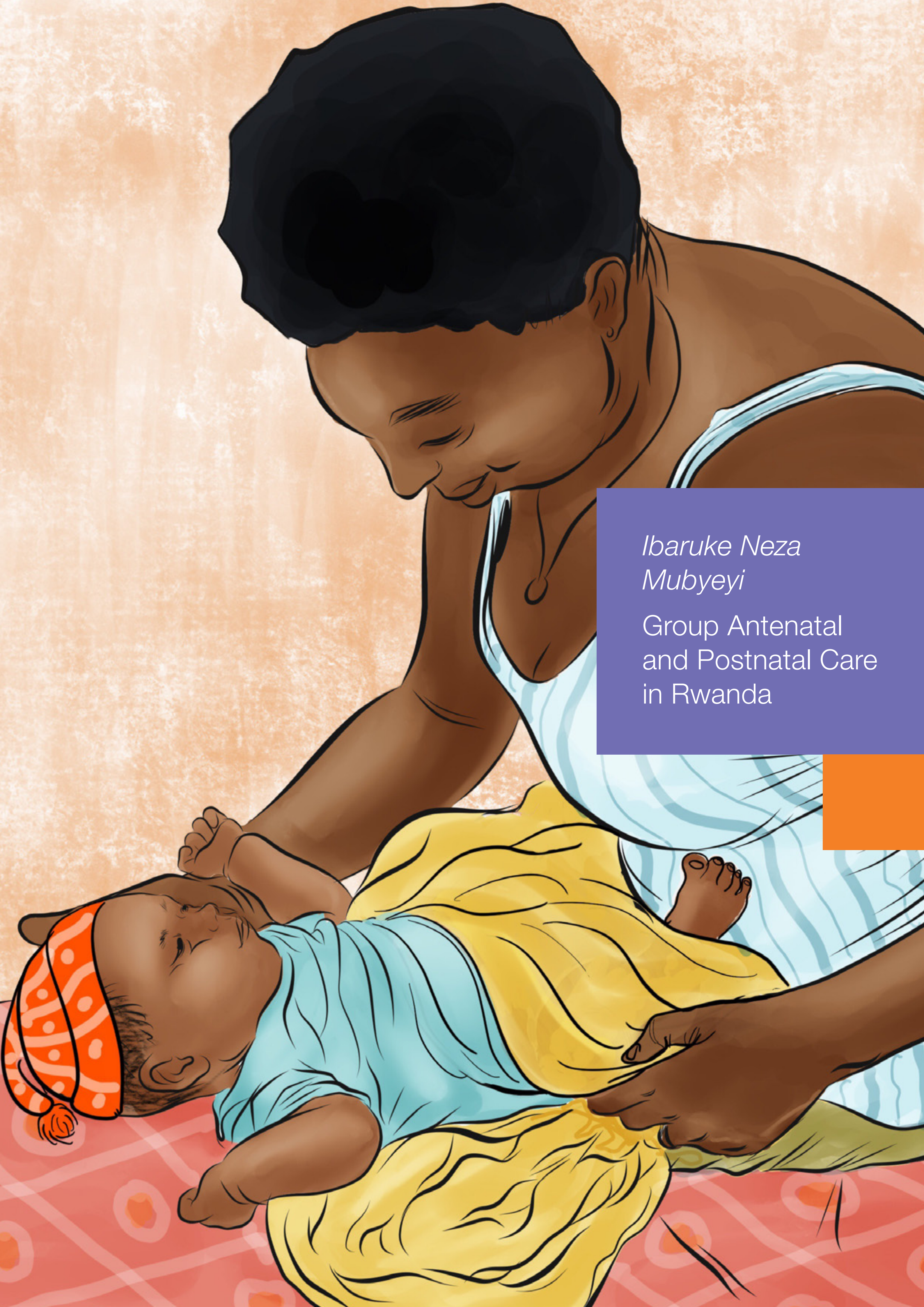

Ibaruke Neza Mubyeyi

Group Antenatal and Postnatal Care in Rwanda

Copyright © 2017 University of California, San Francisco; University of Rwanda and Rwanda Biomedical Center. All rights reserved.

East Africa Preterm Birth Initiative
UCSF Global Health Sciences
550 16th Street, 3rd Floor, Box 1224
San Francisco, CA 94158

Recommended Citation
Ibaruke Neza Mubyeyi: Group Antenatal and Postnatal Care in Rwanda. (2017). The Preterm Birth Initiative East Africa. Kigali: Rwanda Biomedical Center and University of Rwanda. San Francisco: Global Health Sciences, University of California, San Francisco.

Produced in the United States of America. First Edition, July 2017.

This is an open-access document distributed under the terms of the Creative Commons Attribution-Noncommercial License, which permits any noncommercial use, distribution, and reproduction in any medium, provided the original authors and source are credited.

Acknowledgements
This model manual was produced through a collaboration among the Rwanda Ministry of Health, Rwanda Biomedical Center, the University of Rwanda, and the University of California, San Francisco (UCSF) East Africa Preterm Birth Initiative. The East Africa Preterm Birth Initiative is generously funded by the Bill & Melinda Gates Foundation. The content of this manual was developed by the members of the Rwanda GANC/GPNC Technical Working Group:

Evodia Dushimimana
Mathias Gakwerere
Patrick Gatsinzi
Emmanuel Manzi
Catherine Mugeni
Sabine Musange
Assumpta Mwali
Yvonne Delphine Nsaba
Jean Jacques Nshizirungu
Felix Sayinzoga
Providence Umuziga

The Technical Working Group acknowledges the foundational work on group antenatal care accomplished by Sharon Rising and colleagues as described in the book:

Rising S and Quimby C. *The CenteringPregnancy® Model: The Power of Group Health Care*. Springer Publishing Company, 2016.

Contents

Introduction **1**

Key Features of *Ibaruke Neza Mubyeyi* **3**

Preparation for Each Group Visit **6**
1. Chart review 6
2. Gather supplies 6
3. Prepare the group meeting space 7
4. Pre-brief by both co-facilitators 7

Health Assessment Process **9**
ASM role 9
Provider role 9

Group Antenatal Care 2 **12**
Activity 1. Introduction to group antenatal care 12
Activity 2. What is good to eat and drink when you are pregnant? (nutrition) 13
Activity 3: Avoid sickness 13
Activity 4. Pregnancy danger signs 15
Closing activity 15
Alternative activity: Ask the mothers to create a song about what they have learned today 16

Group Antenatal Care 3 **18**
Participant check-in and health assessments 18
Opening activity 18
Activity 1. Mental health 19
Activity 2. Birth plan and signs of labor 20
Activity 3. Birth spacing and family planning 20
Activity 4. Pregnancy danger signs 21
Closing activity 21

Group Antenatal Care 4 **24**
Participant check-in and health assessments 24
Opening activity 24
Activity 1: Labor and birth (and danger signs) 25
Activity 2. Newborn care and danger signs 25
Activity 3: Breastfeeding 27
Activity 4. Postnatal care of the mother and danger signs 27
Closing activity 28

Group Postnatal Care **31**
Opening activity 32
Activity 1. Breastfeeding 32
Activity 2. Healthy family 33
Activity 3. Infant danger signs 33
Activity 4. Immunizations (if immunizations will be given after group visit) 34
Closing activity 34

Introduction

This manual was developed for the use of nurses, midwives, and community health workers (CHWs) who will act as co-facilitators of group antenatal and postnatal care (ANC and PNC) in Rwanda during a cluster randomized controlled trial in 2017–2019. First written in English and then translated to Kinyarwanda, this manual includes basic instructions specific to group ANC and PNC in Rwanda during this period, including orientation to this alternative model of care delivery, step-by-step plans for each visit, and options for activities that help build group cohesion.

The title of this manual, *Ibaruke Neza Mubyeyi*, means "A Mother Achieves a Good Birth" and was chosen by pregnant women in Rwanda who experienced group care. The manual includes several images created by Rwandan artist Dolph Banza, who produced illustrations for the picture cards women use as learning aids during group ANC/PNC in Rwanda.

This manual represents the first implementation of group ANC/PNC in Rwanda and may be changed over time by the partners in future editions.

This document does not include any of the research protocols, data collection tools, or implementation and monitoring plans fundamental to this group ANC/PNC Rwanda project. More information about these materials can be obtained by contacting ptbieastafrica@ucsf.edu.

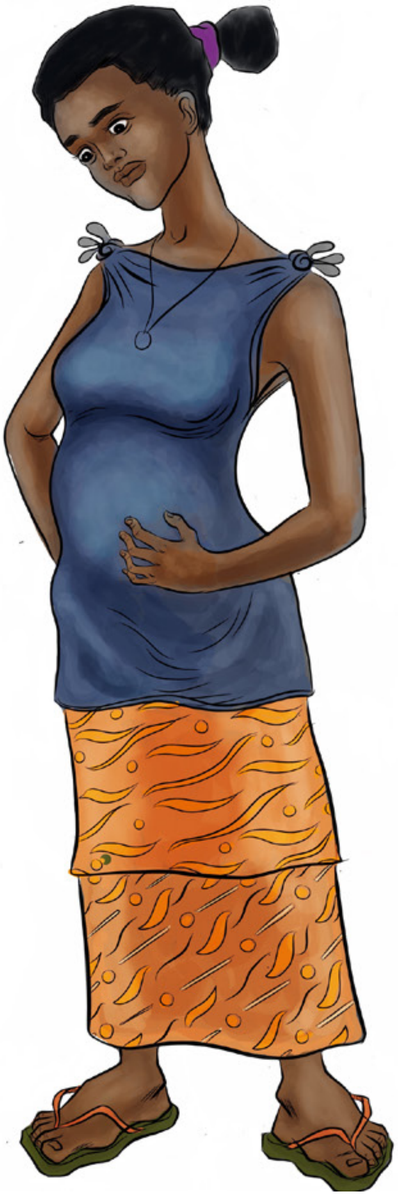

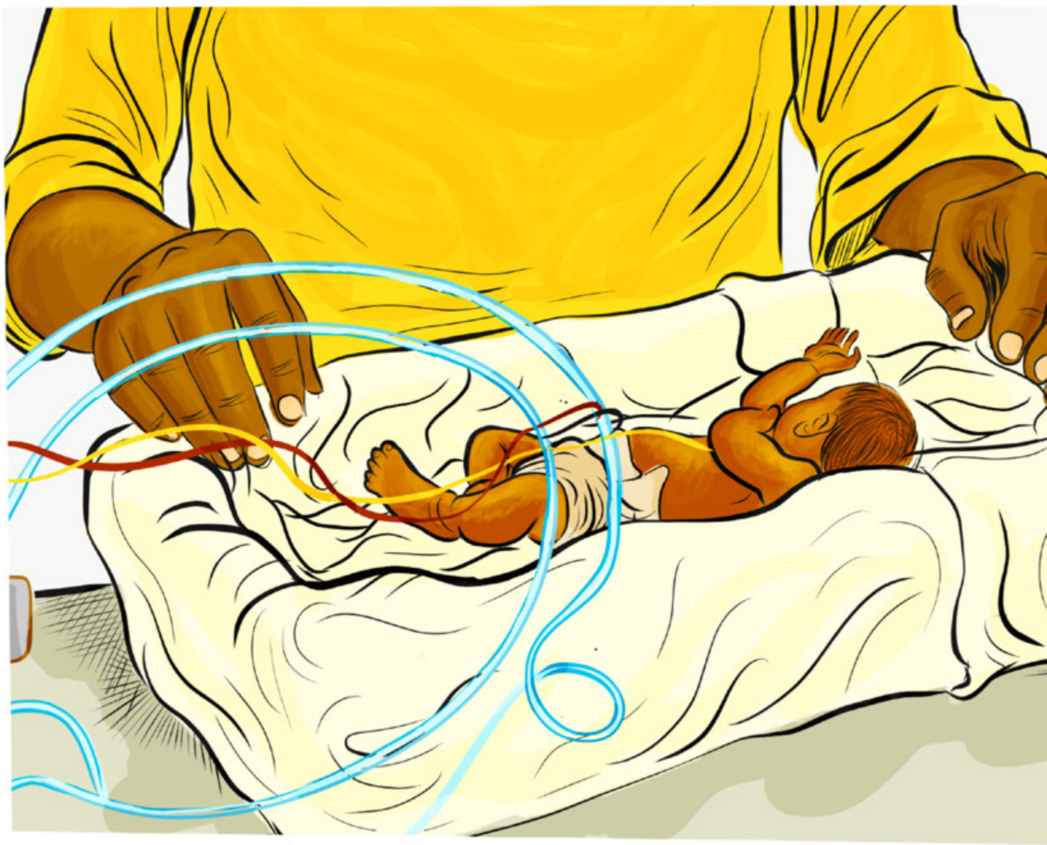

Preterm labor

Illustration developed for *Ibaruke Neza Mubyeyi*

Key Features of *Ibaruke Neza Mubyeyi*

The Rwanda GANC/GPNC Technical Working Group defined the key features of Ibaruke Neza Mubyeyi in 2017. The Group made every effort to preserve the essential elements of this innovative model of care delivery, while maintaining the Rwanda ANC and PNC packages and characteristics of the Rwanda health care system.

1. **Women are organized into consistent groups of 8–12 individuals with the same due month (due dates as close as possible).**

- If possible, special groups are organized for adolescents
- Women are encouraged to attend visits with their consistent group, but may drop in to another group if necessary to make up a missed visit

2. **ANC 1 is a standard, one-on-one visit with a provider—conducted exactly as described in the Rwanda Focused Antenatal Care guide. The following activities are completed:**

- Registration
- Medical file established
- Comprehensive medical, gynecologic, and obstetric history
- Systematic physical examination
- Best calculation of expected delivery date/ gestational age
- Clean catch urine evaluation with dipstick for evidence of albuminuria or infection
- Serum screening
  - » Hemoglobin
  - » Blood type and Rh (this may not be available at all facilities)
  - » RPR and HIV (with partner testing)
- Routine and indicated:
  - » Treatments
  - » Medications
  - » Immunizations
- Referral for specialist consultation as needed
- Health messages and education
- Orientation to *Ibaruke Neza Mubyeyi* for next visit (ANC 2 at 20–24 weeks)
  - » All women eligible for ANC at the health facility are invited to participate in group care
  - » Women are assigned to a group with other pregnant mothers that share a due date in the same month (the closer their due dates, the better)
    - › The meeting times of each group are planned out far in advance on the calendar
    - › Each expecting mother is informed of the dates and times her group will meet over the entire pregnancy and postnatal period
  - » Those attending ANC 1 late in pregnancy are invited to attend the remaining scheduled visits of their assigned group and are also invited to attend the ANC visits they have missed (for example, GANC 2 and/or 3) with different groups
  - » Women are asked to enroll and give consent for data analysis
  - » Those who do not attend any ANC but give birth at the health facility will—before discharge from the facility—be invited to attend a GPNC group at 6 weeks postnatal

3. **ANC 2–4 and PNC (6 weeks) are group visits**

Visit	Timing (Ideal)
GANC 2	20–24 (22) weeks gestation
GANC 3	28–32 (30) weeks gestation
GANC 4	36–40 (38) weeks gestation
GPNC	4–8 (6) weeks post birth

4. **Women sit in a circle in a group space (other staff and patients do not enter during the visit).**

5. **Two co-facilitators lead each group: one ANC provider (midwife/nurse) and one CHW/ Animatrice de Santé Maternelle (ASM). The more stable these group facilitators are across time, the better.**

6. **Confidentiality and mutual respect are prioritized by pregnant women and co-facilitators.**

7. **Clean water is offered to the women to drink. A pitcher and plastic cups are used to serve the water.**

8. **Health assessments are conducted during the first 30 minutes, as women arrive at the scheduled time.**

9. **Women participate in their own health assessments as much as possible (blood pressure and weight).**

10. **Brief consultations with the GANC provider are conducted in a semi-private area of the group space.**

- All women in the group should be assessed within the first 30 minutes.

11. **Women and babies receive the routine treatments described in the ANC/PNC packages, as well as treatments indicated for special conditions such as HIV (PMTCT).**

- Any non-routine treatments are kept confidential, to avoid stigmatization of any woman

12. **Women are referred by the ANC provider to the district hospital for abnormal conditions, as directed by national guidelines.**

13. **Key messages of ANC and PNC are delivered through facilitated discussion in which the women speak more than the facilitators.**

14. **Learning activities are based on principles of adult education, including:**

- Repetition of themes
- Peer-to-peer teaching
- Engagement through several different senses
- Problem-solving through discussion
- Fun and gentle physical activity

15. **Group discussion lasts 1 hour.**

16. **Each group of women decides for themselves if they will invite husbands and next-of-kin to attend group visits.**

17. **Co-facilitators debrief after every group visit in a continuous learning and quality improvement process.**

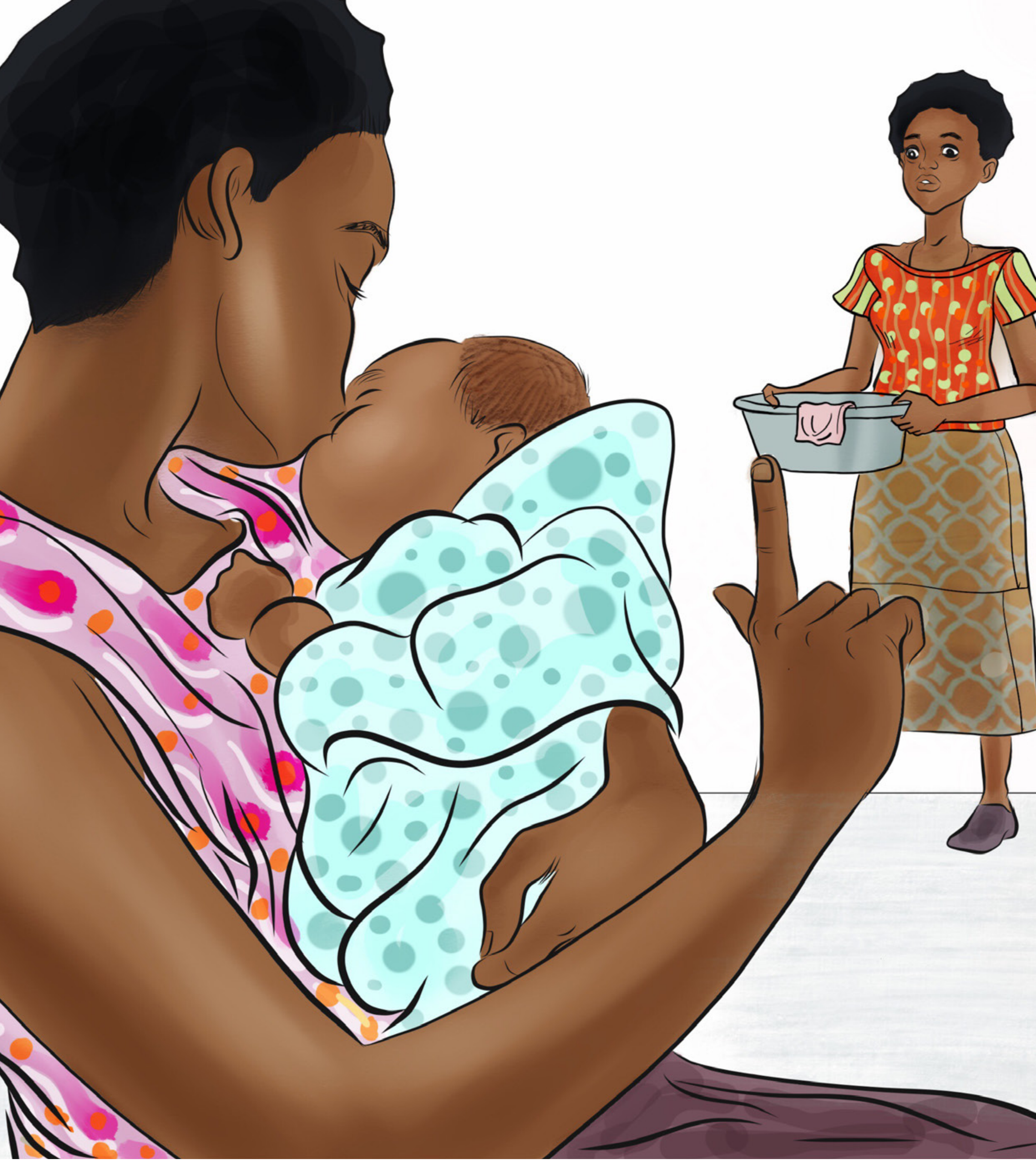

Delay the first bath for at least 24 hours

Illustration developed for *Ibaruke*
Neza Mubyeyi

Preparation for Each Group Visit

On days when you will provide group care, follow these steps to complete a thorough assessment and plan of care for each woman, and prepare well for the group visit. This preparation may require 30 minutes.

1. Chart review

Carefully review each woman's medical file (*Ifishi y'ubuzima*) to plan for the care the woman will need today:

- What is her gestational age today, using a pregnancy wheel and the pregnancy dating information available in her file:
  - » Ultrasound if available?
  - » Last menstrual period if known?
  - » Physical exam at ANC 1?
- Have all her necessary lab tests been done and are the results known?
  - » Hemoglobin
  - » Syphilis & HIV
  - » Albumin in her urine
  - » Blood/Rh group (if available at this facility)
  - » If these have not been documented, test or follow up on results today
- Has she received any previous TT immunization doses and will she require a follow-up TT dose today?
- Does she receive any special treatments you should follow up on today?
- Does she have risk factors that require additional screening or referral?

2. Gather supplies

Prepare bags of iron/folic acid tablets for the women who will come to the session—one bag of iron tablets for each woman you expect to attend. Bring the Mebendazole tablets. Bring TT in a small cooler if possible.

Medication	Dose
Mebendazole 500 mg	1 tablet
Iron & Folic Acid	30–60 tablets
Tetanus Toxoid Immunization	0.5 mL intramuscular injection

Prepare a large pitcher of clean water for the women to drink when they arrive. Place this pitcher on a table with the plastic cups.

Gather the assessment tools you will need.

- The provider will need the following:
  - » Pinnard or other fetoscope
  - » Soft plastic measuring tape (for uterine height)
  - » Thermometer
- The CHW/ASM will need the following:
  - » Adult scale
  - » Electronic blood pressure cuff
  - » MUAC tape

Bring the learning aids you will need for today's group visit. Gather the learning aids that correspond to today's visit number (if you are unsure which learning aids to prepare, study today's visit discussion guide in this manual).

3. Prepare the group meeting space

First choose the best corner of the room for semi-private pregnancy checks. This is generally the corner of the room that is farthest from both the door and the window. Set the exam table and a chair there, surrounded by a privacy screen. Place the provider's assessment tools like the fetoscope and required treatments such as TT immunizations in this space.

Next, arrange the seating (benches or chairs) in a square, triangle, or circle in the room, giving plenty of room for women to enter the circle but still keeping the seats close together to facilitate engagement and discussion. There shouldn't be big spaces between the seated women.

Finally, place a table with one or two chairs just inside the entrance of the group meeting room. Place the following items on the table:

a. Blood pressure cuff
b. MUAC tape measure
c. Pen
d. Individual bags of iron tablets
e. Bottle of Mebendazole 500 mg tablets

Place the scale on the floor nearby. If possible, place the pitcher of water on this table along with enough plastic cups for each of the women.

In some multi-purpose rooms, there may be other furniture, supplies, or equipment that are not related to group care but must always remain in the chosen room. In this case, do all that is possible to make the group care space feel special. Extra seating (for example, benches) may need to be mov;ed to the side of the room during the group care visit.

Use your own good ideas to create a pleasant space for both the pregnant women and the co-facilitators during the group visit. However, don't make the following alterations to the group visit space:

a. Do not complete uterine assessments in a completely private room with a door that closes. It will take too much time and will break up the cohesion of the group. Women can feel their privacy is protected for a routine uterine assessment with the right placement of a mobile privacy screen.
b. Don't arrange the seats in rows. All the group participants should sit in the circle as equals.

4. Pre-brief by both co-facilitators

The two co-facilitators meet for five minutes to plan today's group visit. During the pre-brief, the co-facilitators discuss the outline of activities for the group visit and decide who will lead each activity. The responsibilities should be divided equally. The co-facilitators should take turns leading the learning activities.

Example of how a room can be arranged for a group care visit

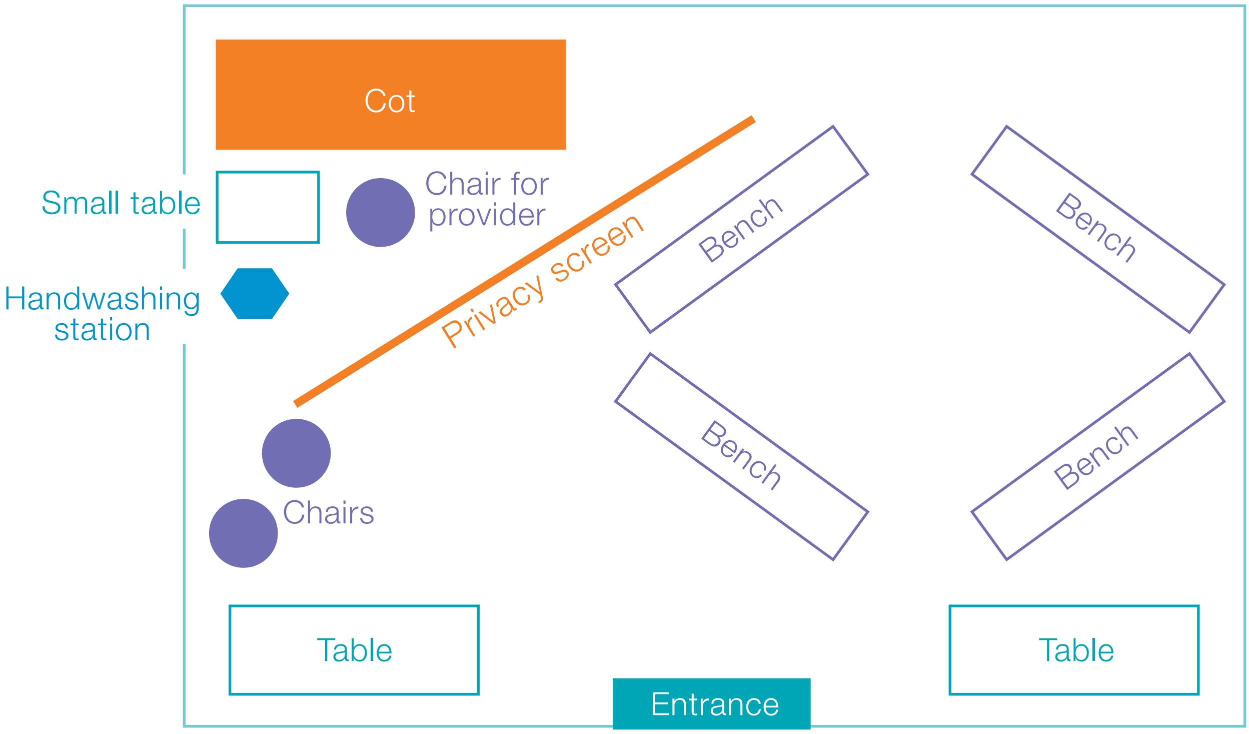

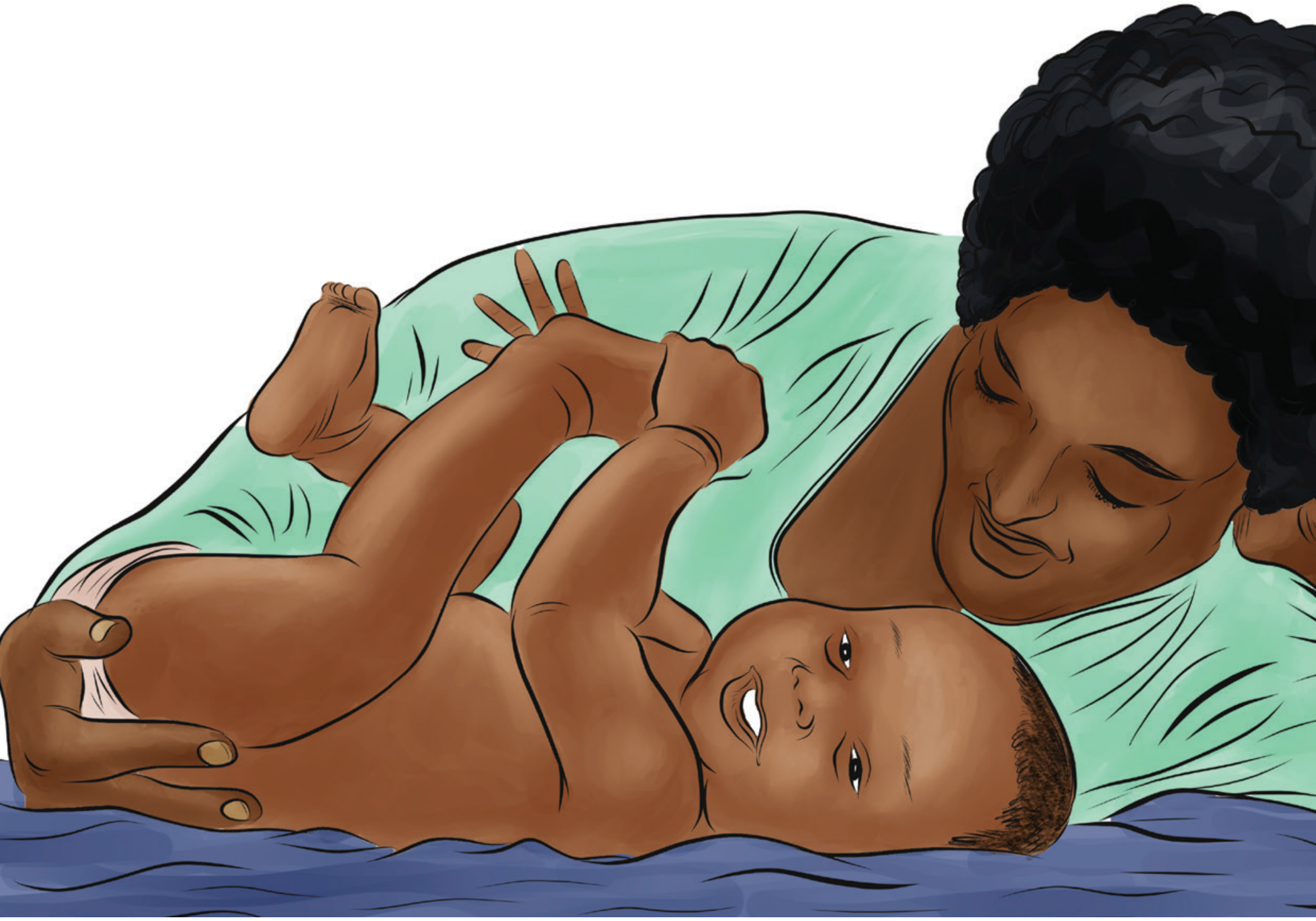

Mother and child play, talk, sing: stimulate
your baby's growing brain

Illustration developed for *Ibaruke*
Neza Mubyeyi

Health Assessment Process

As the women arrive at the appointed group visit time, each will participate actively in her health assessment activities—first, with the Community Health Worker/Animatrice de Santé Maternelle, and second, with the provider. Women will be oriented to this process at the first GANC 2 visit, and thereafter can perform these assessments as they arrive (GANC 3–4 and GPNC) without waiting for all group members to have arrived in order to start.

ASM role

The ASM is responsible for supervising the following:

a. Weight
b. Blood pressure
c. MUAC
d. Mebendazole 500 mg tablet distribution
e. Distribution of iron tablets in bags—one bag to each pregnant woman

The ASM stays near the table at the entrance of the group visit space. The ASM welcomes each woman and supervises activities here, reminding the women how to use the scale, BP, and MUAC equipment and encouraging their participation. The date, weight, BP, and MUAC should be written on the ANC card.

Remind the mother to write the date on ANC card next to the correct visit number. The mother can write the weight, BP, and MUAC in the column called "ICYITON-DERWA/ ICYONGERWAHO," the farthest column to the right.

Some women cannot read, and they should not be singled out or made to feel embarrassed. The ASM and other pregnant mothers should support and help them to be involved in any possible way, to the fullest extent of their abilities.

The ASM should also encourage each woman to drink a tablet of Mebendazole with a large drink of water once each trimester (starting after 16 weeks of pregnancy)—usually at GANC 2 and GANC 3. The ASM explains why this tablet is taken—to keep her healthy by killing any worms that may be in her body.

The ASM invites each woman to take a bag of iron tablets for her use at home. Remind the woman that pregnant mothers will take one iron tablet daily and these tablets should be kept hidden from small children who can die if they find the bag and eat many iron tablets at once.

Each woman spends about 3 minutes completing these activities at the table. Finally, the ASM reminds each woman to take a turn for a pregnancy check with the provider. While she is waiting for her turn, and after her pregnancy check, the pregnant woman sits in the circle and socializes with the other members of the group.

Provider role

Once a pregnant woman has arrived and completed all the assessment activities with the ASM, she waits for a chance to have a brief consultation with the provider.

The provider calls each woman to come behind the privacy screen, one at a time. The provider invites the woman to sit on the exam table, looks at the ANC card and analyzes today's weight, BP, and MUAC and asks about the following danger signs:

- "Do you have any problems, such as difficulty breathing, bleeding, severe abdominal pain or regular cramping, severe headache, vision changes, change in vaginal discharge or a lot of fluid lost, pain when you urinate, genital sores or foul vaginal discharge, or serious illness?"

If the woman responds yes to any of these, explain that she should stay to be examined after the group. If the woman is experiencing an emergency, you may need to arrange for her to be examined immediately. For example:

- If you find an emergency presentation such as very high blood pressure, ask your colleagues or the Titulaire for help to address the emergency. Initiate the correct assessment and/or referral.
- If a non-emergent condition is present, such as an abnormal vaginal discharge, examine the woman privately after the group visit.
- If the woman needs further counseling or care related to PMTCT, invite her to stay after the group for private consultation.

The provider also follows up on any individual issues noted during review of the medical file.

If the woman reports feeling ill, take her temperature with the thermometer now.

In the third trimester, the provider also asks the woman:

- "Is the baby moving normally?"

If the answer is no, investigate whether there is a concern about decreased fetal movement. The woman may need to be referred immediately to the hospital if the baby is not moving normally.

- Complete the assessment documentation in the *Ifishi y'ubuzima* medical file for today's date:
- If the woman she has taken a single Mebendazole tablet today, document this.
- If the woman was given iron and folic acid tablets today, document this.
- If the woman needs a dose of TT today, offer her the immunization now.
- Check today's reported BP and weight, and complete the physical assessment including uterine height and fetal heart beats.

Finally, ask the following:

- "Do you have any other questions or concerns?" If appropriate, ask the woman if you can present her question during the group session so that all the women can benefit from hearing the answer.

Each pregnancy check by the provider should be about 3 minutes. Each visit is brief and to the point, yet the provider is caring and communicates through verbal and non-verbal cues that she is happy the woman has come to the group visit. You may choose to set a timer for each pregnancy check, to keep you on time.

The entire check-in and assessment process should last 30 minutes. If this part of the visit takes longer than 30 minutes, the group visit is unlikely to be successful because the women become bored and there is too little time left for productive discussion.

Once every woman has been checked, all the women and the two co-facilitators sit in the circle together. The co-facilitators should sit across from one another in the circle, for two reasons:

1. The women and co-facilitators will be equal in the circle
2. The co-facilitators can signal easily to each other if necessary

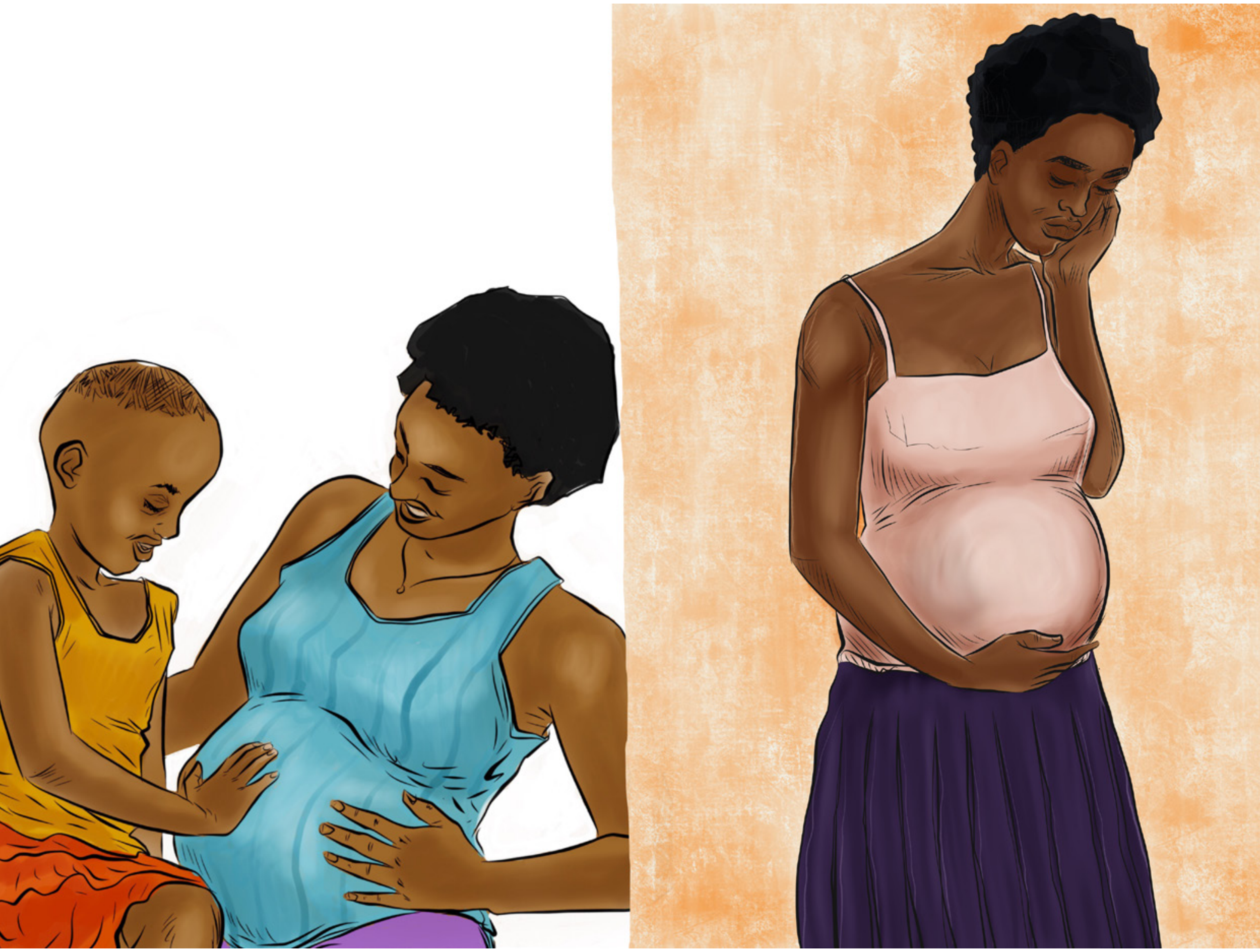

Pay attention to fetal movement

Illustration developed for *Ibaruke Neza Mubyeyi*

Group Antenatal Care 2

Ideal: 22 weeks gestation
Range: 20–24 weeks gestation

Goals for this visit

- Introduction to group care
- Establish pattern for group care health assessments
- Forming group trust and cohesion
- Nutrition and self-care
- Infection prevention
- Birth spacing and family planning
- Pregnancy danger signs
- Closing activity and invitation to return to the next group visit

Supplies needed for this visit

Health assessment tools

- Electronic blood pressure cuff
- Adult scale
- Fetoscope
- Soft plastic measuring tape
- MUAC tape
- Thermometer
- Pen and ANC files
- Timer (optional)
- Any screening tests that women missed at ANC 1
- Pregnancy wheel

Medications

- Large pitcher of clean water and one plastic cup for each woman
- Mebendazole 500 mg tablets
- Iron tablets, divided into individual bags or packages of 30 or 60 tablets each
- Tetanus Toxoid injections as needed

Learning aids

- Models for nutrition activity in a basket and picture card showing traditional medicine
- Picture cards: Pregnancy danger signs
- Mosquito net

Activity 1. Introduction to group antenatal care

45 Minutes

Step 1: Welcome

Say: "Welcome to group antenatal care. The purpose of group care is to learn, share, and help one another during pregnancy. You will also get all the important assessments and treatments to have the healthiest possible pregnancy. When we attend the visits together as a group, we learn more and have more fun!

Step 2: Name game

The group stands in a circle facing each other. Every woman introduces herself. She states her name and a special detail about herself that will help others remember her.

Step 3: Group rules

Say: "It's important that every woman feels comfortable sharing her ideas, feelings, and questions. Since we'll be meeting together four times as a group, or every 8 weeks, let's create some rules for our group, about how we will help each woman feel comfortable sharing her ideas here. What do you think some good rules for our group can be?"

Discuss for 5 minutes. The purpose of this discussion is to clarify the following:

- Everything shared in the group is kept confidential. No one will ever repeat another woman's personal information to a person outside this group. The group members trust each other.
- Every woman will participate
- Every woman is respected and her ideas are important.
- The women may decide at this time to invite husbands and family members or to restrict group membership to the pregnant women only. It is important they decide for themselves—as a group.

Step 4: Storytelling

Say: "During the next 30 minutes, the nurse will call each of you to be checked behind that private screen to check your baby's heart and talk privately for a few minutes there.

"While you wait for your turn, the ASM will help you measure your BP and weight. You can drink water and visit the bathroom if you need it."

"While you wait for these assessments, please find another woman in the group and share a story with each other about something you have experienced in this pregnancy that is different than you expected. Then find out if others in the group have experienced the same."

Step 5: Health Assessments

Complete health assessments for each woman as described in the "Health Assessment Process" on pages 9 and 10.

Step 6: Discussion

When assessments are complete, the co-facilitators join the circle. The co-facilitators should sit across from each other, so they can communicate non-verbally throughout the session (e.g. nodding their heads or gesturing).

The provider leads a discussion about the questions that were asked during pregnancy checks, without revealing which woman asked each question. To close this discussion, ask if there are any other questions from group members. This discussion continues no more than 10 minutes.

Activity 2. What is good to eat and drink when you are pregnant? (nutrition)

Time: 15 minutes

Aims for this activity

- Review the balanced diet (macro and micronutrients) vital to fetal growth and maternal health
- Encourage taking folic acid and iron supplement daily
- Emphasize that vitamin-A containing vegetables are healthy and important to eat
- Discourage over-eating
- Teach the dangers of substance use
- Discourage the consumption of traditional medicine during pregnancy
- Encourage gentle physical activity during pregnancy

Facilitator preparation

- Familiarize yourself with the instructions
- Gather the needed objects into a basket

Objects needed

- Food models, including empty bag of milk
- Picture card with traditional medicine
- Water cup
- Iron/folic acid tablets

Step 1

Pass the basket around the circle. Ask each woman to take out one object.

Step 2

Briefly introduce the topic. Say this: "Every single thing we put in our mouths during pregnancy has the power to do great good or great harm to the baby inside. We will discuss what is good to eat and drink while you are pregnant, and what is bad to eat or drink while you are pregnant."

Ask each woman to name the object she is holding. Ask the group, "Is this good to eat or drink when pregnant?" Also ask, "How can this food be prepared well to eat it when you are pregnant?"

Step 3

If the discussion slows, ask additional questions such as:

- Why do women eat this? (food, iron supplements)
- Why DON'T women eat this if it is healthy and good? (green vegetables, iron tablets, water)
- Why do women take this in? (traditional medicine or self-medication)
- Is it dangerous? (any traditional medicine)
- Is it possible to eat too much during pregnancy? Why?
    - » Emphasize that if a woman gained too much weight, she could be eating too much—and this will create risks for the pregnancy

Step 4 (If there is time)

Ask this: "Have any of you heard the song about nutrition on the radio called 'Indyo yuzuye?' Let's sing it together."

Activity 3: Avoid sickness

Time: 15 minutes

Aims for this activity

- Review the dangers of infections during pregnancy
- Encourage the use of a mosquito net treated with insecticide (ITN), including proper placement and replacement when permethrin treatment expires
- Discuss STIs—their prevention and treatment
- Identify sources of food-born infections

Facilitator preparation

- Familiarize yourself with the following story and discussion questions.

Materials/inputs

- Mosquito net

Step 1: Tell the following short story

Not so long ago, a man named Kamanzi married a clever woman. Soon the wife discovered she was pregnant and told Kamanzi. They were very happy. Kamanzi said, "We will have a very large family!" Kamanzi's wife said to Kamanzi, "I want to have strong healthy children, so when this child is born we must wait 2 or 3 years to have another child." Kamanzi was impressed by his wife's knowledge.

One day, Kamanzi's wife went out to collect clean water. Before she left, she said to Kamanzi, "I have left some food for you to eat. Don't forget to wash your hands with soap before you eat, and keep the food covered, and re-heat the food very hot before you eat it." Kamanzi did not wash his hands nor did he cover the food, and he became very sick with diarrhea, but his wife cared for him and he recovered a few days later.

Kamanzi's wife went to collect water again. Before she left, she said to Kamanzi, "While I am gone, please hang the mosquito net above our bed. I don't want to become infected with malaria while I am pregnant as this could be very dangerous for my pregnancy." As she entered her house again, she saw a strange woman rush out of her bedroom and she saw Kamanzi in the bedroom undressed. Kamanzi's wife was suspicious that he was unfaithful, so she went straight to the health center and asked to be checked and treated for blood and genital infections caused by an unfaithful husband.

When she returned home, Kamanzi's wife fixed the mosquito net properly over her bed herself. She told Kamanzi he couldn't sleep in her bed again until he was checked at the health center for diseases. Then she washed her hands, ate her dinner of beans, isombe, sweet potato, and tilapia, took her folic acid and iron tablet, and fell asleep under the mosquito net.

Step 2

Ask, "What do you think about the words and actions of Kamanzi's wife?" Discuss.

Step 3

Lay the mosquito net in the middle of the circle, spread out. Ask the women to each pick up the edge so they are holding it together.

Say this: "This mosquito net will keep the mosquitos away and prevent malaria, which is a very dangerous illness." Ask, "What are the dangers to pregnant women and their unborn babies when infected with malaria—even if they don't feel sick?" Give the women a chance to provide the answers, and then share correct information: death before or after birth, born too soon, born small and weak.

Ask this: "How do we use these nets in our homes?"

Review: How to properly fix the net on the bed and when it should be changed. The shelf life is indicated on the ITN, usually between 1–3 years.

Step 4

Ask this: "What did Kamanzi's wife do to protect herself from sickness?"

Discuss. Follow-up questions can be:

- What do you do to protect your family from sickness?
  - » Handwashing
  - » Hygiene and sanitation
  - » Clean water
  - » Wash raw vegetables thoroughly with chlorinated water, don't eat undercooked food, re-heat food very hot (gushyushya)
  - » Go to the health center immediately when sick
- What do you think about sex during pregnancy?
  - » Is it OK?
    - › Yes if the woman protects herself from sexual infections like HIV
  - » How do you protect yourself from sexual infections?
    - › Both husband and wife are faithful, abstinence, or CONDOMS
  - » If both Kamanzi and Kamanzi's wife had a negative HIV result at the first ANC visit, could they still become infected with HIV during this pregnancy?
    - › Yes. If the husband or wife is sleeping with other people, the woman and baby could get HIV during the pregnancy.
    - › The only way to protect from this risk is to use condoms every time during sex.
  - » How can you get condoms? How can you plan with your partner to use them?

If possible, distribute male condoms to the women/couples in the group.

Consider providing a demonstration on the correct application of a male condom on a penis model.

Step 5

Ask this: "Kamanzi's wife planned to wait 2 or 3 years to have another child. What do you think of her words?"

Discuss optimal pregnancy spacing of 2–3 years. Ask, "What is the danger of having another baby very soon after this pregnancy?"

Activity 4. Pregnancy danger signs

Time: 15 minutes

Aims for this activity

- Review danger signs and risk factors during pregnancy
  - » Assess knowledge and teach as needed
- Encourage immediate consultation at a health facility in the case of danger signs

Facilitator preparation

- Familiarize yourself with the instructions
- Gather the picture cards for this activity

Materials/inputs: Picture cards—Pregnancy Danger Signs

Step 1

Say this: "Please take one card." Pass the picture cards around the circle, one for each participant.

Step 2

One at a time, each woman shows her card to the group and describes it. Make sure the following points are discussed by the group regarding each dangerous symptom:

- "What is happening to this woman?"
- "Why might this happen?"
- "What should she do next?" or "What should her family do next?"

Closing activity

Time: 5 minutes

Aims for this activity

- The women share what they have learned today and how they will use this new information
- To emphasize the women's close connections and mutual support
- To remind the participants that they will return for another group visit with the same participants in eight weeks
- The group members will decide if husbands and next-of-kin will be invited to future group visits
- To remind the women to participate in community based health insurance

Facilitator preparation

- Familiarize yourself with the instructions
- Prepare baby doll and ball of string

Materials/inputs: Baby doll and ball of string/yarn

Step 1: Deciding whether to invite husbands and next-of-kin to the group visits

Invite the women to stand up in a circle. Thank them for their participation and summarize the key messages of today's visit. Remind them that they have decided to keep all information discussed in the group visit confidential. Ask the women, "What do all of you think about inviting partners and next-of-kin to the future group visits? It is for you to decide if we will invite guests to future group visits. During the next group visit, we will discuss family planning and birth plans. What is your opinion—should we maintain a group of only pregnant women, or should we invite husbands and next of kin to our discussions?" Discuss for one minute and decide together.

Step 2: Creating a web of connection

The facilitator holds the ball of yarn or string. State something brief that you have learned today and how you will use that information to have good health. Now hold tightly to the loose end of the string, and don't let it go. Toss the ball of string to someone across the circle, making eye contact first and making sure she is ready to catch the ball of string. The final web is best if each person tosses the string immediately across from herself. You might start by tossing to your co-facilitator if she/he is across from you, to demonstrate how this is done.

Step 3:

Ask the person who has received the ball of string to say something that she or he has learned today and how she or he will use that information to have a healthy pregnancy and good birth. Then that person throws the ball of string to another in the circle, while holding tightly to the string. Each participant takes a turn sharing and tossing the string until a web is formed.

Step 4

Ask for the ball of string to be tossed back to you once all the women have had a turn. Point out that during the group visits all have become friends and have learned from each other. Now all are connected to each other and have created a web of support.

Step 5

Place the baby doll on the web so it is well-supported and won't fall.

Say this: "What do you think about this web we've created?" If no one can answer, prompt them to discuss how many women together can be stronger and take care of each other and their children.

Step 6: End

Take the baby doll off the web. Instruct the women to set their string down directly in front of their feet on the floor. This will prevent tangling as you wind up the string.

Alternative activity: Ask the mothers to create a song about what they have learned today

Remind the mothers that the group will meet again every 8 weeks for antenatal care and to discuss other important pregnancy topics. Explain that the visit will be like today—with assessment, treatments, and discussions—and that everyone in the group will be expecting to see every other member.

Remind the women of the date and time of the next group meeting. Remind them to keep their Antenatal Card and bring it to the next group meeting. Tell them if they are sick they should come anytime, but if they are well they should come to their next ANC appointment with their group—this group on their appointed day and time.

Finally, remind the women to obtain Community-Based Health Insurance.

Final steps

If anyone needs individual help—for example, a private exam, blood draw, or injection—instruct her what to do next. She will need to stay after the group to receive individualized care.

Once all the women have gone, complete the ANC Register.

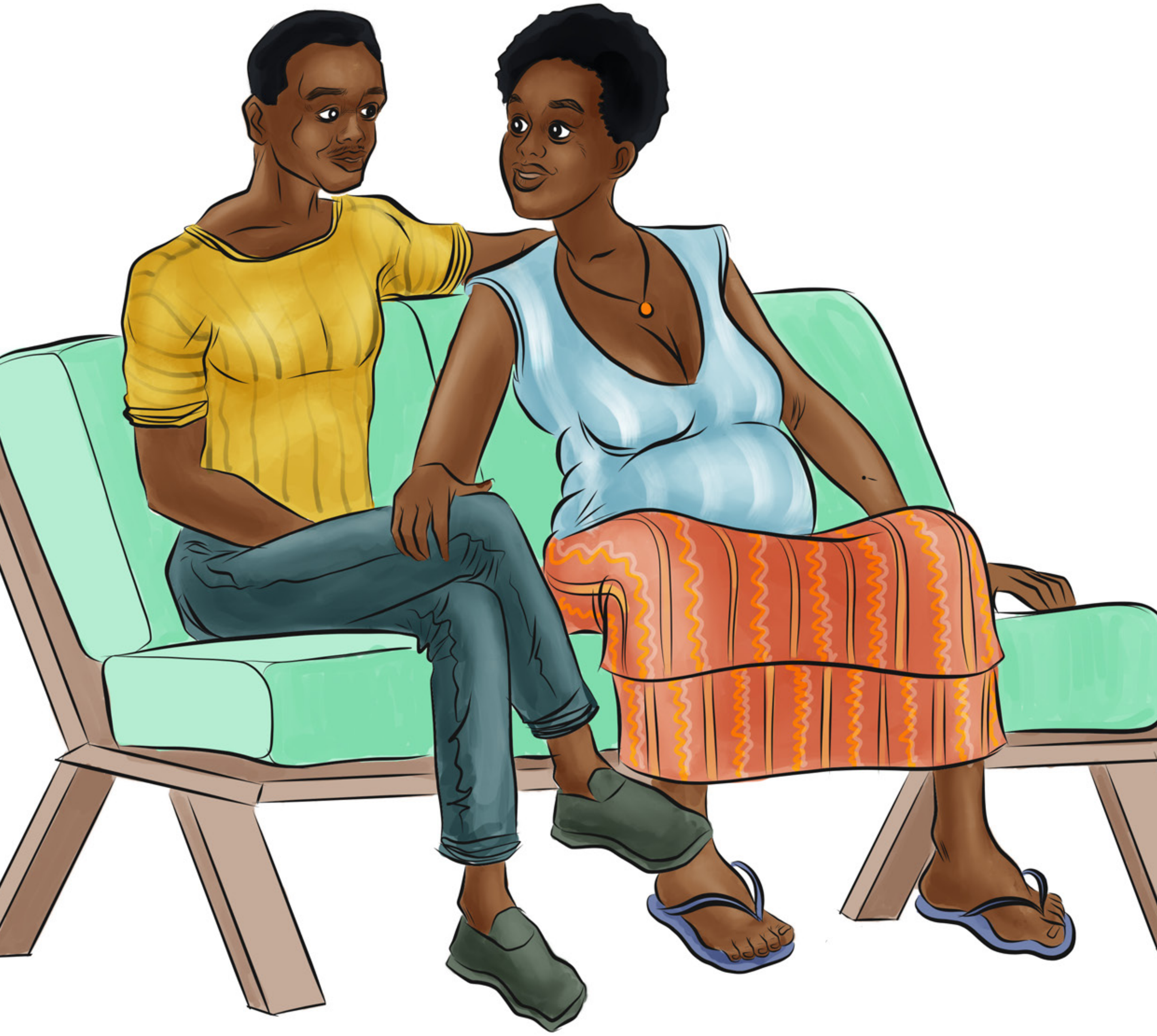

A healthy husband-wife relationship

Illustration developed for *Ibaruke Neza Mubyeyi*

Group Antenatal Care 3

Ideal: 30 weeks gestation
Range: 28–32 weeks gestation

Goals for this session

- Health assessments
- Increase group trust and cohesion
- Discuss maternal mental health
- Birth plan and signs of labor
- Birth spacing and family planning
- Pregnancy danger signs
- Closing activity and invitation to return to the next group visit

Supplies needed for this visit

Health assessment tools

- Electronic blood pressure cuff
- Adult scale
- Pinnard or other fetoscope
- Soft plastic measuring tape
- MUAC tape
- Thermometer
- Pen and medical files
- Timer (optional)
- Pregnancy wheel

Medications

- Large pitcher of clean water and one plastic cup for each woman
- Mebendazole 500 mg tablets
- Iron tablets, divided into individual bags or packages of 30 or 60 tablets each
- TT injections, if needed

Learning aids

- Picture cards: Pregnancy Danger Signs, Maternal Mental Health, Signs of Labor
- Birth plan models in a basket
- Birth control methods models in a basket

Participant check-in and health assessments

Time: 30 minutes

1. As women arrive, greet them warmly and start health assessments.
2. To the women who are waiting for a health assessment or for the group to start, say this: “While you wait for your assessments, find a partner and tell each other about something that has bothered you during pregnancy—something uncomfortable you have felt. Find out if other women have felt this and ask how have they coped with this discomfort?”
3. Complete health assessments (as described on pages 9–10).
4. Invite women to drink water during this activity.

Opening activity

Time: 10 minutes

Step 1: Group rules

Ask the women if they remember the group rules established at the first group. The women should produce the answers and review the group rules.

Discuss. The purpose of this discussion is to clarify the following:

- Everything shared in the group is kept confidential
- Every woman will participate
- Listen to and respect every woman

Step 2: Name game

Even though the group has met once for GANC 2, there may be new members or visitors (husbands or next-of-kin). This activity continues to build group trust and cohesion.

The group stands in a circle facing each other. Every woman introduces herself. She states her name and a special detail about herself that will help others remember her.

Step 3: Mothers' Q & A
Remembering the questions the women asked during semi-private pregnancy checks today, reflect those questions to the group and see if any pregnant mothers know the answers. If they do not, share the correct information with them.

Ask the women if they learned anything from each other today about how to cope with common discomforts of pregnancy. Share correct information if needed. This discussion lasts 10 minutes.

Activity 1. Mental health

Time: 15 minutes

Aims for this activity

- Understand why women have depressive or anxious symptoms
- Identify concerning symptoms if they are present
- Discuss what women and families can do if they feel depressed or anxious
- Discuss how women can care for their mental hygiene

Facilitator preparation

Familiarize yourself with the instructions and gather the picture cards needed for this activity

Materials/inputs: Mental health picture cards

Step 1
Welcome everyone to the group visit. Pass the picture cards around the circle. Say: "Some women feel sad or scared when they are pregnant. These feelings might cause the mother to have poor health. Let's talk about these feelings so we may understand what can be done.

Step 2
Say this: "Each of you has a card. Please explain to the group what the mother feels. Some cards show peaceful feelings, and some show difficult emotions."

Ask each woman about the card she is holding, using open-ended questions such as:

"What experience or feeling does this card illustrate?"

"Has anyone felt this before?"

"What does it feel like in your body when you have this emotion?"

Encourage others in the group to remark on the same emotion/feeling, so the woman holding the card isn't the only one talking about one specific feeling.

Step 3
Ask this: "If a mother feels sad or anxious, or is treated poorly by her husband or family, what can she do?"

Discuss the mental health and social services available to mothers.

Step 4
Ask this: "Have any of you known someone who was pregnant or postpartum who had severe depression, anxiety, or psychosis?" Ask all the women who responded affirmatively follow-up questions such as "How did that person act or what did she/he say?" "How did you know the person wasn't well?" Discuss symptoms of severe depression, anxiety, or psychosis.

Step 5
Ask this: "Why do you think a mother might experience these painful changes in mood and feelings?" Discuss the possibilities: reversible changes in the brain, trauma, loss, etc. Draw women out to discuss whether they think these mood changes are able to be treated and by whom. Discuss stigma and fear about asking for help.

Ask this: "If you were to feel this way, what might you do?" "What could your husband or family members do to help you?" Discuss support, de-stigmatizing mental health problems, and how to access treatment.

Step 6 (optional)
If you think it is appropriate, you might use a "stand-up and sort" activity with items on the Edinburgh Postpartum Depression Scale to help women understand the signs of moderate to severe depression.

Ask everyone to stand up. Say this: "I'm going to make some statements about mood. If you think your model/picture card represents this feeling, raise your hand." When the women raise their hands, ask them to line up on one side with positive emotions and on the other side with concerning emotions. In the end you will have two sides of the circle—the women have moved and sorted themselves into a resilient side and a depressed side.

Read these, one by one:

1. I have been able to laugh and see the funny side of things
2. I have looked forward with enjoyment to things
3. I have blamed myself unnecessarily when things went wrong
4. I have been anxious or worried for no good reason
5. I have felt scared or panicky for no very good reason
6. Things have been getting on top of me
7. I have been so unhappy that I have had difficulty sleeping
8. I have felt sad or miserable
9. I have been so unhappy that I have been crying
10. The thought of harming myself has occurred to me

By this time the women have rearranged themselves into two sides—happy and sad. Assert to the women that if any one of them felt most of these sad emotions

at the same time, and no happy emotions, they should seek help immediately. When these sad emotions are too strong, that's called depression and it is a sickness that can be treated at the district hospital.

Note: This activity may result in strong feelings and disturbing stories among group members. Be prepared to offer the women support and referrals for higher-level mental health care if needed.

Activity 2. Birth plan and signs of labor

Time: 15 minutes

Aims for this activity

- Discuss what each woman plans to do and or/ learn to prepare for the time of her baby's birth.
- Discuss communication with husbands and family members about planning for baby's birth.
- Discuss benefits of health facility delivery

Facilitator preparation

Familiarize yourself with the instructions and gather the needed birth plan models into a small bucket.

Materials/Inputs

- Birth plan models in a bucket:
  - » Baby's cap and shirt
  - » Birth registration certificate
  - » Towel
  - » Kitenge
  - » Mutuelle de Santé card
  - » Soap
  - » Porridge (unopened bag)
  - » Wallet
  - » (optional) Silly object that has nothing to do with birth planning but may make the women laugh
- Picture cards: Signs of Labor

Step 1

Pass the bucket around the circle. Ask each woman to take out an object. The last woman is holding the bucket.

Step 2

Briefly introduce the topic: What does each woman need to prepare for giving birth in a health facility?

Ask each woman to name the object she is holding. Ask the group: "Why will you need this when you give birth?"

Step 3

If the discussion slows, follow up with questions such as:

- Why do women need this?
- If you have already given birth in a health center, what did you wish you had brought with you?
- What would you like to have with you when you give birth?
- What does your husband think about preparing for birth?
- What does your husband's family think about preparing for birth?

Step 4

Pass the empty basket around and ask women to put the object back in. Immediately start passing around Signs of Labor cards.

Step 5

One at a time, each woman acts out the sign of labor on her card. Make sure the following points are discussed for each sign of labor:

- "What is happening to this woman?"
- "What should she do next?" or "What should her family do next?"

Activity 3. Birth spacing and family planning

20 minutes

Aims for this activity

- Discuss rapid repeat pregnancy and risks to the mother and future baby
- Encourage women to make a plan with their husbands to wait two years after this baby's birth to get pregnant again
- Discuss birth control options
- Discuss incorrect opinions about birth control

Facilitator preparation

- Familiarize yourself with the instructions
- Prepare the birth control method examples (in a basket)

Materials/inputs

- Basket with all the possible birth control methods

Step 1: Tell a short story

You remember during the last group visit we heard a story about Kamanzi's wife. Well here is another story about the same clever woman:

Kamanzi's wife gave birth to a beautiful child. Kamanzi's wife had a sister, married to a man named Mugenzi.

Kamanzi's wife eventually had three children. Their ages were 7, 4, and 1 years.

Mugenzi's wife also had three children. Their ages were 3, 2, and 1 years.

Ask this: "What do you think about the ages of Kamanzi's children and the ages of Mugenzi's children?" Discuss the spacing between births.

Step 2
Ask this: "What do you think about spacing the births of your children?"

"What is the healthy time to wait before getting pregnant again?" (2 years) "Why do you think mother and child are healthier when there is a good space between births?"

Step 3
Ask this: "How do wives and husbands control the time between pregnancies?"

Step 4
Pass around the basket of birth control method examples. Each woman picks one from the basket and holds it up for the group to see.

Ask this: "What is this and how is it used?"

Make sure the following are discussed for each method:

1. When the method should be started
2. Benefits and risks of the method
3. Where the method can be obtained
4. How much the methods cost—which are the most cost-effective methods

Step 5
Ask this: "Does anyone have other questions about birth control?" Reflect each question back to the group and let them generate the answer. If they cannot, provide the correct answer.

Activity 4. Pregnancy danger signs

Time: 15 minutes

Aims for this activity

- Review danger signs and risk factors during pregnancy
    - » Assess knowledge and teach as needed
- Encourage immediate consultation at a health facility in the case of danger signs

Facilitator preparation

- Familiarize yourself with the instructions
- Gather the picture cards for this activity

Materials/Inputs: Pregnancy Danger Signs picture cards

Step 1
Pass the Pregnancy Danger Signs picture cards around the circle.

Step 2
Ask the mothers what common discomforts of pregnancy they discussed at the beginning of the visit during health assessments. Use this opportunity to help them understand the differences between harmless discomforts and danger signs.

Ask this: "Will anyone volunteer to teach the group about the pregnancy complication on your card? You can help each other or trade cards with another woman if you would like to."

Encourage the women to teach each other about each symptom—what the symptom is, what it means, and what to do next.

Let the women generate the ideas. If the discussion slows, ask the group another open-ended question.

Step 3
Emphasize that iron and folic acid are taken every day to strengthen the body in case of one of these complications, so the mothers will recover.

Closing activity

Time: 5 minutes

Two options for this activity—choose either:

- Create a song about what we have discussed today
- Web of connection using ball of string and baby doll

Aims for this activity

- The women share what they have learned today and how they will use this new information
- To emphasize the women's close connections and mutual support
- To remind the participants that they will return for another group visit with the same participants in 8 weeks
- To remind the women to participate in community insurance

Facilitator preparation: Prepare the ball of string and the baby doll

Creating a web of connection

Step 1:
The facilitator holds the ball of yarn or string. State something brief that you have learned today and how you will use that information to have good health. Now hold tightly to the loose end of the string, and don't let it go. Toss the ball of string to someone across the circle, making eye contact first and making sure she (or he) is ready to catch the ball of string. The final web

is best if each person tosses the string immediately across from herself. You might start by tossing to your co-facilitator if she/he is across from you, to demonstrate how this is done.

Step 2:

Ask the person who has received the ball of string to say something that she or he has learned today and how she or he will use that information to have a healthy pregnancy and good birth. Then that person throws the ball of string to another in the circle, while holding tightly to the string. Each participant takes a turn sharing and tossing the string until a web is formed.

Step 3

Ask for the ball of string to be tossed back to you once all the women have had a turn. Point out that the women have become teachers that can spread knowledge in their families and communities. Now all are connected to each other and have created a web of knowledge and support.

Step 4

Place the baby doll on the web so it is well-supported and won't fall.

Say this: "What do you think about this web we've created?" If no one can answer, prompt them to discuss how many women together can be stronger and take care of each other and their children.

Step 5: Reminders

Take the baby doll off the web. Instruct the women to set their string down directly in front of their feet on the floor. This will prevent tangling as you wind up the string.

Remind the women that the group will meet again every 8 weeks for antenatal care and to discuss other important pregnancy topics. Explain that the visit will be like today—with assessment, treatments, and discussions—and that everyone in the group will be expecting to see every other member.

Remind the women of the date and time of the next group meeting. Remind them to keep their Antenatal Card and bring it to the next group meeting. Tell them if they are sick they should come anytime, but if they are well they should come to their next ANC appointment with their group—this group on their appointed day and time.

Finally, remind the women to obtain Community-Based Health Insurance.

Final steps

If anyone needs individual help—for example, a private exam, blood draw, or injection—instruct her what to do next. She will need to stay after the group to receive individualized care.

Once all the women have gone, complete the ANC Register.

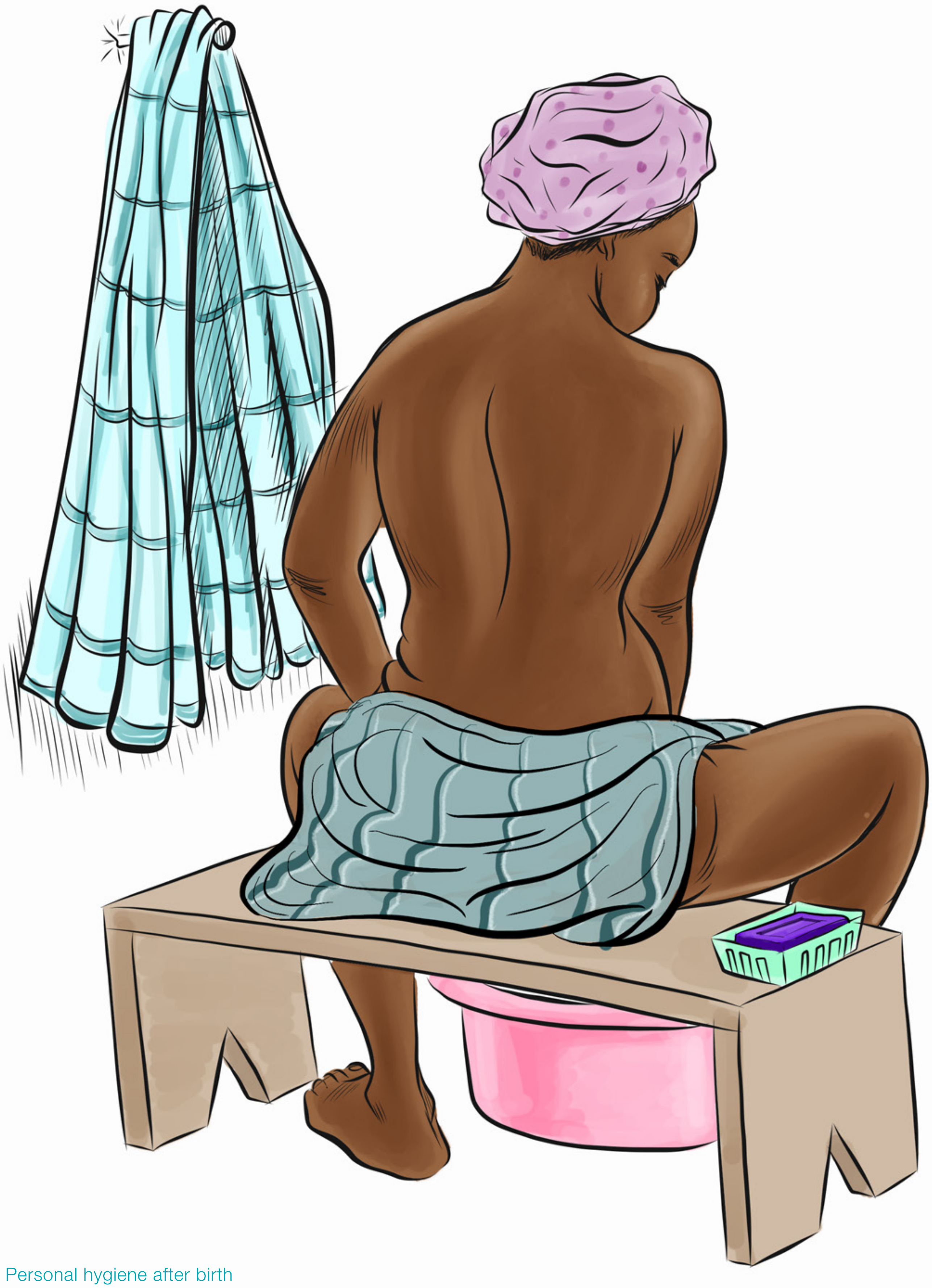

Personal hygiene after birth

Illustration developed for *Ibaruke Neza Mubyeyi* picture cards

Group Antenatal Care 4

Ideal: 38 weeks gestation
Target: 36–40 weeks gestation

Goals for this session

- Health assessments
- Planning for labor and birth, including registering newborn after birth
- Newborn care and parenting, and newborn danger signs
- Breastfeeding
- Birth spacing and family planning
- Pregnancy and postnatal danger signs
- Closing activity and invitation to return to the next group visit (PNC) after the babies are born

Supplies needed for this visit

Health assessment tools

- Electronic blood pressure cuff
- Adult scale
- Pinnard or other fetoscope
- Soft plastic measuring tape
- MUAC tape
- Thermometer
- Pen and medical files
- Timer (optional)
- Pregnancy wheel

Medications

- Large pitcher of clean water and one plastic cup for each woman
- Mebendazole 500 mg tablets
- Iron tablets, divided into individual bags or packages of 30 or 60 tablets each
- Tetanus toxoid injections

Learning aids

- Baby doll
- Picture cards: Pregnancy danger signs
- Picture cards: Postpartum care and danger signs
- Picture cards: Newborn care and danger signs

Participant check-in and health assessments

Time: 30 minutes

1. Ask participants to do the following task while they are waiting for a health assessment. Say this: "While you wait for your assessments, find a partner and tell each other about something that is worrying you. Discuss how to cope with this worry. Perhaps other women in the group worry about the same—how have they coped with this worry?"
2. Complete health assessments (as described on pages 9–10) and **add fetal presentation about Leopold's Maneuvers**.

Opening activity

Time: 10 minutes

Step 1: Group rules

Ask the women if they remember the group rules established at the first group. The women should produce the answers and review the group rules.

Discuss. The purpose of this discussion is to clarify the following:

- Everything shared in the group is kept confidential
- Every woman will participate
- Listen to and respect every woman

Step 2: Name game

Even though the group has met before, there may be new members or visitors (husbands or next-of-kin). This activity continues to build group trust and cohesion.

The group stands in a circle facing each other. Every woman introduces herself. She states her name and a special detail about herself that will help others remember her.

Step 3: Mothers' Q & A

Ask the women what "worries" were discussed in the group during assessments. If their worries are related to incorrect information, discuss those topics until the correct information is presented. Ask this: "What are some strategies to help us feel better when we are worried about something?" Discuss briefly. Possible

strategies are talking to a supportive friend who is generally positive and cheerful; praying, singing, or distracting your mind with another activity you enjoy; breathing deeply; or thinking positive thoughts about yourself, your baby, and your future.

Remembering the questions the women asked during semi-private pregnancy checks today, reflect those questions to the group and see if any women know the answers. If they do not, share the correct information with them.

Activity 1: Labor and birth (and danger signs)

Time: 15 minutes

Aims for this activity

- To encourage each woman to bring a trusted support person with her when she comes to the health facility—someone who can be her advocate
- To review the birth plan
- To review the signs of labor
- To review what to do if a woman accidentally gives birth at home
- To encourage the women to participate in vital records registration

Facilitator preparation

- Familiarize yourself with the instructions

Materials/inputs

- Picture cards: Signs of labor
- Objects in a basket: Birth plan

Step 1

Pass the Signs of Labor picture cards around. Each woman should take one.

As these are passed around, ask this: "How do you hope to be cared for at the health facility when you come to give birth?"

Discuss participant hopes, fears, and questions regarding health facility treatment. Following up on the groups' ideas, ask this: "How can women giving birth make sure they are treated well?" Discuss bringing their husbands or next of kin who can advocate for them, speaking up for what they want and need, and talking to their friends and family about how to prepare for labor.

Step 2

Ask each woman to show her Signs of Labor card and discuss each card as a group. The women should give the answers—either the one holding the card or someone from the group. The facilitator fills in information only when the women themselves can't provide it. Collect the picture cards.

Step 3

Ask the women this: "How have you been preparing for birth?" Discuss important points about preparing for health facility birth, reviewing the detailed birth plan messages discussed in GANC 3. Specifically, review the point about the process and importance of registering the child with the local authority soon after birth. Discuss how other members of the family can help with this registration.

Step 4: Pregnancy danger signs

Pass the Pregnancy Danger Signs cards around the circle. Discuss the Pregnancy Danger Signs cards. Encourage the women to teach each other about each danger sign—what the symptom is, what it means, and what to do next.

Activity 2. Newborn care and danger signs

Time: 15 minutes

Aims for this activity

- Review the importance of the following:
    - » Early and exclusive breastfeeding (This will be reviewed in depth in Activity 3 today)
        - › Within 30 minutes after birth and on demand at least every 2 hours during the first 24 hours of life
    - » Keeping the baby warm
        - › Skin to skin continuously in the first 24 hours after birth and as often as possible thereafter—Kangaroo Mother Care
        - › Keep the baby near mother and do not put far away in a crib
        - › Delay the first bath for at least 24 hours after birth
        - › Close windows
        - › Correct number of clothing layers and swaddling
    - » Don't make the baby too warm
    - » Cord stump clean and dry—do not apply traditional medicine or home remedies
    - » Basic hygiene
    - » Singing, talking, playing, cuddling with baby—to encourage optimal brain development
    - » Sleeping under a mosquito net
    - » Postnatal care services (PNC)
    - » Immunizations at 1 week and 6 weeks of life
- Encourage the woman to hurry to the health facility if the baby shows any of the following:
    - » Trouble breathing or fast breathing
    - » Doesn't eat well
    - » Moves differently—shaking too much or not moving very much (weak)

- Yellow skin
- Umbilical cord is red, hot, or bleeding
- Too warm or too cold

Facilitator preparation

- Familiarize yourself with the instructions and prepare the learning aids

Materials/inputs

- Baby doll
- Picture cards: Newborn care and danger signs

Step 1

Explain that in this activity you will discuss how to care for your new baby.

Pass the baby doll to the participant on one side of you. Say this: "What does this baby need most to grow strong?" Ask the woman holding the doll if she has an idea. If not ask, "Do any of you have an idea? What will you do to keep your baby well and strong?"

Review the following key messages:

- Early and exclusive breastfeeding
    - Within one hour after birth and on demand at least every 2 hours during the first 24 hours of life
    - Keeping the baby warm
        - Skin to skin continuously in the first 24 hours after birth and as often as possible thereafter—Kangaroo Mother Care
        - Keep the baby near mother and do not put far away in a crib
        - Delay the first bath for at least 24 hours after birth
        - Close windows
        - Correct number of clothing layers and swaddling
    - Don't make the baby too warm!
    - Cord stump clean and dry—do not apply traditional remedies
    - Singing, talking, playing, cuddling with baby—brain development
    - Sleeping under a mosquito net

Step 2

Ask, "When does the baby need to be checked by a provider or community health worker?"

Ask women who already have children, "Did you receive postnatal care services?" Hopefully someone in the group will answer yes. Ask her to tell her experience with postnatal care services. Respond to her story with correct details about the purpose and benefit of PNC.

If the conversation slows, ask "What kinds of questions did you have about baby care in the days and weeks after your baby was born?" Say, "Postnatal care services can help you know the answers to these questions." Discuss what postnatal services are available and why they are useful to the women and babies.

The ASM can describe in more detail how to coordinate postnatal care at home on day three and day seven of life.

Step 3

Ask the woman who is holding the baby doll, "Do you think the baby hears or understands what the mother is doing? How do the mother's actions affect the baby?"

Generate a discussion about the importance of talking to and playing with the baby, even when they baby is very young. Singing to the baby is great! Making eye contact with the baby frequently is important. All this stimuli—talking, singing, playing—is absorbed by the baby and the baby's brain grows stronger and smarter.

Put the doll away.

Step 4

Pass around the Newborn Danger Signs picture cards. Say this, "Please take one card." Pass the picture cards around the circle, one for each participant.

One at a time, each woman talks about the newborn care or complication on her card. The other women in the circle can be asked to give input as well. Make sure the following points are discussed for each dangerous symptom:

- "What is the condition of this baby?"
- "What is dangerous in this baby's condition and what might happen to this baby if the dangerous condition is not corrected?"
- "What should the parents do for this baby?" (Go to the health facility)

After the activity, collect the picture cards.

Step 5

Ask this: "What are some common problems newborns have that aren't dangerous? What is the safest way to help the newborn feel better?"

Discuss common problems like constipation, sore throat, or rash. Discourage the use of traditional medicine, cutting, or tonsillectomy. Encourage bringing the newborn to the health facility if the mother has any concerns.

Activity 3: Breastfeeding

Time: 15 minutes

Aims for this activity

- Encourage skin-to-skin placement of the baby on the mother's chest/abdomen to warm the baby and initiate breastfeeding within the first hour after birth
- Encourage exclusive breastfeeding for the first six months of the baby's life
- Discuss optimal breastfeeding positions and latch
- Discuss incorrect beliefs about infant feeding
- Discourage early introduction of non-breastmilk foods or liquids
- Address concerns such as breast pain
- Encourage frequent, nutritious meals for the mother
- Encourage continued iron and folic acid supplementation until the baby is 3-months-old

Facilitator preparation

- Familiarize yourself with the instructions

Materials/inputs: Baby doll

Step 1

Pass the baby doll around the circle again. Invite each woman, when she is holding the baby doll, to say something she knows or has heard about breastfeeding, or to teach the group how to breastfeed by using the doll to demonstrate.

If the woman says very little, ask follow-up question to the group—such as: "What have you all heard about that?" or "Do any of you know more about that?"

If a woman states something incorrect about breastfeeding, ask the group for their input, opinions, or ideas about that mis-statement. Allow the group to correct the mistake. If no one in the group corrects the mistake and it is a vital point to make, offer the correct information.

Step 2

If none of the common myths about breastfeeding have been discussed, bring them up now. You can ask this: "Have any of you ever heard that..." "What do you think about that statement?" "If a grandmother or a very young woman said this to you, how would you correct her opinion?"

These myths include the following:

- Breastfeeding always hurts
- There is no breastmilk for the first two days after birth so the baby must have other fluids
- My breasts might be dry and won't make enough milk
- Mothers who are sick should not breastfeed their babies
- If a baby cries, he doesn't like the breastmilk. Or if the baby vomits, it is caused by breastmilk.
- Never put the baby on the breast immediately after birth because you must wait until the breast is full of milk.

Step 3

Discuss early initiation of breastfeeding and common mistaken opinions about not having enough milk for the baby immediately after birth.

Ask this: "When should the baby start breastfeeding after birth?" Encourage early initiation of breastfeeding within the first hour of life. Emphasize that the baby should suckle often during the first day of life to stimulate the mother's body to make plenty of breastmilk.

"How do you know your baby drinks enough breastmilk?" Discuss. The baby urinates and defecates frequently showing that it is receiving plenty of milk.

Step 4

Ask if any of the mothers know how to breastfeed. Ask one of them to demonstrate with the doll.

Ask follow-up questions after her explanation, such as these:

"What are the breastfeeding positions?"

"What shows you that the baby is well attached to the breast?"

Activity 4. Postnatal care of the mother and danger signs

Time: 15 minutes

Aims for this activity

- Review the importance of the following:
  - » Keep your body clean
  - » Breastfeed your baby every day as many times as possible (at least 10 times per day)
  - » Eat at least four times a day and drink a lot of liquids
  - » Take iron/folic acid tablet daily
  - » Sleep with your baby under a mosquito net
  - » Access PNC and return for the group visit 6 weeks after your baby is born
  - » Plan how long you will wait to have another baby—at least two years
  - » Choose a family planning method before you give birth—such as a PPFP option
  - » Seek support and help from family and friends
- Encourage the woman to hurry to the health facility if she shows any of the following:
  - » Bleeding too much
  - » Persistent headache

- » Severe pain anywhere
- » Fits
- » Fever or feeling very ill
- » Red or hot breasts
- » Difficult or painful urination
- » Severe depression or psychosis

Facilitator preparation

- Familiarize yourself with the instructions and gather the learning aids for this activity

Materials/inputs

- Picture cards: Healthy Postnatal Mothers, Postnatal Danger Signs
- Family planning models in a basket

Step 1

Ask the women to stand up in the circle. Pass out all the picture cards at once.

Ask each woman to look at her card and decide if the card shows a healthy postnatal mother or a postnatal mother with a dangerous problem.

Ask the women to move to your left if they have cards showing healthy mothers and to your right if they have danger signs cards. The women should help each other if they are unsure. They will change positions in the circle and sort themselves into a "healthy" side and a "danger signs" side. Ask them to sit again.

Step 2

Start with the "healthy mothers" side of the circle. Ask each woman in turn to describe card she is holding. Emphasize the healthy behaviors.

When personal hygiene is mentioned, ask a few follow-up questions such as:

- "What do you think about washing yourself frequently after giving birth?" Discuss women's traditional and religious beliefs about washing. Encourage frequent bathing of perineum with clean water.
- If sex is not mentioned, ask about it. Ask this: "What do you think about the traditional sex on the day of the baby's naming? Is this too soon to have sex again after birth?" Discuss.

When family planning is mentioned, ask this:

"What is the healthy number of years to wait between births?" Answer: 2–3 years

"Which birth control methods can women get immediately after giving birth in the health facility?" Discuss immediate postpartum family planning methods.

Step 3

Next, the mothers with the danger signs cards describe them. Ask the group this: "What would you do if this happened to you?" The answer is GO TO THE HEALTH FACILITY.

Ask this: "What if this happened to you and you were too weak to act? Who would help you? Does that person know when to take you to the health facility?" Emphasize that the mothers should teach their husbands and next-of-kin about these danger signs.

Closing activity

Time: 5 minutes

Two options for this activity—choose either:

- Create a song about what we have discussed today
- Web of connection using ball of string and baby doll

Aims for this activity

- To encourage the women to reflect on what they have learned and how they will use their new knowledge
- To emphasize the women's close connections and mutual support
- To remind the participants that they will return for another group visit with the same participants in 8 weeks—after their babies have been born
- To remind the women to participate in community insurance

Facilitator preparation

- Familiarize yourself with the instructions
- Prepare baby doll and ball of string

Step 1: Creating a web

The facilitator holds the ball of yarn or string. State something brief that you have learned today and how you will use that information to have good health. Now hold tightly to the loose end of the string, and don't let it go. Toss the ball of string to someone across the circle, making eye contact first and making sure she is ready to catch the ball of string. The final web is best if each person tosses the string immediately across from herself. You might start by tossing to your co-facilitator if she/he is across from you, to demonstrate how this is done.

Step 2

Ask the person who has received the ball of string to say something that she or he has learned today and how she or he will use that information to have a healthy pregnancy and good birth. Then that person throws the ball of string to another in the circle, while holding tightly to the string. Each participant takes a turn sharing and tossing the string until a web is formed.

Step 3

Ask for the ball of string to be tossed back to you once all the women have had a turn. Point out that during the group visits all have become friends and have learned from each other. Now all are connected to each other and have created a web of support.

Step 4

Place the baby doll on the web so it is well-supported and won't fall. Review key messages from today's group visit.

Step 5: Reminders

Take the baby doll off the web. Instruct the women to set their string down directly in front of their feet on the floor. This will prevent tangling. As you wind up the string, remind them that the group will meet again in 8 weeks for postnatal care, immunizations, and to discuss other important parenting topics. Explain that the visit will be like today—with assessment, treatments, and discussions—and that everyone in the group will be expecting to see every other member.

Remind the women of the date and time of the next group meeting. Remind them to keep their Antenatal Card and bring it to the next group meeting. Tell them if they are sick they should come anytime, but if they are well they should come to the 6-week PNC appointment with their group—this group on their appointed day and time.

Finally, remind the women to obtain Community-Based Health Insurance. Also, remind them to report with the newborn's birth certificate at the sector level within 15 days of the birth—point out that the husband, next-of-kin, or other family member can do this for the mother while she recovers.

Final steps

If anyone needs individual help—for example, a private exam, blood draw, or injection—instruct her what to do next. She will need to stay after the group to receive individualized care.

Once all the women have gone, complete the ANC Register.

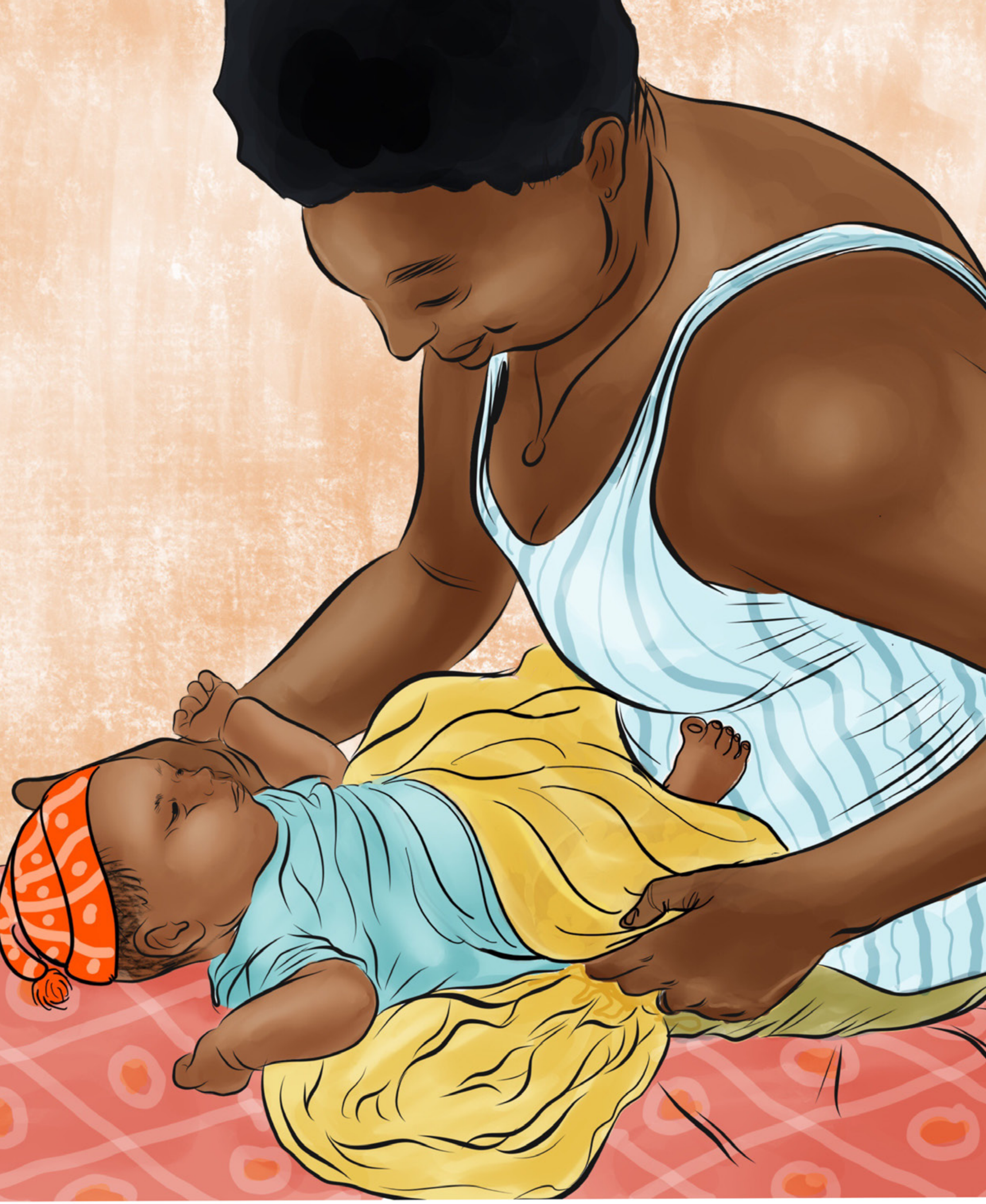

Keep the baby warm

Illustration developed for *Ibaruke Neza Mubyeyi* picture cards

Group Postnatal Care

Ideal: 6 weeks after birth
Range: 4–6 weeks after birth

Goals for this session

- Health assessments—both mother and baby
- Women socialize and introduce their babies to the group
- ITN use
- Birth spacing and family planning
- Newborn care and parenting
- Breastfeeding
- Newborn danger signs
- Closing activity and goodbye

Time needed for this session: 2 hours

Supplies needed for this visit

Health assessment tools

- Newborn scale
- Newborn stethoscope
- MUAC tape
- Thermometer
- Pen and medical files
- Timer (optional)

Immunizations for newborns

- OPV
- DTP-HepB-Hib
- Pneumococcal Conjugate
- Rotavirus

Immunization for mothers when indicated

- Tetanus toxoid

Medication for the mothers

- Vitamin A 200,000IU capsules for mothers who were not already given a dose immediately after birth—only give vitamin A dose if there is no risk the mother is pregnant again

Learning aids

- Birth control methods in a basket
- Baby doll
- Picture cards: Newborn care and danger signs

Chart review before visit—use the medical files in the binder

- Has the mother received Tetanus Toxoid injection?
  - » Does the mother need a dose of TT today?
  - » If so, prepare it and be ready to administer it during her exam or immediately after the group
  - » If not, prepare the date when she should return for her next TT injection—you will give her this date during check-in
- Did the mother indicate during ANC that she desired postnatal family planning?
  - » Which method? Will she receive it today?
- Does the mother have risk factors that require postnatal referral?

Participant check-in:

Time: 30 minutes

1. Participants will relax with their infants in the circle today while they wait—there is no other activity during health assessments. They will likely socialize spontaneously.
2. Complete health assessments (as described on pages 9–10) and today you must add:

- Ask to see mother's ANC card. Use this to review:
  - » Baby's health history
- Complications?
- Did the baby receive any PNC visits after discharge from the facility?
- Was baby born term or preterm?
- Does the baby need referral for any reason?
- Did baby already receive immunizations?
  - » Immediately after birth: BCG, OPV
  - » Has baby already received DPT/HepB/HIB, Rotavirus, 2nd dose OPV, Pneumococcal?
  - » If not, these must be ready to give to the baby today if 6 weeks old
  - » Mother's health history
- Complications?
- Does the mother need referral for any reason?

- Does the mother already have a family planning method? If not, does she want to receive a family planning method today?
- Did mother already receive Vitamin A dose?
  - » If not, give today
  - » Do not give after 8 weeks postnatal if she is not using a birth control method
- Baby's examination
  - » Assess baby's general condition
  - » Ask about the baby's feeding and encourage exclusive breastfeeding
  - » Weigh and assess baby's weight gain
  - » Assess social smiling and visual fixing and following
- Mother's assessment
  - » Ask if she has any problems
  - » Ask if she has been depressed
  - » Ask if she has planned to use a contraceptive method
  - » If appropriate, invite her to stay after the group to receive her contraceptive method of choice or give her an appointment to return to obtain contraception

Opening activity

10 minutes

Step 1: Group rules

Ask the women if they remember the group rules established at the first group. The women should produce the answers and review the group rules.

Discuss for 5 minutes. The purpose of this discussion is to clarify the following:

- Everything shared in the group is kept confidential
- Every woman will participate
- Listen to and respect every woman

Step 2: Babies

Going around the circle, ask the women to introduce their babies to the group, telling the baby's name and something unique about the baby or her birth story. It could be something the baby does, a story about the baby's sibling or other family member, or something she appreciates about her baby.

Step 3: Mothers Q & A

Remembering the questions the women asked during health assessments today, reflect those questions to the group and see if any women know the answers. If they do not know the answers, share the correct information with them.

Activity 1. Breastfeeding

Time: 15 minutes

Aims for this activity

- Encourage exclusive breastfeeding for the first six months of the baby's life
- Discuss incorrect beliefs about infant feeding
- Discourage early introduction of non-breastmilk foods or liquids
- Address concerns such as breast pain

Facilitator preparation:

- Familiarize yourself with the instructions.

Materials/inputs: Baby Doll

Step 1

Ask this: "Does anyone have a question about feeding the baby?"

When a woman has shared her question, ask the group to answer if they can. For example, ask:

"What have you heard about that?"

"Do any of you know more about that?"

If a woman states something incorrect about breast-feeding, ask the group for their input, opinions, or ideas about that misstatement. Allow the group to correct the mistake. If no one in the group corrects the mistake and it is a vital point to make, offer the correct information.

Step 2

If none of the common myths about breastfeeding have been discussed, bring them up now. You can ask this: "Have any of you ever heard that..." "What do you think about that statement?" "If a grandmother or a very young woman said this to you, how would you correct her opinion?"

These myths include the following:

- Breastfeeding always hurts
- There is no breastmilk for the first two days after birth so the baby must have other fluids
- My breasts might be dry and won't make enough milk
- Mothers who are sick should not breastfeed their babies
- If a baby cries, he doesn't like the breastmilk. Or if the baby vomits, it is caused by breastmilk.

Step 3

Ask this: "Why is only breastmilk recommended for babies?"

Ask this: "When do babies need more than breast-milk?" Discuss healthy infant feeding practices.

Activity 2. Healthy family

Time: 20 minutes

Aims for this activity

- Congratulate the mothers for demonstrating healthy parenting behaviors
- Discuss parenting behaviors that stimulate optimal infant brain development, including singing, talking, playing, and bonding—by both father and mother, as well as other caregivers
- Encourage sleeping under mosquito net to prevent malaria infection
- Plan for optimal child spacing (two years before becoming pregnant again) and birth control method
- Encourage health insurance coverage for the child and all members of family

Facilitator preparation

- Familiarize yourself with the instructions and gather the supplies

Materials/inputs

- Baby doll
- Birth control methods in a basket
- Picture cards: Newborn/Infant Danger Signs
- Mosquito net

Step 1

Start a discussion about ITN using open-ended questions, for example "Do you know mothers who sleep with their babies under ITN?" or "Do you know mothers whose babies have become very sick with malaria?"

Ask open-ended questions to stimulate more discussion about ITN use. For example, "Is it difficult to get an ITN?" or "Is it uncomfortable to sleep under the ITN? How do you make it more comfortable?"

Optional: Ask one of the mothers to demonstrate how to properly install and use the mosquito net.

Step 2

"Do any of you know a woman who had babies very close together?" Discuss.

Ask this: "What is the healthiest interval between pregnancies?" Discuss waiting at least two years to get pregnant again.

Ask this: "How can women control when they get pregnant again?" or "What are the methods for preventing pregnancy for two years?" Discuss birth control options. If women are not able to generate all the birth control methods, pass the basket of birth control models around the circle and ask women to describe the methods. Alternatively, if the group has a question about a particular birth control method, take it from the basket and use the model to explain the method in detail.

Step 3

Ask follow-up questions. For example, "When you were a child, did someone sing, talk, and play with you? What effect did that have on you?" Have you seen parents who sing, talk, and play with their children? How do those children behave as they grow?" Discuss.

Ask the mothers if they know a song to sing to babies. If one of the women volunteers offers, a song, ask her to teach it to the group. Sing it together. Practice it a couple of times if necessary. Hold the strong baby doll as if it is a real baby. Demonstrate how to sing to the baby, talk to the baby, and engage the baby face to face.

If the mothers do not volunteer to teach the group a song, offer to teach them one. [SING]

Ask this: "Did you know that singing to a baby helps the baby grow strong?" This stimulation helps their brains to grow strong, even when they don't seem to understand. Their brains need interesting activities, like hearing their mothers sing to, talk to, and play with them. If they don't have this stimulation, their brains will not develop as fully.

"When we sing, talk, and play with our young children, it actually helps them to be smarter when they are grown. This is the first step to fulfilling our dreams for them."

Activity 3. Infant danger signs

Time: 15 minutes

Aims for this activity

- Review the importance of sleeping under a mosquito net
- Encourage the woman to hurry to the health facility if the baby shows:
  - » Trouble breathing or fast breathing
  - » Doesn't eat well
  - » Moves differently—shaking too much or not moving very much (weak)
  - » Yellow skin
  - » Umbilical cord is red, hot, or bleeding
  - » Too warm or too cold

Facilitator preparation: Familiarize yourself with the activity instructions

Materials/inputs

- Picture cards: Newborn/Infant Care and Danger Signs

Step 1

Pass around the "Newborn/infant care and danger signs" picture cards. Say, "Please take one card." Pass the picture cards around the circle, one for each participant.

One at a time, each woman talks about the infant complication on her card. The other women in the circle can be asked to give input as well. Make sure the following points are discussed for each dangerous symptom:

- "What is the condition of this baby?"
- "What is dangerous in this baby's condition and what might happen to this baby if the dangerous condition is not corrected?"
- "What should the parents do for this baby?" (Go to the health facility)

After the activity, collect the picture cards.

Step 2
Ask this: "How can mothers protect their babies from sickness?" Make sure in the discussion the following points are mentioned, if they have not already been discussed today:

- Sleep under mosquito net
- Wash hands frequently with soap
- Give only breastmilk for first 6 months of life
- Immunizations

Step 3
Ask this: "What are some common infant problems that many babies have but they are not truly sick?"

Ask this: "What is the best thing to do when this happens?"

Discuss. Be sure the most common concerns are discussed, such as colic (icyomunda), sore throat, constipation, and skin rashes.

Discuss the best responses to common problems. Discourage the use of traditional medicine, tonsillectomy by traditional healers, or early introduction of foods other than breastmilk.

Step 4
Ask this: "Do you have any other questions today?"

Encourage the women to ask questions about anything related to childbearing or parenting, and reflect the questions back to the group. If no one in the group knows the answer, provide the answer.

Activity 4. Immunizations (if immunizations will be given after group visit)

Time: 5 minutes

Aims for this activity

- Explain the immunizations that will be given today
- Encourage the mothers to make sure the baby gets a vaccine at 9 months of age (measles)

Facilitator preparation

- Familiarize yourself with the instructions and arrange for the immunizations to be ready

Step 1
Say this: "Today we have immunizations to give the babies. These immunizations protect your baby from many dangerous illnesses."

Say this: "These babies all need to return for another immunization when they are 10 weeks old, 14 weeks old, nine months old, and 15 months old."

Make sure each woman has a child immunization card. Point to the card when you explain the other days she will come for immunization.

Ask if there are any questions about vaccines.

Closing activity

Time: 5–10 minutes

Two options for this activity—choose either:

- Create a song about what we have discussed today
- Sharing (instructions below)

Aims for this activity

- The women share what they have learned today and how they will use this new information

Materials/inputs: None

Step 1
Remind the women to maintain their Community-Based Health Insurance, in case their child has a health problem and needs facility care.

Step 2
Everyone in the circle shares one parting message with the group. Each person states something brief that she/he has learned today and how she/he will use that information to enjoy good health for both herself/himself and the new baby.

Step 3
Thank the women for joining the group and participating in the activities.

Tell the women you will immunize the babies now (if immunizations are done after the group visit)

Final step
If any mothers need individual help, private exams, blood draws, injections, or treatments—instruct them what to do next.

After the mothers have gone, be sure to complete the PNC register.

After each group visit:

1. Politely encourage women to leave the group space.
2. Follow up on any pending issues for the mother or baby.
3. Co-facilitators complete the debrief tool together.
4. Complete documentation in the ANC or PNC register.
5. Put GANC supplies in a secure storage place.
6. Reset the arrangement of the room, if necessary.

Additional activities (use as needed)

The Coming and Going of the Rain: In this activity of the group will recreate the sound of coming and going of the rain. Stand and move to the space in the center of the group.

Say: “We can make the sound of the rain together. I’ll rub my hands together like this [demonstrate], and when I look at you—you do the same. Keep doing this action until I turn around to you again and show you a new action.” Slowly turn in place, making eye contact with each member of the group until she rubs her palms together.

Once you see that all the women are rubbing their hands together, make eye contact with the first woman who started and change her action to the next—lightly clapping hands. All the other women continue the first action (rubbing palms together) until you make eye contact with each of them as you turn in a circle. Each woman changes to lightly clapping hands.

Continue the same through each of the following actions:

1. Slap hands on thighs
2. Pound feet on the ground
3. Back to slapping hands on thighs
4. Back to lightly clapping hands
5. Back to rubbing hands together
6. Back to quiet—hold up both hands, palms out, to show you’re not making any noise

The big wind blows: Remove your chair from the circle, so there are now one fewer seats than the number of participants in the room. Stand in the middle of the circle and say something like, “the big wind blows for anyone who has a sister,” at which point everyone who has a sister has to leave her seat and find a new seat. One person is left without a seat and is now standing in the middle. The person in the middle chooses another characteristic and says something like, “the big wind blows for anyone who speaks more than one language” at which point everyone who is multilingual has to leave their seat and find a new seat, leaving a new person in the middle. Repeat as many times as you wish. This is a good game to recognize diversity in a fun and safe way.

The shakeout: Everyone stands up in the circle and shakes out her left arm, right arm, left leg, then right leg, starting with 8 times for each limb, then repeats all four limbs with 4, 2, and then 1. Count out loud for full effect. Do it again, counting in another language!

Excitement sharing: Each person shares something exciting that happened to her recently. Examples are: “I’ve harvested the first peas of the year” or “My friend came to visit.” This creates a lot of positive energy and puts people in touch with each other’s lives. Make sure each person keeps her comments very brief.

Stand up if you...Start by saying, “Stand up if you...“ Choose any way to finish this sentence that will get the participants to offer up a bit of information about themselves and maybe even get them laughing. For example, “Stand up if you like green bananas” or “Stand up if you are a grandmother.” Try general questions or more specific questions related to pregnancy and motherhood. If possible, ask the women to take turns each creating a “Stand up if you...” statement.

Count to ten: An excellent game for getting a group to work together—and laugh. With everyone siting in the circle, explain that they have to count upward, from one to ten. There are a few complications, though! Only one person can say any one number. If at any moment two or more people speak simultaneously, the group has to start the whole process over again at one. Nor can the same person say two numbers in a row. It’s possible that someone will attempt to coordinate the group with hand gestures or nods—discourage this. The point is to enjoy the challenge, not necessarily get to 10.

East Africa
Preterm Birth Initiative
UCSF
University of California
San Francisco

UR UNIVERSITY OF RWANDA

rbc RWANDA BIOMEDICAL CENTER
A Healthy People. A Wealthy Nation

REPUBULIKA Y'U RWANDA
UBUMWE - UMURIMO - GUKUNDA IGIHUGU
